# A narrative review of psychiatric features of traumatic encephalopathy syndrome as conceptualized in the 20th century

**DOI:** 10.3389/fneur.2023.1214814

**Published:** 2023-07-21

**Authors:** Grant L. Iverson, Alicia Kissinger-Knox, Nathan A. Huebschmann, Rudolph J. Castellani, Andrew J. Gardner

**Affiliations:** ^1^Department of Physical Medicine and Rehabilitation, Harvard Medical School, Boston, MA, United States; ^2^Department of Physical Medicine and Rehabilitation, Spaulding Rehabilitation Hospital, Charlestown, MA, United States; ^3^Department of Physical Medicine and Rehabilitation, Schoen Adams Research Institute at Spaulding Rehabilitation, Charlestown, MA, United States; ^4^Home Base, A Red Sox Foundation and Massachusetts General Hospital Program, Charlestown, MA, United States; ^5^MassGeneral Hospital for Children Sports Concussion Program, Boston, MA, United States; ^6^NYU Grossman School of Medicine, New York University, New York, NY, United States; ^7^Department of Pathology, Northwestern University Feinberg School of Medicine, Chicago, IL, United States; ^8^Sydney School of Health Sciences, Faculty of Medicine and Health, The University of Sydney, Camperdown, NSW, Australia

**Keywords:** concussion, traumatic brain injury, chronic traumatic encephalopathy, boxing, athletes, depression

## Abstract

**Introduction:**

Some ultra-high exposure boxers from the 20th century suffered from neurological problems characterized by slurred speech, personality changes (e.g., childishness or aggressiveness), and frank gait and coordination problems, with some noted to have progressive Parkinsonian-like signs. Varying degrees of cognitive impairment were also described, with some experiencing moderate to severe dementia. The onset of the neurological problems often began while they were young men and still actively fighting. More recently, traumatic encephalopathy syndrome (TES) has been proposed to be present in athletes who have a history of contact (e.g., soccer) and collision sport participation (e.g., American-style football). The characterization of TES has incorporated a much broader description than the neurological problems described in boxers from the 20th century. Some have considered TES to include depression, suicidality, anxiety, and substance abuse.

**Purpose:**

We carefully re-examined the published clinical literature of boxing cases from the 20th century to determine whether there is evidence to support conceptualizing psychiatric problems as being diagnostic clinical features of TES.

**Methods:**

We reviewed clinical descriptions from 155 current and former boxers described in 21 articles published between 1928 and 1999.

**Results:**

More than one third of cases (34.8%) had a psychiatric, neuropsychiatric, or neurobehavioral problem described in their case histories. However, only 6.5% of the cases were described as primarily psychiatric or neuropsychiatric in nature. The percentages documented as having specific psychiatric problems were as follows: depression = 11.0%, suicidality = 0.6%, anxiety = 3.9%, anger control problems = 20.0%, paranoia/suspiciousness = 11.6%, and personality change = 25.2%.

**Discussion:**

We conclude that depression, suicidality (i.e., suicidal ideation, intent, or planning), and anxiety were not considered to be clinical features of TES during the 20th century. The present review supports the decision of the consensus group to remove mood and anxiety disorders, and suicidality, from the new 2021 consensus core diagnostic criteria for TES. More research is needed to determine if anger dyscontrol is a core feature of TES with a clear clinicopathological association. The present findings, combined with a recently published large clinicopathological association study, suggest that mood and anxiety disorders are not characteristic of TES and they are not associated with chronic traumatic encephalopathy neuropathologic change.

## 1. Introduction

The purpose of this review is to carefully re-examine the published clinical literature on chronic traumatic encephalopathy (CTE) from the 20th century to determine whether there is evidence to support conceptualizing psychiatric problems as being diagnostic clinical features. We identified 155 case descriptions of boxers and former boxers from 21 published studies (1–21). Those articles are presented in Table 1. These articles were identified exclusively from three previously published narrative and systematic reviews (27–29). The clinical descriptions contained in these published articles are summarized in the Supplementary material. Our primary focus for this review was on whether depression, suicidality, and problems with anxiety were described as features of the clinical condition during the 20th century. These mental health problems have been assumed to be characteristic of CTE over the past 10–15 years.

**Table 1:** Articles with cases included in this review.

Article	Number of cases
Martland (1928) (1)	1
Parker (1934) (2)	3
Critchley (1949) (3)	7
Raevuori-Nallinmaa (1950) (4)	2
Critchley (1957) (5)	7
Neubuerger, Sinton, and Denst (1959) (6)	2
Courville (1962) (7)	1
Spillane (1962) (8)	5
Mawdsley and Ferguson (1963) (9)	10
Payne (1968) (10)	6
Roberts (1969) (11)	11
Johnson (1969) (12)	15
Corsellis, Bruton, and Freeman-Browne (1973) (13)	15
Harvey and Newsome Davis (1974) (14)	1
Kaste et al. (1982) (15)	14
Casson et al. (1984) (16)	18
Hof et al. (1992) (17)	3
Jordan, Kanik, Horwich et al. (1995) (18)	1
Jordan et al. (1997) (19)	30
Geddes, Vowles, Nicoll et al. (1999) (20)	4*
Newell and Drachman (1999) (21)	1

There were 26 articles that included 163 people who were initially identified for this review (1–26); however some cases presented were duplicates and some were not boxers. *These articles included cases that were duplicates or cases that were not boxers (or former athletes). Duplicates included two cases from Corsellis and Brierley (1959) (22) (cases #21 and #22; see Supplementary material) (22), who were also included in Corsellis et al. (1973) (cases #82 and 85) (13). The cases from Corsellis and Brierley (1959) (22) are included in the Supplementary material but they are not included in the statistical analyses for this paper; more clinical details from the 1973 article were provided relating to these cases. A case presented in Geddes et al. (1996) (#127) (23) was also a duplicate in Geddes et al. (1999) (#159) (20). The Geddes et al. (1999) (20) case was included in the statistical analyses. For those who were not boxers, two cases included women, one who experienced prolonged interpersonal violence (case #121) (from Roberts et al., 1990) (25) and another who had history of Autism and severe head banging behavior (case #122). Additionally, there were two men, one described as having intellectual disability and an intractable seizure disorder in Geddes et al. (1999) (20) (case #162) and another who was diagnosed with Autism and severe head banging behavior (case #161). Finally, there was also a case described as an ‘achondroplastic dwarf’ who was a circus clown with severe alcoholism and multiple head injuries in Williams and Tannenberg, 1996 (case #128) (26). These eight aforementioned cases are included in the supplement, but they are not included in the statistical analyses. Johnson (1969) (12) states that 10 cases were already reported by Mawdsley and Ferguson (1963) (9) and were assessed 4–5 years later. However, he does not state which 10 cases these are. Therefore, we assume that some of these cases are not unique cases—they are follow-up cases from Mawdsley and Ferguson. Therefore, in summary, case numbers #21, 22, 121, 122, 127, 128, 161, and 162 (from the Supplementary material) were not included in the statistical analyses, leaving a total of 155 boxers for analysis (from 21 articles).

There have been fundamental and persistent misunderstandings regarding CTE over the past decade. Contributing to this misunderstanding has been the terminology. The same term ‘CTE’ has been used to describe both postmortem microscopic neuropathology and broad range of antemortem psychiatric, neuropsychiatric, and neurological clinical symptoms, signs, and conditions (27, 30, 31). Some researchers have emphasized the importance of separating, not conflating, the neuropathology from the putative clinical disorder (32, 33), and as such, the terms ‘CTE neuropathologic change’ (CTE-NC) (34) or ‘CTE neuropathology’ (32, 33) have been recommended for use as opposed to referring to both the neuropathology and purported clinical features with the same term—CTE. In this paper, we will clearly differentiate the postmortem pathology using terms like CTE-NC or CTE neuropathology.

This paper is divided into six sections. First, the introduction (above). Second, we provide some historical background on the clinical condition based on articles published in the 20th century. Third, we contrast that literature with studies published this century, between 2005 and the present. We also compare and contrast the 2014 preliminary research criteria for traumatic encephalopathy syndrome (TES) (35) with the new 2021 consensus criteria for TES (36). The 2014 criteria (35) had a strong emphasis on mental health problems and specific psychiatric disorders—and this emphasis fundamentally changed in the 2021 consensus criteria for TES (36). Fourth, we review the psychiatric features of the 155 cases of boxers described in the published literature during the 20th century. Fifth, implications for drawing conclusions about associations between postmortem neuropathology and psychiatric symptoms and problems are discussed. The final section provides conclusions.

## 2. Historical background: 1928–2000

In the 20th century, CTE was conceptualized as a neurological condition experienced by some ultra-high exposure boxers—and in a severe form it was referred to as dementia pugilistica (37, 38). Some of these boxers had slurred speech, personality changes (e.g., childishness or aggressiveness), and frank gait and coordination problems (2, 5, 9, 11–14, 39, 40). Some were noted to have progressive Parkinsonian-like signs (2, 5, 9, 11–14, 17, 19). Damage to the brain was obvious enough that it was illustrated through clinical neurological examination, including pyramidal signs such as abnormal reflexes (e.g., hyperreflexia or occasionally depressed ‘sluggish’ reflexes) (2–6, 8, 9, 11, 13, 14, 16, 19, 21, 26, 41), including asymmetric hyperreflexia favoring the left side (2, 4, 8, 9, 11). Neuro-ophthalmological problems, including nystagmus, sluggish pupillary reflexes, optic atrophy, and restriction of upward gaze, were described (2, 8–11, 13, 16, 26). The neurological problems experienced by these men were sometimes documented in their 20s and early 30s while they were still actively fighting (2, 4–6, 9, 11–14). These men did not invariably have progressive neurological signs according to the published literature. Some authors (1–3, 7, 11, 12, 29, 38, 42, 43) described progressive motor signs as well as progressive dementia in some cases and others describing a more static or stationary neurological condition. Still others described progression to a point, followed by stationary disease [e.g., Critchley in 1957 (case 8) (5) and Johnson in 1969 (case 6) (12)].

In the late 1960s, Roberts (11) published a book entitled *Brain Damage in Boxers: A Study of the Prevalence of Traumatic Encephalopathy Among Ex-Professional Boxers*. He provided a clinical description of a random sample of 224 retired professional boxers, who competed between 1929 and 1955, and had extensive exposure to boxing over many years. He described the syndrome as predominately cerebellar or extrapyramidal, typically characterized by dysarthria and motor problems, with some cases having dementia. He reported that 11% of his random sample were deemed to have a mild form of the syndrome and 6% were considered to have a moderate-to-severe form of the syndrome. He did not consider the large majority of boxers to have the syndrome. Those who participated in a large number of professional fights, and those who fought prior to World War II, were considerably more likely to have the condition.

One article, by Johnson in 1969, was entitled ‘Organic Psychosyndromes Due to Boxing’ (12). Johnson noted that not much had been written about the psychiatric problems experienced by boxers. His article was based on clinical examinations of 17 boxers who presented to the hospital with neuropsychiatric symptoms thought to be connected to their boxing career, with 10 of those cases previously described by Mawdsley and Ferguson in 1963 (9). Sixteen of the 17 cases were former professional boxers, with 200–300 professional fights, and many had permanent facial disfigurement. He described one case as an amateur who ‘developed anxiety symptoms following a domestic crisis and falsely attributed these to insidious “punch drunkenness.” All investigations were normal and at follow-up he was symptom free’ (page 45). Johnson noted that the neurological condition was progressive in eight cases and not progressive in the other eight cases. He wrote that ‘the accepted title given to this condition by Critchley (1957) implies that progression is inevitable. Certainly this is not so in all cases. Cases 3, 5, 6, 7, 9, 10, 13, 14 had not deteriorated over the years of the follow-up of this study, and indeed according to relatives’ accounts they had deteriorated little since the end of their fighting careers’ (page 52). He considered the main psychiatric clinical syndromes to be (i) ‘chronic amnestic state’ (i.e., memory impairment, usually not progressive), (ii) ‘dementia’ (with mental torpor and flat affective responses), (iii) ‘morbid jealousy syndrome’ (in relation to their wives), (iv) ‘rage reactions in personality disorder’ (with impulsive acts of violence, often while intoxicated), and (v) psychosis. Two of his cases experienced chronic psychosis (and both had a history of mental illness in a first-degree relative). He concluded by stating that an ‘organic psychosyndrome was manifest in a chronic amnesic state, morbid jealousy reactions, psychosis and “explosive” personality disorder in varying combination in 14 cases’ (page 52) (12).

At the beginning of this century, Jordan published a review of the 20th century literature relating to what he termed ‘chronic traumatic brain injury’ [CTBI (44);]. He included a broader description of chronic brain damage in boxers, with CTE and dementia being the most severe. He described CTBI in a relatively mild form as involving mild dysarthria and difficulty with balance, while those with more extensive neurological problems experience ataxia, spasticity, and Parkinsonism. He reported that cognitive impairment could range from mild to dementia, and diverse behavioral changes might include irritability, euphoria or hypomania, disinhibition, impaired insight, paranoia, and violent outbursts. Jordan noted that it was unclear whether CTBI in boxers reflected a progressive neurodegenerative disease, whether it reflected the aging process superimposed on more static TBI-related neurological injury, or both.

## 3. The modern era: 2005–2021

There has been a large amount of research on CTE-NC in the past 17 years. Postmortem case studies of this neuropathology (30, 45–49) and a comprehensive review of the literature (27) were published between 2005 and 2012. A larger postmortem case series was published in 2013 (31). In 2014, a new and comprehensive set of preliminary clinical diagnostic criteria were published, designed to identify traumatic encephalopathy syndrome (TES) in living research subjects (35). It is during this time, from 2005–2015, when CTE (including CTE-NC), and TES, were conceptualized very differently than they were in the 20th century. Virtually any psychosocial, psychiatric, or neurological symptom or problem that a person experienced during life was attributed, directly or indirectly, to ‘CTE’ (30, 31, 35, 45, 48, 50–55), and thus by inference, to specific neuropathology identified after death (i.e., CTE-NC). This is illustrated in Table 2.

**Table 2:** Psychiatric, psychological, psychosocial, and neurological signs, symptoms, and disorders that have been described as part of the clinical features of traumatic encephalopathy syndrome and CTE between 2009 and 2014.

Psychiatric/Psychological symptoms
Symptoms of depression (e.g., feeling overly sad or hopeless) and anxiety (e.g., anxious mood or excessive fears) (30, 31, 35, 50)
Specific psychiatric diagnoses
Major depressive disorder (35)
Persistent depressive disorder (35)
Generalized anxiety disorder (35)
Obsessive–compulsive disorder (35)
Intermittent explosive disorder (35)
Suicidality
Suicidal ideation, suicide attempts, or suicide as a manner of death (31, 35, 50–55)
Apathy and Anhedonia
Apathy (35)
Loss of interest in usual activities (35)
Loss of motivation and emotions (35)
Paranoia and suspiciousness
Paranoia (35)
Excessive, unwarranted jealousy (35)
Psychosocial problems, substance abuse, and impulse control problems
Personality changes, anger control problems, and violence (30, 31, 35, 50)
Poor financial decisions, financial problems, and bankruptcy (30)
Gambling (35)
Excessive shopping or unusual purchases (35)
Increased or unusual sexual activity (35)
Marital problems, separation, and divorce (55)
Substance abuse (35)
General health problems
Headaches (27, 30, 31, 35)
Generalized body aches and pain (30)
Insomnia (55)
Motor signs and neurological problems
Dysarthria (35)
Dysgraphia (35)
Bradykinesia (35)
Rigidity (35)
Gait disturbance (35)
Falls (35)
Parkinsonism (31, 35, 45, 50)
Motor neuron disease including amyotrophic lateral sclerosis (ALS) (48)
Cognitive impairment and dementia
Mild cognitive impairment (30, 31, 35, 50)
Dementia (30, 31, 35, 50)

### 3.1. Consensus criteria for defining CTE neuropathologic change (CTE-NC) in 2016 and 2021

In 2016, a consensus group published preliminary criteria for defining the microscopic neuropathology of CTE-NC and establishing the research methodology for case identification (56). The consensus group defined a descriptive *pathognomonic* lesion based on microscopic assessment of p-tau immunohistochemical stains as follows: ‘p-tau aggregates in neurons, astrocytes, and cell processes around small vessels in an irregular pattern at the depths of the cortical sulci’ (page 81). The authors described these preliminary criteria as a first step toward the development of validated criteria for CTE-NC and they reported that future researchers could address the potential contribution of p-tau and other pathologies to clinical signs or symptoms. In 2021, the consensus group published updated and revised consensus criteria for CTE-NC (57). The revised *pathognomonic lesion* was described as ‘p-tau aggregates in neurons, with or without thorn-shaped astrocytes, at the depth of a sulcus around a small blood vessel, deep in the parenchyma, and not restricted to the subpial or superficial region of the sulcus’ (page 217). The consensus group who published the preliminary criteria for CTE-NC in 2016 (56) and the revised criteria in 2021 (57) did not attempt to define clinical signs, symptoms, or a clinical syndrome. They encouraged future researchers to determine if there is an association between the postmortem neuropathology and specific neurological problems during a person’s lifetime.

### 3.2. Important knowledge gaps as of 2021

During 2021, two major efforts were published: revised consensus criteria for CTE-NC (57) and the first consensus criteria for TES (36). Prior to 2021, there were large gaps in knowledge that were described in critical reviews (32, 33, 58)—and many major knowledge gaps persist to the present day. The prevalence rates of the neuropathology, interobserver variability in CTE-NC interpretation, and the putative clinical features were unknown. It was not known whether, or the extent to which, either the presumed neuropathology or the presumed clinical features are inexorably progressive. It was not known whether, or the extent to which, the *emergence*, *course, or severity* of clinical signs and symptoms are caused directly or indirectly by CTE neuropathology. Moreover, prior to 2021, there were no agreed upon, or validated, criteria for diagnosing CTE or TES in a living person. There were several attempts to create clinical diagnostic criteria for CTE and TES, published between 2013 and 2018 (29, 35, 59–61), but none of these criteria were rigorously researched or validated. All of the aforementioned knowledge gaps remain in 2023.

### 3.3. The 2021 consensus criteria for traumatic encephalopathy syndrome

New consensus criteria for TES were published in 2021 (36). They were developed through a Delphi process by a multidisciplinary group of clinicians and scientists, led by researchers from Boston University. They used the preliminary 2014 research criteria (35) as the foundation for their work. Those 2014 criteria were broad, heavily focused on psychiatric problems, and several studies illustrated problems with applying the diagnostic criteria and their associated risk for misdiagnosis (62–65). The core features of the 2014 criteria included depression (e.g., major depressive disorder), anger dyscontrol (e.g., intermittent explosive disorder), and cognitive impairment (e.g., mild cognitive impairment or dementia). For the 2021 consensus criteria for TES (36), no parts of the 2014 criteria (35) were retained in their original form. Importantly, having major depressive disorder or intermittent explosive disorder is no longer allowed to meet core criteria for TES, and anxiety disorders and suicidality are no longer considered to be supportive diagnostic features. The two sets of criteria for TES are compared in Table 3. Twelve hypothetical cases of former contact and collision sport athletes, who developed psychiatric problems after their sporting careers, are presented in Table 4. These cases illustrate how people presenting with a diverse range of psychiatric and psychosocial difficulties could have been diagnosed with TES using the preliminary 2014 criteria but none of these cases would be considered to have TES based on the 2021 consensus criteria.

**Table 3:** Comparing the 2014 preliminary research diagnostic criteria to the 2021 consensus criteria for traumatic encephalopathy syndrome.

	2021 Consensus Criteria (36)	2014 Preliminary Research Criteria (35)
Exposure Criteria	Substantial exposure to repetitive head impacts (e.g., 5 or more years of football, with at least 2 years at the high school level). Other types of exposures are included but are not clearly defined (e.g., military occupational exposures to low-level blast). Comment: Mild, moderate, and severe TBIs are not considered to be part of the exposure criteria. The exposure criterion does not consider concussions and it is much less/lower than the criterion from 2014.	History of multiple impacts to the head (or to the body resulting in impulsive force transmitted to the head) from a variety of sources, including: (i) a minimum of four or more mild TBIs; (ii) a minimum of two or more moderate–severe TBIs, and/or (iii) subconcussive trauma from playing sports, military service, or domestic violence. For sports, a minimum of 6 years was required, including at least 2 years at the college level or higher.
Core Diagnostic Features	‘Diagnosis of TES requires (1) substantial exposure to repetitive head impacts (RHIs) from contact sports, military service, or other causes (2); core clinical features of cognitive impairment (in episodic memory and/or executive functioning), neurobehavioral dysregulation, or both (3); a progressive course; and (4) that the clinical features are not fully accounted for by any other neurologic, psychiatric, or medical conditions’ (pg. 848) (36). Comment: The authors stated that intermittent explosive disorder *does not* meet the criterion for neurobehavioral dysregulation.	For diagnosis, *one of three core features* must be present: (i) difficulties with cognition (subjective and objectively measured); (ii) being emotionally explosive, physically violent, or verbally violent; and/or (iii) feeling overly sad, depressed, and/or hopeless. Comment: The authors stated that a formal diagnosis of intermittent explosive disorder meets the second core criterion but is not necessary for diagnosis. They also stated that diagnoses of major depressive disorder or persistent depressive disorder meet the third core criterion but are not necessary for diagnosis (35). The 2021 criteria are substantially different and no longer include psychiatric disorders as core or supportive diagnostic features.
Supportive Features (2021)/Supportive Diagnostic Criteria (2014)	Supportive features are *not required* for diagnosis. The three supportive features are (i) delayed onset of symptoms, (ii) motor signs [e.g., a diverse range of Parkinsonian signs (e.g., bradykinesia, tremor, gait disorder), upper motor neuron signs (e.g., spasticity or hyperreflexia), lower motor neuron signs (e.g., fasciculations and muscle atrophy), and/or amyotrophic lateral sclerosis], and (iii) psychiatric features (e.g., a diverse range of psychiatric problems, occurring singly or in combination, that are persistent or progressive, including anxiety disorders, depressive disorders, apathy, and paranoia) (36).	*A minimum of two of the following nine supportive criteria* must be present: (i) impulsivity (e.g., excessive gambling, substance abuse, or excessive shopping); (ii) anxious mood, agitation, excessive fears, or obsessive or compulsive behavior (or both); (iii) apathy (e.g., loss of interest in usual activities, loss of motivation and emotions); (iv) paranoia (e.g., delusional beliefs or unwanted jealousy); (v) suicidality (history of suicidal thoughts or attempts); (vi) headache (at least once per month for six months), (vii) motor signs (e.g., dysarthria, bradykinesia, tremor, gait disturbance, and/or other features of Parkinsonism), (viii) documented decline in function or symptoms for at least one year; and (ix) delayed onset (onset of clinical features at least two years after the exposure to repetitive head impacts) (35).
Must be a Progressive Clinical Condition	Yes. This is a required diagnostic feature.	No. Being progressive is one of nine possible supportive diagnostic criteria, for at least one year, and is “based upon repeated formal testing, clinician examination, or other formal measurement” (page 11)
Can be Diagnosed Based Entirely on Mental Health Problems	No. Comment: This is a major change from the 2014 criteria and how TES/CTE has been described in the literature between 2009 and 2021.	Yes. Comment: For example, former college football, rugby, or hockey players who develop depression, anxiety, and suicidality any time during middle or late adulthood could (would) meet criteria for TES.

**Table 4:** Twelve hypothetical examples of psychiatric presentations in former college football, soccer, ice hockey, or rugby athletes that would meet 2014 (35) criteria for traumatic encephalopathy syndrome (TES) but not the 2021 consensus criteria (36) for TES.

	One Core Feature is Required (2014 criteria)		Two or More Supportive Features are Required for Diagnosis (2014 criteria)								
Gender Age	Depression	Anger Dyscontrol	Impulsivity	Anxiety	Suicidality	Apathy	Paranoia	Headaches Per Month	Delayed Onset	Progressive (one or more years)	# of Supportive Features
Woman 36	Major Depressive Disorder	--	Alcohol Abuse	Anxious Mood	Thoughts	--	--	--	✓	✓	5
Man 51	Feeling Hopeless	--	Excessive Gambling	Agitation	Thoughts/ Attempt	--	--	1–3	✓	--	5
Woman 53	Feeling Overly Sad	--	Alcohol Abuse	Generalized Anxiety Disorder	Thoughts	--	--	2–5	✓	✓	6
Man 38	Feeling Overly Sad	--	Alcohol Abuse	Generalized Anxiety Disorder	--	--	--	5–10	✓	✓	5
Man 67	Feeling Depressed	--	--	--	--	Loss of Interest and Low Motivation	--	--	✓	--	2
Man 42	Major Depressive Disorder	Intermittent Explosive Disorder	--	Anxious Mood	Thoughts	--	--	1–3	✓	✓	5
Woman 33	Feeling Depressed	--	--	Obsessive–Compulsive Disorder	--	--	--	--	✓	--	2
Man 58	Major Depressive Disorder	--	Alcohol Abuse	Agitation	--	--	Excessive/ Unwanted Jealousy	--	✓	✓	5
Man 28	--	Intermittent Explosive Disorder	Alcohol Abuse	Agitation	Thoughts	--	--	--	--	--	3
Man 31	Feeling Hopeless	Intermittent Explosive Disorder	--	Anxious Mood	Thoughts	--	--	5–7	--	✓	4
Man 75	Persistent Depressive Disorder	--	--	Agitation	Thoughts	Loss of Interest and Low Motivation	--	--	✓	--	4
Woman 63	Persistent Depressive Disorder	--	Excessive Gambling	Anxious Mood	--	--	--	--	✓	--	3

For former athletes, the exposure criteria in 2014 based on years of play for being at risk for TES is a minimum of 6 years, including at least 2 years at the college level or higher. Any former college athletes who develop depression, for example, anytime during adulthood (after their playing years, such as age 30 through death), meet the criteria for ‘delayed onset.’ Therefore, as written, only one additional supportive feature is required. In fact, as written, if a person developed depression at any point during mid or late adulthood, and that depression progressively worsened over the course of more than a year, than that person would meet 2014 criteria for TES.

## 4. Review of the psychiatric features of 155 cases of boxers from the 20th century

We initially identified 26 published articles from the 20th century (1–26) that included 163 cases, 158 of whom were men and current or former boxers—although three were duplicates leaving a sample size of 155 from 21 published articles (see Table 1; Supplementary material). Two authors (NAH and AKK) reviewed each article, extracted quotes relating to the clinical features, and coded the cases based on clinical features (see Supplementary material). The psychiatric and neuropsychiatric clinical features from those cases are summarized in Table 5.

**Table 5:** Clinical features of the 155 cases.

Clinical Feature	Present		Not present		Unknown/Not mentioned/Missing	
	f	%	f	%	f	%
Depression	17	11.0	22	14.2	116	74.8
Suicidality	1	0.6	18	11.6	136	87.7
Anxiety	6	3.9	18	11.6	131	84.5
Depression, Suicidality, or Anxiety	23	14.8	22	14.2	110	71.0
Anger Control Problem	31	20.0	21	13.5	103	66.5
Paranoia/Suspiciousness	18	11.6	19	12.3	118	76.1
Personality Change	39	25.2	30	19.4	86	55.5
Paranoia/Suspiciousness/Personality Change	40	25.8	30	19.4	85	54.8
Substance Use (Alcohol)	43	27.7	43	27.7	69	44.5
Any Psychiatric or Neuropsychiatric Problem*	54	34.8	29	18.7	72	46.5
Considered Primarily Psychiatric	10	6.5	145	93.5	--	--
Progressive Course	49	31.6	16	10.3	90	58.1

f = frequency, % = percentage, and *having any psychiatric or neuropsychiatric problem includes depression, suicidality, anxiety, paranoia, suspiciousness, anger control problems, or personality change.

More than one third of cases (34.8%) had a psychiatric, neuropsychiatric, or neurobehavioral problem described in their case histories. However, only 6.5% of the cases were described as primarily psychiatric/neuropsychiatric in nature. We did not find evidence that the authors of these articles conceptualized these cases as having predominantly a psychiatric condition such as depression or an anxiety disorder. There were only 14.8% of the cases who had depression, suicidality, or anxiety documented. Quotes from articles describing cases as having major psychiatric problems are reprinted in Table 6.

**Table 6:** Examples of clinical descriptions of boxers with psychiatric problems.

Critchley (1957) (5): “Simple fatuous cheerfulness is, however, the commonest prevailing mood, though sometimes there is depression with a paranoid coloring” (page 359).
Spillane (1962) (8): “The fourth patient, aged 33, is in excellent physical health, there is no abnormality on neurological examination, but he is aggressive and violent, especially when he has been drinking” (page 1208).
Payne (1968) (10): “He drank alcohol but the amount is unknown. In 1914 he was admitted to hospital with ‘Manic Depressive Psychosis’ and thereafter he spent many years in mental institutions. He had a guilt complex about his mis-spent youth” (page 175).
Johnson, 1969 (12): “An organic psychosyndrome was manifest in a chronic amnesic state, morbid jealousy reactions, psychosis and “explosive” personality disorder in varying combinations in 14 cases” (page 52). “Persistent accusations against the wife’s supposed sexual infidelity led to the primary psychiatric referral in five cases (1, 6, 8, 9, 10). The morbid jealously persisted in all cases at follow-up, except in Case 1” (page 48). “At 45 years he became impotent, although making excessive sexual demands upon his wife. He then began to accuse her of being pregnant by his son, brother, nephew and later his neighbors. He became increasingly querulous and argumentative, and was finally admitted to a local psychiatric unit where he was diagnosed as ‘paranoia due to brain injury’…Over the past fifteen years his family have tolerated his incessant suspicion and accusations about his wife’s supposed infidelity with neighbors. Owing to his quarrelsome attitudes he has not worked for ten years” (page 48). “A persistent psychosis was present…Case 6… had a chronic paranoid psychosis with a chronic amnesic state due to underlying brain damage” (pages 48–49). “…has a sensitive, querulous, suspicious type of personality and had always been ‘touchy.’ His development of a morbid jealously syndrome in late life was seen as a personality reaction related to brain damage” (page 48). “Severe personality disorders were present in four cases (1, 8, 9, 10). In three of these (1, 9, 10) impulsive aggressive behavior was prominent as a life-long trait and justified the description of ‘explosive, psychopathic personality.’ Rage reaction, uncontrolled outbursts of anger and violence, were prominent in the case histories of these men after their boxing careers and were attributed by them to their decreased tolerance to alcohol. All three cases had had numerous emergency psychiatric admissions for impulsive acts of violence, of short duration” (page 48). “Case 8 had an episode of endogenous depression at the age of 30 years which responded to E.C.T.” (page 49).
Roberts (1969) (11): “This man, aged 60,… had been a patient in a mental hospital for seven [years]” (pages 27–28). “The death of his wife precipitated a paranoid delusional illness which necessitated his admission, finally for long-term care, to a mental hospital” (page 28).
Corsellis, Bruton, and Freeman-Browne (1973) (13): “This man was a patient in a psychiatric hospital for the last seven years of his life… He tried to kill himself when aged 65 and was admitted to a psychiatric hospital” (page 282).
Geddes, Vowles, Nicoll, and Révész (1999) (20): “At the age of 20 he was admitted to a psychiatric hospital with a diagnosis of paranoid schizophrenia which responded to major tranquillizers. He was readmitted with an acute psychotic illness at the age of 25, this type more depressive in nature, and then again 2 years later. He died unexpectedly the following year during a grand mal seizure. He had no history of a serious head injury during his boxing career” (page 172).

### 4.1. Depression, anxiety, and suicidality

Depression was reported in some cases from the 20th century (11.0%, see Table 5) (3, 8–12, 14, 19). In general, depression was not reported to be the primary problem, or the only problem, but rather it was reported in the context of a broader clinical history and there was an emphasis on the neurological condition and not mental health (see Supplementary material). We did not find evidence to suggest that authors in the 20th century conceptualized CTE, TES, or dementia pugilistica as a primary depressive disorder or involving a neurologically-mediated secondary depressive disorder.

It is important to keep in mind that former contact, collision, or contact sport athletes, or high exposure military veterans, might develop depression for reasons that are similar to those experienced by people in the general population. Depression in the general population is associated with genetics (66), adverse events in childhood (67, 68), and current life stressors (69). It is also associated with chronic pain (70), headaches and migraines (71, 72), chronic insomnia (73), and sleep apnea (74). A systematic review concluded that there might be a bidirectional relationship between symptoms of depression and cardiovascular health (75). It is also well established that there is an association between diabetes and depression (76), and although the mechanisms underlying that association are not well understood, a recent systematic review and meta-analysis suggests that there might be a bidirectional longitudinal association (77). It is also essential to appreciate that neurological problems and diseases in older adults are associated with depression, such as Parkinson’s disease (78), mild cognitive impairment (79), and Alzheimer’s disease (80). Therefore, it is apparent that possible associations between repetitive mild neurotrauma, later in life depression, and the presence of CTE-NC might be influenced by many other factors.

We did not find evidence in our review that anxiety, or anxiety disorders—such as generalized anxiety disorder or obsessive–compulsive disorder—were conceptualized as being a core or supportive clinical feature of TES during the 20th century. Some form of anxiety was described in only 3.9% of cases (2–4, 6, 8). When it was described, it was usually included in the case histories of former boxers who clearly had neurological problems (see Supplementary material). For example: “At times he is anxious or depressed….The positive neurological signs consisted of slow slurred speech, unsteady gait, moderate right-sided hemiparesis, and partial right optic atrophy. There was no dysphasia, he dragged his right foot when walking, and all movements were performed slowly and carefully. There was moderate ataxia on formal testing in all limbs, more marked on the right. There was no diplopia, ocular movements were normal…” (8) (page 1,206). Another example was as follows: “…of late he had been getting nervous. The main feature in his case was a spastic, stammering type of dysarthria and a habit of talking without opening his mouth sufficiently” (page 136) (5).

We found no evidence in our review that suicidality (i.e., suicidal ideation, intent, or planning), or suicide as a manner of death, were conceptualized as being core or supportive clinical features of TES during the 20th century. Similarly, in their review of the world literature on CTE, McKee and colleagues, in 2009, did not consider suicidality or suicide to be a clinical feature (27). We identified only one case (0.6%) in the literature described as experiencing suicidality—and that person was part of the case series described by Corsellis and colleagues in 1973 (13). Notably, when the postmortem brain tissue from that case series was reexamined using the modern definition of CTE-NC (56), by Goldfinger and colleagues (81), the man who had suicidality documented did not have CTE-NC. He had Lewy body dementia, accompanied by aging-related tau astrogliopathy. Many studies in recent years have examined suicidality or suicide as a manner of death (82–90), some with former amateur athletes (82–85) and some with former professional athletes (86–90), and none of these studies found an association between playing contact or collision sports during youth or adulthood and future risk for suicidality or suicide.

### 4.2. Anger attacks, aggressiveness, and intermittent explosive disorder

In studies published last century, some current and former boxers had documented anger control problems and violent behavior during their lifetime (5, 9, 10, 12–14). Some authors speculated that anger dyscontrol and aggressiveness might have represented longstanding personality or behavioral characteristics in some boxers (6, 11, 13). In the present review, anger dyscontrol was identified in 19.0% of the cases (see Table 5). The anger dyscontrol documented in their case histories co-occurred with obvious neurological signs of damage to their brains, such as dysarthric speech, gait problems, and Parkinsonism, and virtually all had cognitive impairment or dementia (9–13) (see Supplementary material).

The preliminary 2014 criteria indicated that a diagnosis of intermittent explosive disorder would fulfill the core ‘behavioral’ clinical criterion for TES. This was problematic because intermittent explosive disorder is a psychiatric disorder that tends to emerge in adolescence and early adulthood, and people with intermittent explosive disorder often experience co-occurring mood disorders, anxiety disorders, and substance use disorders (91). Therefore, a person with primary neurodevelopmental intermittent explosive disorder could be misdiagnosed as having TES based on the 2014 criteria, as illustrated in one study (63). In the 2014 criteria, both depression and any form of anger control problems were considered to be core diagnostic features. This was also problematic because depression and anger attacks tend to co-occur in men in the general population (92, 93), reducing the specificity of these criteria and increasing the risk for misdiagnosis.

The 2021 consensus group disagreed with including intermittent explosive disorder as part of the criteria for TES, and the new consensus criteria explicitly state that it should not be considered part of the diagnostic criteria for TES (36). In contrast, they describe ‘neurobehavioral dysregulation’ as one of the core diagnostic features for TES. Neurobehavioral dysregulation is described as having: ‘symptoms and/or observed behaviors representing poor regulation or control of emotions and/or behavior, including (but not limited to) explosiveness, impulsivity, rage, violent outbursts, having a short fuse (exceeding what might be described as periodic episodes of minor irritability), or emotional lability (often reported as mood swings)’ (page 852) (36).

There are several reasons why neurobehavioral dysregulation might prove to be difficult to conceptualize in future studies of former contact and collision sport athletes or military veterans. It is possible that some people might have these characteristics as a longstanding part of their personality and behavior (94–101). Moreover, if so, these characteristics could be exacerbated by life stressors (102), including financial problems (103) or marital problems (103), depression (92, 93), substance abuse (104, 105), and a variety of neurological conditions and diseases such as severe TBI (106, 107), stroke (108–110), and Alzheimer’s disease (111, 112).

## 5. Implications for clinicopathological associations

In recent years, since prior to the publication of the preliminary criteria for TES in 2014, there has been an assumption that the postmortem neuropathological entity identified via immunohistochemistry, CTE-NC, has an associated clinical syndrome (or syndromes)—and efforts to validate diagnostic criteria for TES with CTE-NC have been underway for many years. Many articles have asserted that psychiatric problems, such as depression, suicidality, anxiety, and substance abuse, are a fundamental part of the clinical condition of ‘CTE,’ for which CTE-NC is the presumed neuropathological substrate (e.g., see Table 2).

**Figure 1:**
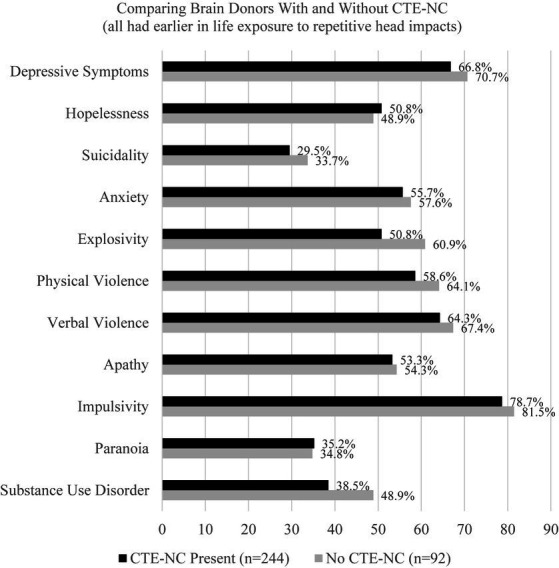
No association between having chronic traumatic encephalopathy neuropathologic change and psychiatric features in former athletes and military veterans (*N* = 336) (113). CTE-NC, chronic traumatic encephalopathy neuropathologic change. These data were derived from 336 consecutive brain donors exposed to repetitive head impacts from sports, military service, and/or physical violence, 244 (72.6%) of whom were identified as having CTE-NC and 92 did not have CTE-NC (27.4%) (113). To create this figure, data were extracted from a table on pages 9 and 10 of the online supplement for the article by Mez et al. (113). There are no statistically significant differences in the proportions of the groups that have any of these psychiatric characteristics or features based on chi square tests.

In 2021, a large-scale clinicopathological study designed to validate the 2014 criteria for TES was published, and the authors concluded that the TES criteria had high sensitivity for CTE-NC, but low specificity, and the mood (i.e., depression) and behavior (i.e., anger dyscontrol) symptoms of TES were not associated with CTE-NC (113). That study was extraordinarily important because (i) it was large, involving 336 brain donors who were exposed to repetitive head impacts from sports, military service, and/or physical violence, (ii) the neuropathology was rated without knowledge of the clinical information, and (iii) the clinical diagnostic criteria for TES were rated without knowledge of the neuropathology. Of the 336 brain donors, 244 (72.6%) were identified as having CTE-NC and 92 (27.4%) did not have CTE-NC. The individual psychiatric features that we rated in the present article, from the 155 boxers included in published articles from the 20th century, were also included in this clinicopathological correlation and validation study with brain donors from the past few years (113). There was no difference between those with CTE-NC and those who did not have the neuropathology in depressive symptoms, hopelessness, suicidality, anxiety, explosivity, physical violence, verbal violence, apathy, impulsivity, paranoia, or substance abuse (see supplementary Table S5, included in the online supplement from the article by Mez et al.) (113). This is illustrated in Figure 1.

The findings from the present study, and from the clinicopathological validation and association study summarized in Figure 1 (113), have implications for the new consensus criteria for TES. The authors of the consensus criteria wrote that psychiatric features “were not included as core features but reserved as supportive features that are used in determining levels of certainty for CTE pathology” (page 856) (36). These psychiatric features include anxiety, apathy, depression (e.g., “feeling overly sad, dysphoric, or hopeless, with or without a history of suicidal thoughts or attempts” page 856), and paranoia. In other words, if a research subject or clinical patient has one of those four psychiatric features, this is supposed to “increase levels of certainty” that the person harbors CTE-NC (that ultimately will be identifiable after death). However, according to the large-scale clinicopathological validation study there is no association between any of those psychiatric features and having CTE-NC after death (113) (see Figure 1). Therefore, there is a major problem with the new TES consensus criteria when used to try to draw an inference about the likelihood of having CTE-NC based on the presence (or absence) of these psychiatric features. Given that there is no association between the psychiatric features included in the 2021 TES consensus criteria (36) and the postmortem presence of CTE-NC (113), a future revision of the TES consensus criteria should consider dropping psychiatric features as factors that are assumed to increase the level of certainty that a person has CTE-NC.

## 6. Conclusion

During the 20th century, TES was described as a neurological condition, and in a severe form it was referred to as dementia pugilistica. Many articles discussed the onset of neurological problems reflecting chronic brain damage while the boxers were still actively fighting (1–5, 11, 12, 14, 18, 21). Many authors described the neurological condition as having a progressive course (3–6, 8, 9, 13–15, 18, 21, 22), but it was also discussed throughout the literature that the course does not appear to be progressive in some cases (11, 12). It was recognized last century that some current and former boxers had psychiatric and neuropsychiatric problems such as personality changes, impulsive aggressiveness and violence, paranoia, and psychosis.

In recent years, TES was reconceptualized to include a very broad range of psychosocial and mental health problems (31, 35, 50–55) (see Table 2). The reconceptualization of depression as being a diagnostic feature of TES was new to this century (35) (see Table 2), and did not appear in the literature we reviewed from the 20th century. Importantly, the 2021 TES consensus group (36) disagreed that psychiatric disorders should be conceptualized as diagnostic clinical features of TES, as defined in the 2014 preliminary diagnostic criteria (35), and removed them from the status of core or supportive diagnostic features. Instead, the new consensus criteria indicate that psychiatric problems often can be associated with TES but are not diagnostic of it.

The present review of cases from the 20th century supports the decision of the TES consensus group (36) to remove mood and anxiety disorders, and suicidality, from the diagnostic criteria for TES. That said, the consensus group designed the TES criteria so that having a psychiatric problem is assumed to increase the level of certainty that the person harbors CTE-NC. However, the best available evidence suggests that there is no association between the TES psychiatric features and having CTE-NC, as illustrated in Figure 1 (113). Therefore, the present review of cases from the 20th century, combined with a recently published large clinicopathological association study (113), suggests that depression, suicidality, anxiety, and substance abuse disorders are not characteristic features of TES and they are not associated with having the underlying neuropathology conceptualized as CTE-NC.

## Author contributions

GI conceptualized and designed the review, assisted with the literature review, wrote sections of the manuscript and secured funding for the work. AK-K was the second author to review all articles and extract additional quotes for the Supplementary material, she entered data into the database and edited drafts of the manuscript. NH was the first author to review all articles and extract quotes for the Supplementary material, he designed the database and entered data and edited drafts of the manuscript. RC assisted with the literature review and edited drafts of the manuscript. AG assisted with conceptualizing the review, assisted with the literature review and extract additional quotes for the Supplementary material and edited drafts of the manuscript. All authors contributed to the article, approved the submitted version and agree to be accountable for the content of the work.

## Funding

The study was funded, as part of a program of research entitled improving the methodology for diagnosing traumatic encephalopathy syndrome (PI GI), by the Wounded Warrior Project™. This work was also funded, in part, by unrestricted philanthropic research support from the National Rugby League (AG and GI). GI acknowledges unrestricted philanthropic support from the Mooney-Reed Charitable Foundation, ImPACT Applications, Inc., the Heinz Family Foundation, Boston Bolts, and the Schoen Adams Research Institute at Spaulding Rehabilitation. None of the above entities were involved in the study design, analysis, interpretation, the writing of this article, or the decision to submit it for publication.

## Conflict of interest

GI serves as a scientific advisor for NanoDX^®^, Sway Operations, LLC, and Highmark, Inc. He has a clinical and consulting practice in forensic neuropsychology, including expert testimony, involving individuals who have sustained mild TBIs (including former athletes), and on the topic of suicide. He has received past research support or funding from several test publishing companies, including ImPACT Applications, Inc., CNS Vital Signs, and Psychological Assessment Resources (PAR, Inc.). He receives royalties from the sales of one neuropsychological test (WCST-64). He has received travel support and honorariums for presentations at conferences and meetings. He has received research funding as a principal investigator from the National Football League, and subcontract grant funding as a collaborator from the Harvard Integrated Program to Protect and Improve the Health of National Football League Players Association Members. RC is a collaborator on a grant funded by the National Football League to study the spectrum of concussion, including possible long-term effects. He has a consulting practice in forensic neuropathology, including expert testimony, which has involved former athletes at amateur and professional levels, and sport organizations. AG serves as a scientific advisor for hitIQ, Ltd. He has a clinical practice in neuropsychology involving individuals who have sustained sport-related concussion (including current and former athletes). He has been a contracted concussion consultant to Rugby Australia since July 2016. He has received travel funding or been reimbursed by professional sporting bodies, and commercial organizations for discussing or presenting sport-related concussion research at meetings, scientific conferences, workshops, and symposiums. Previous grant funding includes the NSW Sporting Injuries Committee, the Brain Foundation (Australia), an Australian-American Fulbright Commission Postdoctoral Award, a Hunter New England Local Health District, Research, Innovation and Partnerships Health Research & Translation Centre and Clinical Research Fellowship Scheme, and the Hunter Medical Research Institute (HMRI), supported by Jennie Thomas, and the HMRI, supported by Anne Greaves. AG is supported by a National Health and Medical Research Council (NHMRC) Investigator Grant. He acknowledges unrestricted philanthropic support from the National Rugby League for research in former professional rugby league players.

The remaining authors declare that the research was conducted in the absence of any commercial or financial relationships that could be construed as a potential conflict of interest.

## Publisher’s note

All claims expressed in this article are solely those of the authors and do not necessarily represent those of their affiliated organizations, or those of the publisher, the editors and the reviewers. Any product that may be evaluated in this article, or claim that may be made by its manufacturer, is not guaranteed or endorsed by the publisher.
