# Supplementary material for: A narrative review of psychiatric features of traumatic encephalopathy syndrome as conceptualized in the 20th century
Source: Front Neurol. 2023 Jul 21;14:1214814. doi: 10.3389/fneur.2023.1214814 (PMC10401603; doi:10.3389/fneur.2023.1214814)
Supplement: Supplementary file 1 [file Data_Sheet_1.PDF]

Frontiers in Neurology, Neurotrauma Section

Online Supplementary Material

A Narrative Review of Psychiatric Features of Traumatic Encephalopathy Syndrome as Conceptualized in the 20th Century

Grant L. Iverson, Ph.D.[1-5], Alicia Kissinger-Knox, Psy.D.[1,2,5], Nathan A. Huebschmann, BA[6], Rudolph J. Castellani, M.D.[7], & Andrew J. Gardner, Ph.D.[8]

1. Department of Physical Medicine and Rehabilitation, Harvard Medical School, Boston, MA, USA.
2. Department of Physical Medicine and Rehabilitation, Spaulding Rehabilitation Hospital, Charlestown, MA, USA.
3. Department of Physical Medicine and Rehabilitation, Schoen Adams Research Institute at Spaulding Rehabilitation, Charlestown, MA, USA.
4. Home Base, A Red Sox Foundation and Massachusetts General Hospital Program, Charlestown, MA, USA.
5. MassGeneral Hospital for Children Sports Concussion Program, Boston, MA, USA
6. NYU Grossman School of Medicine, New York University, New York, NY, USA
7. Department of Pathology, Northwestern University Feinberg School of Medicine, Chicago, Illinois, USA
8. Sydney School of Health Sciences, Faculty of Medicine and Health, The University of Sydney, Camperdown, NSW, Australia

Table of Contents

Overview of Methodology 2
Presence of Select Clinical Features in 20th Century Cases 3
Coding Cases for Statistical Analyses: Boxers Only 7
Frequency Tables 11
Examples of Clinical Descriptions in the Literature 13
Article Extractions 17
Martland (1928) (1) 17
Parker (1934) (2) 18
Critchley (1949) (3) 24
Raevuori-Nallinmaa (1950) (7) 28
Critchley (1957) (4) 30
Corsellis & Brierley (1959) (8) 34
Neubuerger, Sinton, & Denst (1959) (9) 36
Courville (1962) (10) 38
Spillane (1962) (11) 39
Mawdsley & Ferguson (1963) (5) 43
Payne (1968) (12) 49
Johnson (1969) (6) 52
Roberts (1969) (13) 62
Corsellis, Bruton, and Freeman-Browne (1973) (14) 71
Harvey & Newsom Davis (1974) (15) 78
Kaste, Vilkki, Sainio, Kuurne, Katevuo, & Meurala (1982) (16) 79
Ross, Cole, Thompson, & Kim (1983) (17) 80
Casson, Siegel, Sham, Campbell, Tarlau, & DiDomenico (1984) (18) 81
Roberts, Whitwell, Acland, & Bruton (1990) (19) 89
Hoff, Knabe, Bovier, & Bouras (1991) (20) 90
Hof, Bouras, Buée, Delacourte, Perl, & Morrison (1992) (21) 91
Jordan, Kanik, Horwich, Sweeney, Relkin, Petito, & Gandy (1995) (22) 92
Geddes, Vowles, Robinson, & Sutcliffe (1996) (23) 93
Williams & Tannenberg (1996) (24) 94
Jordan, Relkin, Ravdin, Jacobs, Bennett, & Gandy (1997) (25) 95
Geddes, Vowles, Nicoll, & Révész (1999) (26) 100
Newell & Drachman (1999) (27) 102
Appendix A. Cases from the 20th Century with Postmortem Findings 104
Appendix B. Reference List 105

Overview of Methodology

Purpose
The clinical features of each suspected case of Chronic Traumatic Encephalopathy (CTE) in the 20th Century are described using direct quotes from the articles. The primary focus of this review is on (i) the descriptions of mental health and psychiatric problems, and (ii) whether the cases were described as having a progressive course.

Article Review Extraction Notes
Case descriptions in the articles were often brief. Direct quotes relating to the clinical characteristics of the cases were extracted from case reports first by one author (NAH), and then a second author reviewed every article and extracted additional quotes (AKK). If cases were mentioned individually elsewhere in the articles (e.g., Discussion section), quotes were also extracted from that location. We attempted to code behavioral and psychiatric features, but this information was often not present. For depression and anxiety, we did not require a formal diagnosis. Mentioning symptoms that seemed consistent with these mental health problems was sufficient.

General Comments
The authors of the published studies in the 20th century clearly described the cases as having severe neurological and/or neuropsychiatric problems. It was striking to note how many boxers had clear neurological problems, reflecting obvious damage to the brain, while still actively fighting—and often at a young age while still in their 20s (after a long career of fighting). Many cases were described as having pronounced Parkinsonian-like signs, such as gait ataxia, masked facies, dysarthria, and tremor. Many of the cases with Parkinsonian features also had evidence of neurological lesions causing abnormalities in reflexes.

Inclusion Criterion: Boxers
Nearly every case from the 20th century was a boxer. There were two women, one who experienced prolonged interpersonal violence and one who had autism and severe head banging behavior. There was also a man with autism and severe head banging behavior described in the literature. There was also a case described as an ‘achondroplastic dwarf’ who was a circus clown with severe alcoholism and many head injuries, as well as a man with severe epilepsy who was not a boxer or a former athlete. Additionally, there were duplicated cases, which are discussed. These eight cases are included in this supplement, but they are not included in the statistical analyses or the main article.

Presence of Select Clinical Features in 20th Century Cases

Reference	Case	Case #	Boxer	Anger Control Problems	Depression	Suicidality	Anxiety	Paranoia/ Suspiciousness	Personality Change	Substance Use	Presented as Psychiatric	Course
Martland (1928)	2	1	Yes	NM	NM	NM	NM	NM	NM	0	No	Progressive
Parker (1934)	1	2	Yes	NM	NM	NM	1	NM	NM	NM	No	Nonprogressive
	2	3	Yes	NM	NM	NM	NM	NM	NM	1	No	Unable to Classify
	3	4	Yes	NM	NM	NM	NM	NM	NM	NM	No	Unable to Classify
Critchley (1949)	A	5	Yes	NM	NM	NM	NM	NM	NM	NM	No	Unable to Classify
	B	6	Yes	NM	NM	NM	NM	NM	NM	NM	No	Unable to Classify
	C	7	Yes	NM	NM	NM	1	NM	1	1	No	Unable to Classify
	D	8	Yes	1	1	NM	NM	NM	1	1	No	Unable to Classify
	E	9	Yes	1	0	NM	NM	NM	1	NM	No	Unable to Classify
	F	10	Yes	NM	NM	NM	NM	NM	NM	NM	No	Unable to Classify
	G	11	Yes	NM	NM	NM	2	NM	NM	NM	No	Unable to Classify
Raevuori-Nallinmaa (1950)	1	12	Yes	0	NM	NM	1	NM	0	NM	No	Nonprogressive
	2	13	Yes	NM	NM	NM	NM	NM	2	NM	No	Progressive
Critchley (1957)	2	14	Yes	NM	NM	NM	NM	NM	NM	NM	No	Progressive
	3	15	Yes	NM	0	NM	NM	NM	NM	NM	No	Progressive
	8	16	Yes	NM	NM	NM	NM	NM	NM	NM	No	Progressive
	9	17	Yes	NM	NM	NM	NM	NM	NM	NM	No	Progressive
	10	18	Yes	1	NM	NM	NM	NM	NM	NM	No	Progressive
	11	19	Yes	NM	NM	NM	NM	NM	1	NM	No	Progressive
	14	20	Yes	NM	NM	NM	NM	NM	NM	NM	No	Unable to Classify
Corsellis & Brierley	1	21	Yes	1	NM	NM	NM	1	1	1	No	Progressive
(1959)	2	22	Yes	1	NM	NM	NM	1	1	1	No	Progressive
Neubuerger, Sinton, & Denst (1959)	1	23	Yes	1	NM	NM	1	1	1	NM	No	Progressive
	2	24	Yes	0	NM	NM	1	NM	1	NM	No	Progressive
Courville (1962)	1	25	Yes	NM	NM	NM	NM	NM	1	1	Yes	Progressive
Spillane (1962)	1	26	Yes	NM	0	NM	NM	NM	0	1	No	Progressive
	2	27	Yes	NM	1	NM	1	NM	0	1	No	Progressive
	3	28	Yes	NM	0	NM	NM	NM	1	NM	No	Progressive
	4	29	Yes	1	NM	NM	NM	NM	NM	1	Yes	Unable to Classify
	5	30	Yes	NM	1	NM	NM	NM	NM	1	No	Progressive
Mawdsley &	1	31	Yes	1	NM	NM	NM	NM	1	1	No	Progressive
Ferguson (1963)	2	32	Yes	NM	NM	NM	NM	NM	NM	0	No	Progressive
	3	33	Yes	1	1	NM	NM	NM	1	NM	No	Progressive
	4	34	Yes	1	NM	NM	NM	1	1	0	No	Unable to Classify
	5	35	Yes	1	1	NM	NM	1	1	NM	No	Progressive
	6	36	Yes	NM	NM	NM	NM	NM	NM	NM	No	Progressive
	7	37	Yes	NM	NM	NM	NM	NM	NM	0	No	Progressive
	8	38	Yes	1	NM	NM	NM	NM	1	1	No	Unable to Classify
	9	39	Yes	NM	NM	NM	NM	NM	NM	NM	No	Progressive
	10	40	Yes	0	NM	NM	NM	NM	1	1	No	Progressive
Payne (1968)	1	41	Yes	NM	1	NM	NM	NM	1	1	Yes	Unable to Classify
	2	42	Yes	1	1	NM	NM	1	1	1	No	Progressive
	3	43	Yes	1	1	NM	NM	NM	1	1	No	Unable to Classify
	4	44	Yes	1	1	NM	NM	NM	1	1	No	Unable to Classify
	5	45	Yes	NM	NM	NM	NM	NM	NM	1	No	Unable to Classify
	6	46	Yes	NM	NM	NM	NM	NM	NM	NM	No	Unable to Classify

Reference	Case	Case #	Boxer	Anger Control Problems	Depression	Suicidality	Anxiety	Paranoia/ Suspiciousness	Personality Change	Substance Use	Presented as Psychiatric	Course
Johnson (1969)	1	47	Yes	1	NM	NM	NM	1	1	1	No	Progressive
	2	48	Yes	NM	NM	NM	NM	NM	0	0	No	Progressive
	3	49	Yes	NM	NM	NM	NM	NM	0	1	No	Nonprogressive
	5	50	Yes	NM	NM	NM	NM	NM	0	NM	No	Nonprogressive
	6	51	Yes	1	NM	NM	NM	1	1	NM	Yes	Nonprogressive
	7	52	Yes	NM	NM	NM	NM	NM	0	NM	No	Nonprogressive
	8	53	Yes	NM	1	NM	NM	1	1	NM	Yes	Progressive
	9	54	Yes	1	NM	NM	NM	1	1	1	Yes	Nonprogressive
	10	55	Yes	1	NM	NM	NM	1	1	1	Yes	Nonprogressive
	11	56	Yes	NM	NM	NM	NM	NM	NM	1	No	Progressive
	12	57	Yes	NM	NM	NM	NM	0	0	NM	No	Progressive
	13	58	Yes	NM	NM	NM	NM	NM	NM	1	No	Nonprogressive
	14	59	Yes	NM	NM	NM	NM	NM	0	NM	No	Nonprogressive
	15	60	Yes	NM	NM	NM	NM	NM	NM	NM	No	Progressive
	16	61	Yes	NM	NM	NM	NM	NM	NM	NM	No	Progressive
Roberts (1969)	1	62	Yes	NM	NM	NM	NM	1	NM	NM	Yes	Progressive
	2	63	Yes	1	NM	NM	NM	NM	1	1	No	Progressive
	3	64	Yes	NM	NM	NM	NM	NM	NM	0	No	Unable to Classify
	4	65	Yes	NM	NM	NM	NM	NM	0	1	No	Progressive
	5	66	Yes	NM	1	NM	NM	NM	1	1	No	Unable to Classify
	6	67	Yes	1	NM	NM	NM	1	1	NM	No	Progressive
	7	68	Yes	NM	NM	NM	NM	NM	NM	NM	No	Progressive
	8	69	Yes	NM	NM	NM	NM	NM	0	0	No	Nonprogressive
	9	70	Yes	NM	NM	NM	NM	NM	NM	NM	No	Unable to Classify
	10	71	Yes	NM	NM	NM	NM	NM	0	0	No	Nonprogressive
	11	72	Yes	NM	NM	NM	NM	NM	NM	1	No	Nonprogressive
Corsellis, Bruton, & Freeman-Browne (1973)	1	73	Yes	1	NM	NM	NM	NM	1	1	No	Progressive
	2	74	Yes	NM	NM	NM	NM	NM	1	0	No	Progressive
	3	75	Yes	1	NM	NM	NM	1	1	1	No	Progressive
	4	76	Yes	NM	NM	NM	NM	NM	NM	1	No	Progressive
	5	77	Yes	1	NM	NM	NM	NM	NM	1	No	Progressive
	6	78	Yes	1	NM	NM	NM	1	1	1	No	Progressive
	7	79	Yes	1	NM	NM	NM	NM	1	1	No	Progressive
	8	80	Yes	1	NM	NM	NM	NM	1	1	No	Progressive
	9	81	Yes	NM	NM	1	NM	NM	NM	1	Yes	Unable to Classify
	10	82	Yes	1	NM	NM	NM	1	1	1	No	Progressive
	11	83	Yes	1	NM	NM	NM	NM	NM	NM	No	Unable to Classify
	12	84	Yes	1	NM	NM	NM	NM	NM	NM	No	Nonprogressive
	13	85	Yes	1	NM	NM	NM	1	1	1	No	Progressive
	14	86	Yes	NM	NM	NM	NM	NM	NM	0	No	Nonprogressive
	15	87	Yes	NM	NM	NM	NM	NM	NM	0	No	Nonprogressive
Harvey & Newsom Davis (1974)	1	88	Yes	1	1	NM	NM	1	1	NM	No	Progressive
Kaste et al. (1982)	1	89	Yes	NM	NM	NM	NM	NM	2	0	No	Unable to Classify
	2	90	Yes	NM	NM	NM	NM	NM	2	0	No	Unable to Classify
	3	91	Yes	NM	NM	NM	NM	NM	NM	0	No	Unable to Classify
	4	92	Yes	NM	NM	NM	NM	NM	NM	0	No	Unable to Classify
	5	93	Yes	NM	NM	NM	NM	NM	NM	0	No	Unable to Classify
	6	94	Yes	NM	NM	NM	NM	NM	NM	0	No	Unable to Classify
	7	95	Yes	NM	NM	NM	NM	NM	NM	0	No	Unable to Classify

Reference	Case	Case #	Boxer	Anger Control Problems	Depression	Suicidality	Anxiety	Paranoia/ Suspiciousness	Personality Change	Substance Use	Presented as Psychiatric	Course
	8	96	Yes	NM	NM	NM	NM	NM	NM	0	No	Unable to Classify
	9	97	Yes	NM	NM	NM	NM	NM	NM	0	No	Unable to Classify
	10	98	Yes	NM	NM	NM	NM	NM	NM	0	No	Unable to Classify
	11	99	Yes	NM	NM	NM	NM	NM	NM	0	No	Unable to Classify
	12	100	Yes	NM	NM	NM	NM	NM	NM	0	No	Unable to Classify
	13	101	Yes	NM	NM	NM	NM	NM	NM	0	No	Unable to Classify
	14	102	Yes	NM	NM	NM	NM	NM	NM	0	No	Unable to Classify
Casson et al. (1984)	1	103	Yes	0	0	0	0	0	0	0	No	Unable to Classify
	2	104	Yes	0	0	0	0	0	0	0	No	Unable to Classify
	3	105	Yes	0	0	0	0	0	0	0	No	Unable to Classify
	4	106	Yes	0	0	0	0	0	0	0	No	Unable to Classify
	5	107	Yes	0	0	0	0	0	0	0	No	Unable to Classify
	6	108	Yes	0	0	0	0	0	0	0	No	Unable to Classify
	7	109	Yes	0	0	0	0	0	0	0	No	Unable to Classify
	8	110	Yes	0	0	0	0	0	0	0	No	Unable to Classify
	9	111	Yes	0	0	0	0	0	0	0	No	Unable to Classify
	10	112	Yes	0	0	0	0	0	0	0	No	Unable to Classify
	11	113	Yes	0	0	0	0	0	0	0	No	Unable to Classify
	12	114	Yes	0	0	0	0	0	0	0	No	Unable to Classify
	13	115	Yes	0	0	0	0	0	0	0	No	Unable to Classify
	14	116	Yes	0	0	0	0	0	0	0	No	Unable to Classify
	15	117	Yes	0	0	0	0	0	0	0	No	Unable to Classify
	16	118	Yes	0	0	0	0	0	0	0	No	Unable to Classify
	17	119	Yes	0	0	0	0	0	0	0	No	Unable to Classify
	18	120	Yes	0	0	0	0	0	0	0	No	Unable to Classify
Roberts et al. (1990)	1	121	No	NM	NM	NM	NM	NM	NM	NM	No	Unable to Classify
Hoff et al. (1991)	1	122	No	NM	NM	NM	NM	NM	NM	NM	No	Progressive
Hoff et al. (1992)	1	123	Yes	NM	1	NM	NM	NM	NM	NM	No	Unable to Classify
	2	124	Yes	NM	1	NM	NM	NM	NM	NM	No	Unable to Classify
	3	125	Yes	NM	1	NM	NM	NM	NM	NM	No	Unable to Classify
Jordan et al. (1995)	1	126	Yes	NM	NM	NM	NM	NM	NM	NM	No	Progressive
Geddes et al. (1996)	1	127	No	NM	NM	NM	NM	NM	NM	NM	No	Unable to Classify
Williams & Tannenberg (1996)	1	128	No	1	NM	NM	1	NM	NM	1	No	Unable to Classify
Jordan et al. (1997)	1	129	Yes	NM	NM	NM	NM	NM	NM	NM	No	Unable to Classify
	2	130	Yes	NM	NM	NM	NM	NM	NM	NM	No	Unable to Classify
	3	131	Yes	NM	NM	NM	NM	NM	NM	NM	No	Unable to Classify
	4	132	Yes	NM	NM	NM	NM	NM	NM	NM	No	Unable to Classify
	5	133	Yes	NM	NM	NM	NM	NM	NM	NM	No	Unable to Classify
	6	134	Yes	NM	NM	NM	NM	NM	NM	NM	No	Unable to Classify
	7	135	Yes	NM	NM	NM	NM	NM	NM	NM	No	Unable to Classify
	8	136	Yes	NM	NM	NM	NM	NM	NM	NM	No	Unable to Classify
	9	137	Yes	NM	NM	NM	NM	NM	NM	NM	No	Unable to Classify
	10	138	Yes	NM	NM	NM	NM	NM	NM	1	No	Unable to Classify
	11	139	Yes	NM	NM	NM	NM	NM	NM	1	No	Unable to Classify
	12	140	Yes	NM	NM	NM	NM	NM	NM	NM	No	Unable to Classify
	13	141	Yes	NM	NM	NM	NM	NM	NM	1	No	Unable to Classify
	14	142	Yes	NM	NM	NM	NM	NM	NM	1	No	Unable to Classify
	15	143	Yes	NM	NM	NM	NM	NM	NM	NM	No	Unable to Classify
	16	144	Yes	NM	NM	NM	NM	NM	NM	NM	No	Unable to Classify

Reference	Case	Case #	Boxer	Anger Control Problems	Depression	Suicidality	Anxiety	Paranoia/ Suspiciousness	Personality Change	Substance Use	Presented as Psychiatric	Course
	17	145	Yes	NM	NM	NM	NM	NM	NM	NM	No	Unable to Classify
	18	146	Yes	NM	NM	NM	NM	NM	NM	NM	No	Unable to Classify
	19	147	Yes	NM	NM	NM	NM	NM	NM	NM	No	Unable to Classify
	20	148	Yes	NM	NM	NM	NM	NM	NM	NM	No	Unable to Classify
	21	149	Yes	NM	NM	NM	NM	NM	NM	NM	No	Unable to Classify
	22	150	Yes	NM	NM	NM	NM	NM	NM	NM	No	Unable to Classify
	23	151	Yes	NM	NM	NM	NM	NM	NM	NM	No	Unable to Classify
	24	152	Yes	NM	NM	NM	NM	NM	NM	NM	No	Unable to Classify
	25	153	Yes	NM	NM	NM	NM	NM	1	1	No	Unable to Classify
	26	154	Yes	NM	NM	NM	NM	NM	1	1	No	Unable to Classify
	27	155	Yes	NM	NM	NM	NM	NM	NM	NM	No	Unable to Classify
	28	156	Yes	NM	NM	NM	NM	NM	NM	NM	No	Unable to Classify
	29	157	Yes	NM	1	NM	NM	NM	NM	NM	No	Unable to Classify
	30	158	Yes	NM	NM	NM	NM	NM	NM	NM	No	Unable to Classify
Geddes et al. (1999)	1	159	Yes	NM	NM	NM	NM	NM	NM	NM	No	Unable to Classify
	2	160	Yes	NM	1	NM	NM	1	1	NM	Yes	Unable to Classify
	3	161	No	NM	NM	NM	NM	NM	NM	NM	No	Unable to Classify
	4	162	No	NM	NM	NM	NM	NM	NM	NM	No	Unable to Classify
Newell & Drachman (1999)	1	163	Yes	1	NM	NM	NM	1	1	1	No	Progressive

Note: Not Present: 0; Present: 1; Unknown: 2; NM: Not Mentioned

Coding Cases for Statistical Analyses: Boxers Only

Case	Depression	Suicidality	Anxiety	Depression, Anxiety, or Suicidality	Anger Dyscontrol	Paranoia	Personality Change	Paranoia or Personality Change	Substance Misuse	Any Psychiatric or Neuropsychiatric Feature	Primarily Psychiatric	Progressive Course
1	.	.	.	.	.	.	.	.	No	.	No	Yes
2	.	.	Yes	Yes	.	.	.	.	.	Yes	No	No
3	.	.	.	.	.	.	.	.	Yes	.	No	.
4	.	.	.	.	.	.	.	.	.	.	No	.
5	.	.	.	.	.	.	.	.	.	.	No	.
6	.	.	.	.	.	.	.	.	.	.	No	.
7	.	.	Yes	Yes	.	.	Yes	Yes	Yes	Yes	No	.
8	Yes	.	.	Yes	Yes	.	Yes	Yes	Yes	Yes	No	.
9	No	.	.	No	Yes	.	Yes	Yes	.	Yes	No	.
10	.	.	.	.	.	.	.	.	.	.	No	.
11	.	.	.	.	.	.	.	.	.	.	No	.
12	.	.	Yes	Yes	No	.	No	No	.	Yes	No	No
13	.	.	.	.	.	.	.	.	.	.	No	Yes
14	.	.	.	.	.	.	.	.	.	.	No	Yes
15	No	.	.	No	.	.	.	.	.	No	No	Yes
16	.	.	.	.	.	.	.	.	.	.	No	Yes
17	.	.	.	.	.	.	.	.	.	.	No	Yes
18	.	.	.	.	Yes	.	.	.	.	Yes	No	Yes
19	.	.	.	.	.	.	Yes	Yes	.	Yes	No	Yes
20	.	.	.	.	.	.	.	.	.	.	No	.
23	.	.	Yes	Yes	Yes	Yes	Yes	Yes	.	Yes	No	Yes
24	.	.	Yes	Yes	No	.	Yes	Yes	.	Yes	No	Yes
25	.	.	.	.	.	.	Yes	Yes	Yes	Yes	Yes	Yes
26	No	.	.	No	.	.	No	No	Yes	No	No	Yes
27	Yes	.	Yes	Yes	.	.	No	No	Yes	Yes	No	Yes
28	No	.	.	No	.	.	Yes	Yes	.	Yes	No	Yes
29	.	.	.	.	Yes	.	.	.	Yes	Yes	Yes	.
30	Yes	.	.	Yes	.	.	.	.	Yes	Yes	No	Yes
31	.	.	.	.	Yes	.	Yes	Yes	Yes	Yes	No	Yes
32	.	.	.	.	.	.	.	.	No	.	No	Yes
33	Yes	.	.	Yes	Yes	.	Yes	Yes	.	Yes	No	Yes
34	.	.	.	.	Yes	Yes	Yes	Yes	No	Yes	No	.
35	Yes	.	.	Yes	Yes	Yes	Yes	Yes	.	Yes	No	Yes
36	.	.	.	.	.	.	.	.	.	.	No	Yes
37	.	.	.	.	.	.	.	.	No	.	No	Yes
38	.	.	.	.	Yes	.	Yes	Yes	Yes	Yes	No	.
39	.	.	.	.	.	.	.	.	.	.	No	Yes
40	.	.	.	.	No	.	Yes	Yes	Yes	Yes	No	Yes
41	Yes	.	.	Yes	.	.	Yes	Yes	Yes	Yes	Yes	.
42	Yes	.	.	Yes	Yes	Yes	Yes	Yes	Yes	Yes	No	Yes
43	Yes	.	.	Yes	Yes	.	Yes	Yes	Yes	Yes	No	.
44	Yes	.	.	Yes	Yes	.	Yes	Yes	Yes	Yes	No	.

Case	Depression	Suicidality	Anxiety	Depression, Anxiety, or Suicidality	Anger Dyscontrol	Paranoia	Personality Change	Paranoia or Personality Change	Substance Misuse	Any Psychiatric or Neuropsychiatric Feature	Primarily Psychiatric	Progressive Course
45	.	.	.	.	.	.	.	.	Yes	.	No	.
46	.	.	.	.	.	.	.	.	.	.	No	.
47	.	.	.	.	Yes	Yes	Yes	Yes	Yes	Yes	No	Yes
48	.	.	.	.	.	.	No	No	No	No	No	Yes
49	.	.	.	.	.	.	No	No	Yes	No	No	No
50	.	.	.	.	.	.	No	No	.	No	No	No
51	.	.	.	.	Yes	Yes	Yes	Yes	.	Yes	Yes	No
52	.	.	.	.	.	.	No	No	.	No	No	No
53	Yes	.	.	Yes	.	Yes	Yes	Yes	.	Yes	Yes	Yes
54	.	.	.	.	Yes	Yes	Yes	Yes	Yes	Yes	Yes	No
55	.	.	.	.	Yes	Yes	Yes	Yes	Yes	Yes	Yes	No
56	.	.	.	.	.	.	.	.	Yes	.	No	Yes
57	.	.	.	.	.	No	No	No	.	No	No	Yes
58	.	.	.	.	.	.	.	.	Yes	.	No	No
59	.	.	.	.	.	.	No	No	.	No	No	No
60	.	.	.	.	.	.	.	.	.	.	No	Yes
61	.	.	.	.	.	.	.	.	.	.	No	Yes
62	.	.	.	.	.	Yes	.	Yes	.	Yes	Yes	Yes
63	.	.	.	.	Yes	.	Yes	Yes	Yes	Yes	No	Yes
64	.	.	.	.	.	.	.	.	No	.	No	.
65	.	.	.	.	.	.	No	No	Yes	No	No	Yes
66	Yes	.	.	Yes	.	.	Yes	Yes	Yes	Yes	No	.
67	.	.	.	.	Yes	Yes	Yes	Yes	.	Yes	No	Yes
68	.	.	.	.	.	.	.	.	.	.	No	Yes
69	.	.	.	.	.	.	No	No	No	No	No	No
70	.	.	.	.	.	.	.	.	.	.	No	.
71	.	.	.	.	.	.	No	No	No	No	No	No
72	.	.	.	.	.	.	.	.	Yes	.	No	No
73	.	.	.	.	Yes	.	Yes	Yes	Yes	Yes	No	Yes
74	.	.	.	.	.	.	Yes	Yes	No	Yes	No	Yes
75	.	.	.	.	Yes	Yes	Yes	Yes	Yes	Yes	No	Yes
76	.	.	.	.	.	.	.	.	Yes	.	No	Yes
77	.	.	.	.	Yes	.	.	.	Yes	Yes	No	Yes
78	.	.	.	.	Yes	Yes	Yes	Yes	Yes	Yes	No	Yes
79	.	.	.	.	Yes	.	Yes	Yes	Yes	Yes	No	Yes
80	.	.	.	.	Yes	.	Yes	Yes	Yes	Yes	No	Yes
81	.	Yes	.	Yes	.	.	.	.	Yes	Yes	Yes	.
82	.	.	.	.	Yes	Yes	Yes	Yes	Yes	Yes	No	Yes
83	.	.	.	.	Yes	.	.	.	.	Yes	No	.
84	.	.	.	.	Yes	.	.	.	.	Yes	No	No
85	.	.	.	.	Yes	Yes	Yes	Yes	Yes	Yes	No	Yes
86	.	.	.	.	.	.	.	.	No	.	No	No
87	.	.	.	.	.	.	.	.	No	.	No	No
88	Yes	.	.	Yes	Yes	Yes	Yes	Yes	.	Yes	No	Yes
89	.	.	.	.	.	.	.	.	No	.	No	.

Case	Depression	Suicidality	Anxiety	Depression, Anxiety, or Suicidality	Anger Dyscontrol	Paranoia	Personality Change	Paranoia or Personality Change	Substance Misuse	Any Psychiatric or Neuropsychiatric Feature	Primarily Psychiatric	Progressive Course
90	.	.	.	.	.	.	.	.	No	.	No	.
91	.	.	.	.	.	.	.	.	No	.	No	.
92	.	.	.	.	.	.	.	.	No	.	No	.
93	.	.	.	.	.	.	.	.	No	.	No	.
94	.	.	.	.	.	.	.	.	No	.	No	.
95	.	.	.	.	.	.	.	.	No	.	No	.
96	.	.	.	.	.	.	.	.	No	.	No	.
97	.	.	.	.	.	.	.	.	No	.	No	.
98	.	.	.	.	.	.	.	.	No	.	No	.
99	.	.	.	.	.	.	.	.	No	.	No	.
100	.	.	.	.	.	.	.	.	No	.	No	.
101	.	.	.	.	.	.	.	.	No	.	No	.
102	.	.	.	.	.	.	.	.	No	.	No	.
103	No	No	No	No	No	No	No	No	No	No	No	.
104	No	No	No	No	No	No	No	No	No	No	No	.
105	No	No	No	No	No	No	No	No	No	No	No	.
106	No	No	No	No	No	No	No	No	No	No	No	.
107	No	No	No	No	No	No	No	No	No	No	No	.
108	No	No	No	No	No	No	No	No	No	No	No	.
109	No	No	No	No	No	No	No	No	No	No	No	.
110	No	No	No	No	No	No	No	No	No	No	No	.
111	No	No	No	No	No	No	No	No	No	No	No	.
112	No	No	No	No	No	No	No	No	No	No	No	.
113	No	No	No	No	No	No	No	No	No	No	No	.
114	No	No	No	No	No	No	No	No	No	No	No	.
115	No	No	No	No	No	No	No	No	No	No	No	.
116	No	No	No	No	No	No	No	No	No	No	No	.
117	No	No	No	No	No	No	No	No	No	No	No	.
118	No	No	No	No	No	No	No	No	No	No	No	.
119	No	No	No	No	No	No	No	No	No	No	No	.
120	No	No	No	No	No	No	No	No	No	No	No	.
123	Yes	.	.	Yes	.	.	.	.	.	Yes	No	.
124	Yes	.	.	Yes	.	.	.	.	.	Yes	No	.
125	Yes	.	.	Yes	.	.	.	.	.	Yes	No	.
126	.	.	.	.	.	.	.	.	.	.	No	Yes
129	.	.	.	.	.	.	.	.	.	.	No	.
130	.	.	.	.	.	.	.	.	.	.	No	.
131	.	.	.	.	.	.	.	.	.	.	No	.
132	.	.	.	.	.	.	.	.	.	.	No	.
133	.	.	.	.	.	.	.	.	.	.	No	.
134	.	.	.	.	.	.	.	.	.	.	No	.
135	.	.	.	.	.	.	.	.	.	.	No	.
136	.	.	.	.	.	.	.	.	.	.	No	.
137	.	.	.	.	.	.	.	.	.	.	No	.
138	.	.	.	.	.	.	.	.	Yes	.	No	.

Case	Depression	Suicidality	Anxiety	Depression, Anxiety, or Suicidality	Anger Dyscontrol	Paranoia	Personality Change	Paranoia or Personality Change	Substance Misuse	Any Psychiatric or Neuropsychiatric Feature	Primarily Psychiatric	Progressive Course
139	.	.	.	.	.	.	.	.	Yes	.	No	.
140	.	.	.	.	.	.	.	.	.	.	No	.
141	.	.	.	.	.	.	.	.	Yes	.	No	.
142	.	.	.	.	.	.	.	.	Yes	.	No	.
143	.	.	.	.	.	.	.	.	.	.	No	.
144	.	.	.	.	.	.	.	.	.	.	No	.
145	.	.	.	.	.	.	.	.	.	.	No	.
146	.	.	.	.	.	.	.	.	.	.	No	.
147	.	.	.	.	.	.	.	.	.	.	No	.
148	.	.	.	.	.	.	.	.	.	.	No	.
149	.	.	.	.	.	.	.	.	.	.	No	.
150	.	.	.	.	.	.	.	.	.	.	No	.
151	.	.	.	.	.	.	.	.	.	.	No	.
152	.	.	.	.	.	.	.	.	.	.	No	.
153	.	.	.	.	.	.	Yes	Yes	Yes	Yes	No	.
154	.	.	.	.	.	.	Yes	Yes	Yes	Yes	No	.
155	.	.	.	.	.	.	.	.	.	.	No	.
156	.	.	.	.	.	.	.	.	.	.	No	.
157	Yes	.	.	Yes	.	.	.	.	.	Yes	No	.
158	.	.	.	.	.	.	.	.	.	.	No	.
159	.	.	.	.	.	.	.	.	.	.	No	.
160	Yes	.	.	Yes	.	Yes	Yes	Yes	.	Yes	Yes	.
163	.	.	.	.	Yes	Yes	Yes	Yes	Yes	Yes	No	Yes
N=155	39	19	24	45	52	37	69	70	86	83	155	65

Frequency Tables

Depression

		Frequency	Percent	Valid Percent	Cumulative Percent
Valid	No	22	14.2	56.4	56.4
	Yes	17	11.0	43.6	100.0
	Total	39	25.2	100.0	
Missing	System	116	74.8		
Total		155	100.0		

Suicidality

		Frequency	Percent	Valid Percent	Cumulative Percent
Valid	No	18	11.6	94.7	94.7
	Yes	1	.6	5.3	100.0
	Total	19	12.3	100.0	
Missing	System	136	87.7		
Total		155	100.0		

Anxiety

		Frequency	Percent	Valid Percent	Cumulative Percent
Valid	No	18	11.6	75.0	75.0
	Yes	6	3.9	25.0	100.0
	Total	24	15.5	100.0	
Missing	System	131	84.5		
Total		155	100.0		

Depression, Anxiety, or Suicidality

		Frequency	Percent	Valid Percent	Cumulative Percent
Valid	No	22	14.2	48.9	48.9
	Yes	23	14.8	51.1	100.0
	Total	45	29.0	100.0	
Missing	System	110	71.0		
Total		155	100.0		

Anger Dyscontrol

		Frequency	Percent	Valid Percent	Cumulative Percent
Valid	No	21	13.5	40.4	40.4
	Yes	31	20.0	59.6	100.0
	Total	52	33.5	100.0	
Missing	System	103	66.5		
Total		155	100.0		

Paranoia

		Frequency	Percent	Valid Percent	Cumulative Percent
Valid	No	19	12.3	51.4	51.4
	Yes	18	11.6	48.6	100.0
	Total	37	23.9	100.0	
Missing	System	118	76.1		
Total		155	100.0		

Personality Change

		Frequency	Percent	Valid Percent	Cumulative Percent
Valid	No	30	19.4	43.5	43.5
	Yes	39	25.2	56.5	100.0
	Total	69	44.5	100.0	
Missing	System	86	55.5		
Total		155	100.0		

Paranoia or Personality Change

		Frequency	Percent	Valid Percent	Cumulative Percent
Valid	No	30	19.4	42.9	42.9
	Yes	40	25.8	57.1	100.0
	Total	70	45.2	100.0	
Missing	System	85	54.8		
Total		155	100.0		

Substance Misuse

		Frequency	Percent	Valid Percent	Cumulative Percent
Valid	No	43	27.7	50.0	50.0
	Yes	43	27.7	50.0	100.0
	Total	86	55.5	100.0	
Missing	System	69	44.5		
Total		155	100.0		

Any Psychiatric and Neuropsychiatric Feature

		Frequency	Percent	Valid Percent	Cumulative Percent
Valid	No	29	18.7	34.9	34.9
	Yes	54	34.8	65.1	100.0
	Total	83	53.5	100.0	
Missing	System	72	46.5		
Total		155	100.0		

Presented as Psychiatric

		Frequency	Percent	Valid Percent	Cumulative Percent
Valid	No	145	93.5	93.5	93.5
	Yes	10	6.5	6.5	100.0
	Total	155	100.0	100.0	

Progressive Course

		Frequency	Percent	Valid Percent	Cumulative Percent
Valid	No	16	10.3	24.6	24.6
	Yes	49	31.6	75.4	100.0
	Total	65	41.9	100.0	
Missing	System	90	58.1		
Total		155	100.0		

Examples of Clinical Descriptions in the Literature

Article/Quotes
**Martland (1928) (1)**<br>"Punch drunk most often affects fighters of the slugging type, who are usually poor boxers and who take considerable head punishment, seeking only to land a knockout blow. It is also common in second rate fighters used for training purposes, who may be knocked down several times a day." (p. 1103)<br><br>"The early symptoms of punch drunk usually appear in the extremities. There may be only an occasional and very slight flopping of one foot or leg in walking, noticeable only at intervals; or a slight unsteadiness in gait or uncertainty in equilibrium." (p. 1103)<br><br>"In some cases periods of slight mental confusion may occur as well as distinct slowing of muscular action… Many cases remain mild in nature and do not progress beyond this point. In others a very distinct dragging of the leg may develop and with this there is a general slowing down in muscular movements, a peculiar mental attitude characterized by hesitancy in speech, tremors of the hands and nodding movements of the head, necessitating withdrawal from the ring." (p. 1103)<br><br>"I am of the opinion that in punch drunk there is a very definite brain injury due to single or repeated blows on the head or jaw which cause multiple concussion hemorrhages in the deeper portions of the cerebrum. Such hemorrhages are very apt to occur in or near the corpora striata, in the corona radiata but almost never in the cerebral cortex or below the tentorium cerebelli." (p. 1103)<br><br>"…the fact that nearly one half of the fighters who have stayed in the game long enough develop this condition, either in a mild form or a severe and progressive form which often necessitates commitment to an asylum, warrants this report. The condition can no longer be ignored by the medical profession or the public." (p. 1107)
**Parker (1934) (2)**<br>"He walked slowly and unsteadily. His lower limbs were stiff and slow, the right being the worse of the two. At the same time, he staggered on turning suddenly and was somewhat ataxic. He held the right arm flexed when walking, and stiffly at his side, and did not swing it like the other. He hopped clumsily on the right foot, but did better with the left. The toes of both shoes were well worn down. There was a coarse, pill-rolling tremor of both hands, more marked on the right. Slight tremor of the head was present, but speech was unaltered. Speed was diminished on the right side, in both the upper and lower extremity, especially for rapid movements like drumming with fingers on a table. All tendon reflexes were greatly exaggerated, but there was no Babinski's sign on plantar stimulation. Romberg's sign was positive, and slight, horizontal nystagmus was present. Facial expression was normal and labile. Urinalysis and examination of the blood disclosed nothing abnormal, and the flocculation test for syphilis performed on the blood was negative." (p. 22)<br><br>"…the clinical picture was exceedingly complicated and did not resemble at least the more usual sequalae of epidemic encephalitis. There was, first, evident dystonia, producing grotesque muscular contractions, but the corticospinal motor system was affected also, as shown by the spasticity and Babinski phenomenon. There was also the dysarthria and the spasmodic laugh to account for, and altogether the picture was that of scattered and diffuse lesions of the brain, resembling no specific clinical syndrome." (p. 25)

Critchley (1949) (3)

"Whilst a good deal of diversity exists between the individual clinical pictures, certain grouping of symptoms commonly emerge, so as to suggest the existence of a number of clinical variants, or syndromes. The punch-drunk condition is usually a progressive one, with both psychological and neurological features, which help explain the variety of the case-records." (p. 131)

"The presenting symptom of the punch-drunk boxer is usually a headache of a dull and persistent character. Only slightly less common are the symptoms of postural dizziness; defective memory; intolerance of alcohol; diplopia; blurred vision; dysarthria; unsteadiness of gait; deafness; and epileptiform seizures. But often the history is not readily forthcoming and the lack of insight upon the patient's part may make it difficult to determine the nature, extent, and duration of the symptoms." (p. 132)

"The expression may be vacant and staring, a hallmark known to the profession by the term 'glass eye.'" (p. 132)

"More striking, however, are the psychological features. In brief, there is usually a combination of an euphoric dementia with an emotional lability. The problem is complicated by the fact that the victim is often one of subnormal intellect to start with, and it may not be easy to detect the extent or even the existence of a superadded mental deterioration. Particularly is this the case when the dullard has received an inadequate education." (p. 132)

"…help [for history taking] is derived by the story as given by relatives or friends, with its record of increasing forgetfulness, inappropriate behaviour, and lack of application…a tale of gradual falling-off in efficiency; progressive slovenliness in turn-out; and intractable delinquency. With all this goes a lack of insight; a fatuous euphoria; and an excessive mood swing. Intense irritability, sometimes with outbursts of violence, is common. Marked slowness of thought, speech and movement may be noticeable. Much more unusual is a depressive state, which may be coloured by paranoid trends and ideas of reference." (pp. 132-133)

"Neurological examination reveals a number of syndromes, comprising various combinations of such physical signs as tremors of the face, tongue and hands; sensory or cerebellar ataxia of the limbs; titubant stance; and exaggerated tendon reflexes. Nystagmus is a rare phenomenon. Blurred vision may occur with constricted visual fields but without fundus changes." (p. 133)

"Physical signs which rarely if ever occur in the punch-drunk state, include wasting; gross loss of power; and sensory impairment. Pyramidal signs are uncommon but not unknown." (p. 133)

"…various types of syndromes can often be identified:

(1) A syndrome resembling general paralysis…
(2) A syndrome resembling disseminated sclerosis
(3) A syndrome resembling brain tumour" (p. 133)

"The course of this disorder is unusual, because there is reason to believe that in some cases at least, it is progressive despite the cessation of the causative trauma. This is of great interest and important and rather suggests a slowly progressive pathological process which has been fired off by the original injuries." (p. 134)

"A few cases may be reported, to illustrate various aspect of types of the punch-drunk syndrome:

(A) Typical mild case, with headache as the outstanding complaint.
(B) Simple dementing type.
(C) Euphoric dementia and some delinquency.
(D) Gross delinquency.
(E) Resembling cerebral tumour.
(F) Resembling disseminated sclerosis.
(G) Associated with recurrent hysterical blindness." (p. 135)

Critchley (1957) (4)
"The interval between the onset of a boxing career and the onset of punch-drunk manifestation is often difficult to gauge precisely, but it is usually a matter of years." (p. 360)

"Of great interest, pathological as well as practical, is the fact that this traumatic encephalopathy is a progressive condition. Once established it not only does not permit of reversibility, but it ordinarily advances steadily. This is the case even though the boxer has retired from the ring and repeated cranial traumata are at an end." (p. 360)

"Neurologists have visualized this state of chronic traumatic encephalopathy as being based upon multiple minor cerebral contusions, possibly with initial pinpoint haemorrhages later replaced by a gliosis, cortical atrophy, and internal hydrocephalus. It is a matter of surprise that actual pathological verification should have been so dilatory in its appearance." (p. 360)

"There is much in boxing to interest a practising neurologist, and special attention should be focused upon (1) the phenomenon of groggy states as occurring during or after a contest, and (2) the condition known as traumatic progressive encephalopathy (or punch- drunkenness)." (p. 362)

Mawdsley & Ferguson (1963) (5)
"Some boxers sustain chronic neurological damage as a result of repeated head injuries in the ring. Clinically these men present a picture of dementia and impairment of motor function, with epilepsy as a less common feature. Boxers so affected are considerably disabled … The clinical signs of neurological disease are supplemented by the findings on air-encephalography of brain atrophy and of abnormalities of the septum pellucidum." (p. 800)

Johnson (1969) (6)
"The clinical features of the traumatic encephalopathy suggest that it is related to brain damage in two main areas, viz. the upper brain stem and the hippocampal-fornical system." (p. 50)

"The neurological features of the traumatic encephalopathy of boxers closely resembles this [dysarthria, ataxia, Parkinsonism and pyramidal disorder, and was due to a lesion about the level of the decussation of the superior cerebellar peduncles] and would be compatible with localized neuronal damage in the upper brain stem. This has some limited support from the three neuropathological studies of the encephalopathy which are available in the literature, all of which demonstrated gliosis in this area." (p. 50)

"There is, therefore, clinical, neuropathological and experimental evidence to support the suggestion that the neurological aspects of the encephalopathy are due to local neuronal damage in the upper brain stem, and that it probably derives from the repeated rotational torque to which the brain stem is subjected in repetitive head blows in boxing." (p. 50)

"Disturbance of recent memory functions are due in the main to lesions of the mammillothalamic-fornico-hippocampal system at three principal sites. Lesions in and around the mammillary bodies produce severe disturbances of registration and affect, as in Korsakov's psychosis; whilst in recent years the surgery of the temporal lobes has drawn attention to the severe defects of retentive memory which occur with bilateral lesions of the hippocampus. Deep-midline lesions in and around the third ventricle may produce severe defects of retention, impaired learning and decay of recent memory. The chronic amnesic syndrome present in most of these boxers was of a benign, non-progressive form and dated from the end of their fighting career. It was characterized by poor retention and defective immediate recall of impersonal data, associated with a learning defect in psychometric testing: older memories, social habits and acquired skills were not affected." (p. 50)

"Neuroradiological evidence of brain damage around the third ventricle and neurological evidence of damage in the midbrain suggest that the lesions are probably widespread throughout the amnestic system." (p. 50)

"In three cases … there was dementia due to a progressive cerebral degenerative process." (p. 50)

"Permanent impotence was present in two boxers… and temporary impotence had occurred in two others… It is considered that impotence in these cases, particularly persistent impotence, was due to lesions in the limbic system and around the third ventricle where radiological evidence of damage had been demonstrated (Johnson, 1965). It is of interest that disorders of sexual potency and disorders of recent memory may both arise from limbic lesions." (p. 51)

"Aggression must be a prominent personality trait of any successful pugilist. Three cases… had always been described as sensitive, suspicious, impulsive and aggressive, particularly when primed with alcohol, and all justified the label of "explosive psychopaths." They had all had psychiatric emergency admissions to hospitals following outbursts of uncontrollable rage. This type of "explosive diathesis" is particularly likely to occur in brain-damaged individuals after alcoholic intoxication, to which they become increasingly intolerant." (p. 51)

"A chronic psychosis was present in two cases, both of whom had a history of mental illness in a first-degree relative." (p. 51)

"Morbid jealousy was a prominent psychiatric syndrome." (p. 51)

"The neurological features may assume predominance over the psychiatric features and vice versa. They may progress independently of each other, lending further support to the view that they are dependent upon lesions in different neural systems." (p. 52)

"A great deal of polemic still surrounds the relationship of boxing to the punch-drunk syndrome. The supporters of boxing often argue that the brain damage is secondary to alcoholism. Certainly this is a feature in the case histories of many ex-boxers, but it is countered by the fact that this unique encephalopathy is never seen in chronic alcoholics who are not boxers." (p. 52)

"The neuro-psychiatric findings in 17 ex-boxers are reported, along with psychometric, EEG and AEG investigations. Sixteen had evidence of a traumatic encephalopathy, which was progressive in 8 cases and stationary in the remainder. An organic psychosyndrome was manifest in a chronic amnesic state, morbid jealousy reactions, psychosis and "explosive" personality disorder in varying combination in 14 cases. The relation between head injury and progressive cerebral disease is described." (p. 52)

Article Extractions

Martland (1928) (1)

Number of Cases Described in the Article: 1 (Case 2)
Anger Control Problems: Not Mentioned
Depression: Not Mentioned
Anxiety (Nervousness, Fear, PTSD, Obsessive-Compulsive, Worry, “Neuroses”): Not Mentioned
Suicidality: Not Mentioned
Paranoia/Suspiciousness: Not Mentioned
Personality/Demeanor Change: Not Mentioned
Substance Use: No
Cognitive Change: No
Case Presented as Psychiatric (e.g., depression and anxiety), not Neurological: No
Headaches: Not Mentioned
Type of Headaches Described: Not Applicable
Progressive Course: Yes
Non-Progressive Course: No
Unable to be Classified as Having Progressive or Non-Progressive Course: No
Delayed Onset: No
Autopsy Performed: No

Case 2 (Age: 38; Sport: Boxing)
Age started: 16 years
Age retired: 23 years
Length of career: 7 years
Age of symptom onset: 23 years
Delay between retirement and symptom onset (Age of symptom onset – Age retired): 0 years*
*No delay

- Immediately before beginning this case description, Martland describes this as a “case of advanced parkinsonian syndrome due to punch drunk.” (1106)
- “He stopped fighting in 1913, when 23 years of age, because of a **tremor in his left hand and an unsteadiness on his legs**.” (1106)
- “He had been knocked out twice; once when 22 years of age he was out for an hour. He was never sick and seldom drank.” (1106)
- “On account of symptoms of **tremor and unsteadiness**, he was often wrongly accused of being intoxicated.” (1106)
- “Since 1913 [the year he stopped fighting] **his condition has slowly progressed until he now resembles a well marked case of paralysis agitans**.” (1106)
- “His **gait is staggering and propulsive**, his **facial expression masklike**. There is marked **stammering and hesitancy in speech**. He has a **fine tremor in his hands and tongue**. The pupils are equal and react to light. The **knee kicks are slightly exaggerated**. Clonus and Babinski phenomenon are absent. The sensations are normal. The intelligence is normal. He has been under treatment in many clinics for paralysis agitans and told that his fighting did not have anything to do with his present condition.” (1106)
- “From a neurologic aspect this case is one of paralysis agitans. It either is traumatic in origin or is a primary, essential form of paralysis agitans of the juvenile type.” (1106)

Note: Martland (1928) appears to make the distinction between two forms of “punch drunk” in his conclusion: “that nearly one half of the fighters who have stayed in the game long enough develop this condition, either in a mild form or a severe and progressive form which often necessitates commitment to an asylum, warrants this report.” (1107)

Parker (1934) (2)

Number of Cases Described in the Article: 3
Cases with Anger Control Problems: Not mentioned for any cases
Cases with Depression: Not mentioned for any cases
Cases with Anxiety (Nervousness, Fear, PTSD, Obsessive-Compulsive, Worry, "Neuroses"): Case I; Not Mentioned: Cases II & III
Cases with Paranoia/Suspiciousness: Not mentioned for any cases
Cases with Suicidality: Not mentioned for any cases
Cases with Personality/Demeanor Change: Unknown: Case II; Not Mentioned: Cases I & III
Cases with Substance Use: Case II; Not Mentioned: Cases I & III
Cognitive Change: Cases I, II, & III
Cases Presented as Psychiatric (e.g., depression and anxiety), not Neurological: 0
Number of Cases with Headaches: Case II, Not Mentioned: Cases I & III
Type of Headaches Described: Parietal (Case II)
Cases Described as Having a Progressive Course: Unclear
Cases Described as Having a Non-Progressive Course: Case I
Unable to be Classified as Having Progressive or Non-Progressive Course: Cases II & III. Although Parker describes a progression of symptoms for Cases II and III, he reports that both men did not seem to deteriorate further following their visits to Mayo Clinic, over a period of 6 (Case II) and 11 years (Case III). He suggests in his summary and conclusions that some people progress while actively fighting, then become stationary or slightly improve over the years after stopping.
Delayed Onset: No delayed onset for all cases
Number of Cases with Autopsy Performed: 0

Case I (Age: 24; Sport: Boxing)
Age started: 15 years
Age retired: Likely 22 years (in 1932)
Length of career: 7 years
Age of symptom onset: Likely 22 years (in 1932)
Delay between retirement and symptom onset (Age of symptom onset – Age retired): 0 years*
*No delay

- "A man, age 24, came to The Mayo Clinic on February 9, 1934, because of **weakness of the legs** and **tremor of the hands.**" (21)
- "He had been knocked unconscious many times, but only for short intervals." (21)
- "Two months later, in June, 1932, chance of another fight had come to him, and, in spite of his **poor balance**, he thought to train for it and overcome this difficulty." (22)
- "He seemed to be fast enough on his feet in the ring at the beginning, but was unable to endure any considerable length of time, because of a **sense of fatigue of his lower extremities and a tendency of them to drag**." (22)
- "When I first saw him he still had trouble in walking, in that his **feet seemed to drag**. The right foot was worse than the left and he wore away the toes of his shoes." The **sense of balance was not normal**, but slightly better than it had been; yet he **frequently staggered**, more at times than at others." By conscious attention to walking he would pick up his feet better than he usually did, but he soon **relapsed into a dragging gait**." His mother complained that he was **more nervous since his last fight**, a little **more forgetful** of names and details, and that his hands shook. Under excitement, **he tended to have a general tremor**." (22)
- "He **walked slowly and unsteadily**. His **lower limbs were stiff and slow**, the right being the worse of the two. At the same time, he **staggered on turning suddenly** and was **somewhat ataxic**. He held the **right**

arm flexed when walking, and stiffly at his side, and did not swing it like the other. He hopped clumsily on the right foot, but did better with the left. The toes of both shoes were well worn down. There was **a coarse, pill-rolling tremor of both hands,** more marked on the right. **Slight tremor of the head** was present, but speech was unaltered. **Speed was diminished on the right side**, in both the upper and lower extremity, especially for rapid movements like drumming with fingers on a table. **All tendon reflexes were greatly exaggerated,** but there was no Babinski's sign on plantar stimulation. Romberg's sign was positive, and slight, horizontal nystagmus was present. Facial expression was normal and labile." (22)

- "There is but little doubt that the severe beating received by this pugilist in his last fight was responsible for the chain of circumstances which followed. Although he finished the fight, **the symptoms which appeared almost immediately thereafter suggested at least moderately severe injury to the brain, from which he made very far from complete recovery**…**he attributed all his difficulties to his last and futile appearance in the ring**." (22) In the opinion of Parker (1934), the patient sustained a moderate TBI that was responsible for the majority of his symptoms upon presentation. A more detailed description of this fight and subsequent symptoms is provided here:
    - "In April, 1932, the chance of making money from a bout presented itself and he re-entered the ring. He was knocked down twice in the third round, once by a blow over the right side of his jaw and the other time by a blow over the left eye and temple. He took the count of nine and then finished the fight of six round. Immediately after the fight, he felt as usual, but a few minutes later he took a drink of water, which he promptly vomited. He continued to vomit and retch, even on an empty stomach, and an hour after the fight, when attempting to go home, he reeled and staggered as if drunk. He managed to make his way home, and went to bed, but slept poorly because of nausea. Finally he slept and awakened at 10 a.m. When he attempted to arise he found himself very unsteady and was unable to balance himself. He was able to walk after a fashion, and actually had more difficulty in equilibrium than during the previous night. After consulting a physician, he returned to bed, where he stayed for three days, feeling nauseated and complaining of diplopia. He improved somewhat after this rest, and again he arose, but, while the diplopia had disappeared, he was still unsteady, although very much less so than previously. In two weeks he returned to his duty as a filling-station attendant, still slightly ataxic." (21-22)
- "…it is to be noted that **in nearly two years the condition had not become worse nor was there much improvement** beyond a certain point." (23)
- "No specific nervous syndrome appeared, such as Parkinson's disease…" (23)
- "In the first [case], the **maximal functional disability appeared immediately after a fight, improved somewhat during the next few weeks, but thereafter remained at a standstill.**" (27)
- "If probabilities are to be discussed, the first patient was without reasonable doubt injured during his last fight and his subsequent difficulties resulted from it." (28)

Case II (Age: 30; Sport: Boxing)
Age started: 16 years
Age retired: Likely 23 years (in 1921)
Length of career: 7 years
Age of symptom onset: Likely 23 years (in 1921)
Delay between retirement and symptom onset (Age of symptom onset – Age retired): 0 years*
*No delay

- A man, age 30 years, came to The Mayo Clinic on January 9, 1928, because of **difficulty in walking and in speech**." (23)
- "During this period of training he noticed that, when he was skipping rope, **his right leg tended to catch on the rope**, but he took little notice of it at the time." (23) Note: the time period being referred to here was the day after a fight during which he was knocked unconscious, then proceeded to get drunk after the fight.
- "The fourth fight was in June, 1921, and it proved to be his last. He was knocked down by the first blow of his opponent, which was a moderately hard one, and when he attempted to get up he was dizzy, had a severe headache, and promptly gave up the fight. While walking home, he found that the difficulty with his right leg had become decidedly noticeable; both lower limbs felt numb and they dragged as he walked. He decided, after this, that he was no longer fit to fight in the ring, and became a labourer in a steel mill. During the first month that elapsed after his last fight, and while he was working in the mill, the **stiffness and slowness of movement of his lower extremities** increased. This was **more marked on the right side**, for, as time went on, he wore out two soles of the right shoe to one of the left. **Walking became increasingly difficult**; he noticed that his **voice had become nasal**, and that articulate **speech was becoming more and more indistinct**. He continued at the mill about 18 months, when he was discharged because his infirmity had become obvious." (23)
- "In the latter part of 1923, **movements of the right arm became stiff, clumsy, and awkward**. **Spasmodic contractions of the muscles of the right arm, right leg and back appeared and gradually increased** so that in walking he felt a **tendency to be pulled backward**. He had **occasional parietal headaches** lasting from one to four hours, and his **powers of mental concentration, attention and memory were considerably reduced**." (23)
- "**Through the subsequent four years, his difficulties slowly and gradually increased.** He experienced **more and more difficulty in walking and in speech**, as well as **increasing spasms of the muscles of the extremities and trunk**. At the time of his examination, however, he still was able to walk alone, and he could feed and dress himself." (24)
- "…various contractions and spasms continuously at play in his muscles. In walking, his **gait was unsteady and spastic**; the **spasticity was more marked on the right** than on the left side. More conspicuous, however, were the **continuous, irregular spasms of the muscles of the right arm and back**. These were sufficiently violent to pull the man backward and to the right, with the right arm jerking and his hand slapping his thigh. These contractions were rapid, ever changing and quickly repeated." When the right arm was extended forward, **fully supinated, continuous jerking occurred**, tending to flex and pronate the arm. When, on the other hand, the arm was drawn behind the trunk in full pronation, these movements ceased. **While the patient was seated the muscles of the back were continuously jerking, pulling him backward and to the right."** The muscles of the neck and face were quiet. There was no paralysis, but all of the man's **movements were slow and clumsy**, except when he was running and jumping, which acts he performed, on the whole, better than slow walking." When he was asked to perform **rapid movements of the fingers, these movements were slow on the right side**, but **in the left leg and foot the movements were equally slow and spastic**. The **tendon reflexes on the left side were exaggerated,** and plantar stimulation on the left side produced an extensor reflex. Stroking the lips with a hard object-produced a sucking reflex (**Oppenheim's sign**). Tests of coordination, applied to the upper and lower extremities, did not disclose marked dysmetria or dyssynergia, but all tests were interfered with by the **irregular, spasmodic contractions**." There was no nystagmus. **Speech was very indistinct, slow, laboured, grating**

and nasal in tone, and the breath escaped through the nose while the man was talking, apparently because of some weakness of his soft palate." During animated conversation, he had the **tendency to break into a silly guffaw**. This, with the mouth wide open and with the tone of voice, seemed to suggest the **spasmodic laugh** seen in pseudobulbar palsy or progressive lenticular degeneration. **Mentally the man seemed childish**, and below par even when his social standing and previous education were discounted." (24)

- " [he] **had not become any worse,** and, if anything, there was a slight improvement during the six years that had elapsed since his examination at the clinic." (24)
- "All that is known is that after seven years of strenuous fighting and during the course of his career, between one bout and the next, symptoms appeared insidiously, increased in severity, and put an end to his fighting. Even after he had left the ring and had tried other pursuits, **his difficulties increased steadily up to the time he appeared at the clinic, seven years after his last fight**." (25)
- "It is, however, begging the question to assume that this patient had epidemic encephalitis, for this is incapable of proof, and **it is just as plausible and reasonable to assume that repeated injuries to his brain led to a progressive, bizarre syndrome of disease of the nervous system**." (25)
- "…the clinical picture was exceedingly complicated and did not resemble at least the more usual sequelae of epidemic encephalitis. There was first, **evident dystonia**, **producing grotesque muscular contractions**, but the corticospinal motor system was affected also, as shown by the **spasticity and Babinski phenomenon.** There was also the **dysarthria and the spasmodic laugh** to account for…" (25)
- "In the final analysis, and unsupported by necropsy, **it can only be a matter of opinion that this patient's trouble was due to his pugilism**, but in view of other cases reported a strong feeling remained that it was actually due to this." (25)
- "In the second case the **progressively crippling syndrome appeared insidiously during the last few fights of the patient's career**. His difficulties had **progressed up to the time of his examination four years later, but apparently had not become worse, and were, if anything, improved in the subsequent six years**." (28)
- "The case of the second patient must remain in doubt, for there was not the clear-cut association between his occupation and his disability." (28)

Case III (Age: 28; Sport: Boxing)
Age started: 18 years
Age retired: 28 years
Length of career: 10 years
Age of symptom onset: Likely 24 years (in 1919)
Delay between retirement and symptom onset (Age of symptom onset – Age retired): -5 years*
*Symptom onset during career

- "A man, age 28, came to The Mayo Clinic first on March 23, 1923, because of **stiffness and dragging of his legs in walking, unsteadiness of gait, and indistinct speech**." (25)
- "While serving as a mess waiter [in the Army], just before discharge in 1919, he noticed that his **left hand and foot were clumsy**; he spilled glasses of water because the left foot hit the ground awkwardly and the hand shook." (25)
- "During the latter part of 1919, while walking… his left leg hit the ground with a slapping noise, and for the first time he realized there was something wrong." (25)
- "In 1920 episodes of staggering while walking in the street appeared, and his **speech became thick**, so that he was accused unjustly a number of times of being drunk." (25)
- "Gradually, during 1921 and 1922, the **increasing stiffness of the left arm and leg** and the **unsteady gait** became more obvious. He would be slow, awkward and clumsy in the first few rounds…" (25-26)
- "He stated that he hardly ever had felt the blows he received, never had been knocked out, but had received many terrible beatings…" (26)
- "The **left arm would go into clonus**, puzzling his opponent by its unexpected and grotesque trepidation…" (26)
- "Nevertheless, **remorselessly his disease advanced, speed and precision were slower in appearing with each bout,** and a few days before the patient's first visit to the clinic his career ended in an inglorious fashion." (26)
- "When he was examined in 1923 his disability was obvious. His **speech was thick, muffled and hard to understand;** there was a rasping, laboured quality in the voice. His **gait was spastic**. The spasticity was marked on the left side, and he dragged his legs, wearing out the toe of his left shoe. There was no marked weakness. The grip of the left hand was weaker than that of the right, but the outstanding feature was the **slowness and clumsiness of the fingers, hand and arm.** In certain positions the left arm went into clonus." **Patellar clonus** was also present, and there were a few **strokes of ankle clonus** on the left. **Tendon reflexes were everywhere enormously exaggerated**, more so on the left, and on that side a Babinski phenomenon could be elicited. The jaw reflex was exaggerated, and sucking reflexes were present on stroking the lips. The man was unable to hop on the left leg, and on performing active movements, such as shadow-boxing, he was so **stiff and clumsy** that he nearly fell, and his left arm was slow and awkward. The right arm and hand seemed but little affected. There were no sensory changes, tremors or nystagmus; facial expression was mobile and pupillary reactions were normal. He seemed **below par mentally**; **his general intelligence and his memory were somewhat reduced**." (26)
- "A diagnosis was made of lateral sclerosis of the spinal cord, and some doubt was expressed as to the origin of the condition. Syphilis was suspected because of the history, and a course of antisyphilitic treatment was advised on empirical grounds. A very poor prognosis was given, since it was expected that progression of the disease could be expected." (26)
- "…he reappeared on March 7, 1934, **almost exactly eleven years after his first examination**. To our surprise we found that time had dealt gently with him. **He seemed in no way worse**, and beyond acquiring a certain amount of obesity he presented an identically similar picture to that encountered on his first examination, and was, if anything, somewhat improved, discounting the increased weight and lack of training. His disturbance of speech and gait were as conspicuous as before, but he had not deteriorated any more, physically or mentally. He had taken no antisyphilitic treatment in the meantime…" (26-27)

- "The patient, a high-grade pugilist, **developed symptoms during a period of four years, coincident with his heaviest fighting. The symptoms slowly progressed up to his final, ignominious failure in the ring, and then, for eleven years thereafter, his condition remained much the same as it was when he ceased fighting.** The picture was also different from that presented in Cases I and II; it was mainly that of injury to the corticospinal motor tracts on both sides; hence the original impression of lateral sclerosis." (27)
- "The third patient also gave evidence of **the insidious development of an affliction of the central nervous system appearing five years before he ceased to fight and progressing up to the time of his leaving the ring**. However, **after the patient's fighting career was over his condition remained stationary or perhaps improved slightly**." (28)
- "In the case of the third patient, his **difficulties appeared during his fighting career but stopped progressing as soon as he left the ring**. The probability here, that his disease was connected with his occupation, is good." (28)

Critchley (1949) (3)

Number of Cases Described in the Article: 7
Cases with Anger Control Problems: Unknown: Cases D & E; Not Mentioned: Cases A, B, C, F, & G
Cases with Depression: Case D; Not Present: Case E; Not Mentioned: Cases A, B, C, F, & G
Cases with Anxiety (Nervousness, Fear, PTSD, Obsessive-Compulsive, Worry, "Neuroses": Case C; Unknown: Case G; Not Mentioned: Cases A, B, D, E, & F
Cases with Paranoia/Suspiciousness: Not mentioned for any cases
Cases with Suicidality: Not mentioned for any cases
Cases with Personality/Demeanor Change: Case C; Unknown: Case D & E; Not Mentioned: A, B, F, & G
Cases with Substance Use: Cases C & D; Not Mentioned: A, B, E, F, & G
Cognitive Change: Case B, C, D, E, & F; Not Mentioned: A & G
Cases Presented as Psychiatric (e.g., depression and anxiety), not Neurological: 0
Cases with Headaches: Cases A, B, D, E, & F; Not Mentioned: Cases C & G
Type of Headaches Described: Intractable, persistent (Case A); early morning onset (Case F)
Cases Described as Having a Progressive Course: 0
Cases Described as Having a Non-Progressive Course: 0
Unable to be Classified as Having Progressive or Non-Progressive Course: 7 (Cases A-G). Although Critchley provides timelines in the descriptions of some of his cases, there is too little information for each case (i.e., re: progression of symptoms over time or development of new symptoms) to classify these cases as either progressive or non-progressive.
Delayed Onset: None: Case C; Not Mentioned: A, B, D, E, F, & G
Number of Cases with Autopsy Performed: 0
General Comment: Some cases were described as having extensive brain damage affecting gross motor coordination, reflexes, and speech.

Case A (Age: 31; Sport: Boxing)
Age started: 10 years
Age retired: Not mentioned
Length of career: Not mentioned
Age of symptom onset: Not mentioned
Delay between retirement and symptom onset (Age of symptom onset – Age retired): Not mentioned

- "A typical mild case of punch-drunkenness with headaches as the outstanding complaint." (135)
- "An airman, aged 31, had been complaining of **intractable and persistent headaches for some weeks, associated with postural giddiness, and nocturnal restlessness.**" (135)
- "Out of a series of over 300 bouts, each usually lasting 10-15 rounds, he had been knocked out five times." (135)
- "After some of the later contests he would feel 'groggy' and this malaise would last for days. At such times he would notice that his eyesight was temporarily impaired and he could not judge distances properly." (135) *Note: these symptoms seem to be more typical of post-concussion symptoms rather than CTE.*

Case B (Age: 21; Sport: Boxing)
Age started: 14 years
Age retired: Not mentioned
Length of career: Not mentioned
Age of symptom onset: Not mentioned
Delay between retirement and symptom onset (Age of symptom onset – Age retired): Not mentioned

- "Simple demential type of punch-drunk syndrome." (135)
- "A leading seaman, aged 21, gave a very inadequate account of **headaches for six months; ringing in the ears; postural giddiness; poor sleep; and a sense of heaviness over the top of the head**." (135)
- "On about five occasions he has been knocked out." (136)
- "He revealed **considerable retardation in speech, thought and action**. His facial appearance showed a property of expressional movement, and **he appeared dazed and bewildered**. Frequently he **failed to grasp even simple ideas**. He often forgot to finish an action which he had started; thus when told to take off his shoes and socks, he did so on one side and then had to be told to take off the other… he seemed **bemused and unaware of what was expected of him.**" (136)
- "He regarded Edward V as the reigning King of England. After a very long pause he named correctly the capital of France. Asked the capital of Germany he replied "Moscow, no that is in Russia…Berlin." (136)
- "…**no dysarthria**. The **tongue deviated to the left**. **All tendon reflexes were rather sluggish**. No other neurological abnormality could be traced." (136)

Case C (Age: 29; Sport: Boxing)
Age started: 13 years
Age retired: Still active
Length of career: Not applicable
Age of symptom onset: Not mentioned
Delay between retirement and symptom onset (Age of symptom onset – Age retired): Symptom onset during career

- "Euphoric dementia in a punch-drunk delinquent." (136)
- "Was put upon a charge in the following official terms: <<that he had been adrift 2 ½ hours; that he had lost his respirator in a pub; that when spoken to he belched offensively without regard; that he is a general nuisance; not intelligent; a bad influence; and finally that he is evil>>." (136)
- "He left school at 14 in Standard VII, with an excellent character." (136)
- "He has had about 300 fights, about three quarters of which he won; he had never been knocked out." (136)
- "For the last two years **he had been drinking rather more,** i.e., about 8 pints of beer a night. His wife said he was good-natured and cheery – perhaps unduly so; he did not mix with others very much; **of late he had been getting nervous."** (136)
- "The main objective feature in his case was a **spastic, stammering type of dysarthria,** and a habit of **talking without opening his mouth sufficiently.**" (136)

Case D (Age: 27; Sport: Boxing)
Age started: 15 years
Age retired: Not mentioned
Length of career: Not mentioned
Age of symptom onset: Not mentioned
Delay between retirement and symptom onset (Age of symptom onset – Age retired): Not mentioned

- "Criminal record in a punch-drunk boxer." (136)
- "As a boy, he had been a bright, even-tempered scholar, winning three scholarships and leaving school at 14 in Standard VII." (136)
- "His nose had been flattened and his ear contused, but he was never knocked out." (136)
- "For some weeks he had been complaining of **headaches**. His **memory began to play tricks**; he might lose himself in his own street. **At times he was depressed**." (137)
- Critchley describes a consistent pattern of desertion and impulsivity: "At the age of 22 he was charged with inflicting grievous bodily hurt, but was acquitted. Shortly after joining the Royal Navy he was adrift for 24 hours and later he deserted for 14 days. He received 90 days detention. Almost immediately afterwards he deserted for 21 days. Later he deserted a third time and was arrested for theft and sent to a civil jail for three months. He then deserted for seven weeks and married a woman he had met the day before. Immediately after the marriage ceremony he left her and had not seen her since." (137)

Case E (Age: 20; Sport: Boxing)
Age started: 15 years
Age retired: Not mentioned
Length of career: Not mentioned
Age of symptom onset: Not mentioned
Delay between retirement and symptom onset (Age of symptom onset – Age retired): Not mentioned

- "Punch-drunk syndrome resembling a cerebral tumour." (137)
- "A stoker, aged 20, described his symptoms in the following terms… 'I **can't think properly** and I **can't remember things**. My **speech has been getting bad for 18 months**… I can't talk any quicker than I am now. My **head goes blank at times**. I get **pain over the left eye nearly every morning** when I wake. If I rest it may go away, but I **feel thick-headed all day** long. **I get dizzy** but I have never lost consciousness. **My left ear is slightly deaf and my sight is not good in the left eye**. I cannot give my mind to reading, it makes my head worse; I just look at pictures.'" (137)
- "His **speech was extraordinarily slow and dysarthric**; he talked with a curious over-action of the lips. Cerebration was slow, as also his movements. He was **not unduly emotional or depressed**." (137)
- "His **memory was extremely bad for both recent and remote events**, and he could not repeat 4 digits. His knowledge of current events was very poor." (137)
- "Slight nerve type deafness in the left ear. There was **some facial and ocular apraxia**, as shown by the difficulty in getting him to screw up his eyelids, show his teeth, or follow a moving object. His **hand grips were reduced in power**. There was **no tremor, but the finger-nose test performed awkwardly**. **Tendon reflexes were sluggish**; planters flexor. The **gait was slow, lumbering and shuffling**." (137-138)
- "He **resisted with violence** any attempts at further investigation." (138)

Case F (Age: 38; Sport: Boxing)
Age started: 16 years
Age retired: Likely 34 or 35 years (“3 or 4 years ago” (138))
Length of career: Likely 18 or 19 years
Age of symptom onset: Not mentioned
Delay between retirement and symptom onset (Age of symptom onset – Age retired): Not mentioned

- “Punch-drunk syndrome reminiscent of disseminated sclerosis.” (138)
- “A stoker, aged 38… complained of **early morning headaches** (3 years); **constant diplopia to the left** (3-4 years); **reeling gait**, as if drunk; **impaired memory**; and **‘silly feelings’ in the head**.’” (138)
- “His contests usually lasted 20 rounds, and he was knocked out only once, by Len Gennons in his last contest.” (138)
- “Examination showed him to be a cheery, simple-minded fellow, **slow in cerebration**, with fair insight but with **impaired memory.** The **right pupil was greater than the left**. **On left ocular deviation, the right eye turned up and did not turn inwards fully, while the left eye gave one or two slow nystagmoid jerks. On looking right, the left eye did not move in completely. Alternating movements of the upper limbs were carried out slowly and clumsily,** especially on the left. There was **no loss of power; no tremor; no inc[o]ordination.** The **gait was rolling, and the stance unsteady.** All **tendon reflexes were exaggerated**, the knee-jerks being pendular. The **abdominals were sluggish in the left upper quadrant**. **The right plantar response was equivocal, the left one being probably extensor in type**.” (138)

Case G (Age: 25; Sport: Boxing)
Age started: 13 years (professional at 17)
Age retired: 23 years (“two years ago, he gave up the ring” (138))
Length of career: Likely 10 years
Age of symptom onset: Not mentioned
Delay between retirement and symptom onset (Age of symptom onset – Age retired): Not mentioned

- “Punch-drunk syndrome associated with recurrent hysterical blindness.” (138)
- “A stoker, aged 25, complained of **six attacks of blindness during the past four years**; the first and longest attack had lasted two months, and the shortest one only a few hours.” (138)
- “In all he had been knocked out about 60 times.” (138)
- When evaluated during a period of blindness: “His head was thrown back; his eyelids were close and fluttering; his arms were extended in front of him; he proceeded with small, shuffling step. His pupils reacted normally to light, but he said he could not discern the light of a torch. He could deviate his eyes in any direction on command, but there was a tendency towards a convergence-spasm. His optic discs were normal.” (139)
- “Later on, while still in hospital, his blindness returned, possibly as the result of receiving an anonymous letter impugning the fidelity of his wife.” (139)
- “**Neurological examination, including an E.E.G., proved negative.**” (139)

Raevuori-Nallinmaa (1950) (7)

Number of Cases Described in the Article: 2
Cases with Anger Control Problems: Not Present: Case 1 (irritability and loss of patience, but not explosiveness); Not Mentioned: Case 2
Cases with Depression: Not mentioned for either case
Cases with Suicidality: Not mentioned for either case
Cases with Anxiety (Nervousness, Fear, PTSD, Obsessive-Compulsive, Worry, "Neuroses"): Case 1; Not Mentioned: Case 2
Cases with Paranoia/Suspiciousness: Not mentioned for either case
Cases with Personality/Demeanor Change: Not Present: Case 1; Unknown: Case 2
Cases with Substance Use: Not mentioned for either case
Cognitive Change: Cases 1 & 2
Cases Presented as Psychiatric (e.g., depression and anxiety), not Neurological: 0
Cases with Headaches: Cases 1 & 2
Type of Headaches Described: Not specified
Cases Described as Having a Progressive Course: Case 2
Cases Described as Having a Non-Progressive Course: Case 1; The symptoms described in Case No. 1 appear to more closely resemble the acute and sub-acute symptoms of a moderate TBI.
Unable to be Classified as Having Progressive or Non-Progressive Course: 0
Delayed Onset: Not mentioned for either case
Number of Cases with Autopsy Performed: 0

Case No. 1 (Age: 33; Sport: Boxing)
Age started: Not mentioned ("since his youth" (53))
Age retired: Not mentioned
Length of career: 10 years
Age of symptom onset: Not mentioned
Delay between retirement and symptom onset (Age of symptom onset – Age retired): Not mentioned

- "Taken part in several matches and been knocked out 4 times." (53)
- "In recent years has become **irritable and easily falls out with others**." (53)
- "Since this event [fractured nasal bone during boxing match with no loss of consciousness] has had a **headache and felt dizziness,** because of which was taken to hospital a month afterwards. Has slept badly, the nervous system continually sensitive, the patient is **easily irritated and loses patience**. A **distinct lability of the pulse** could be verified in the hospital in several measurements, so that in different positions of the body the difference of the pulse was even 30 beats a minute. **Other neurological symptoms - including balance disturbances could not be ascertained**. The **patient himself complained of his bad memory**. **When speaking, he sometimes had difficulty in finding words, and when writing, in finding capital letter**s, especially the letter N. Objectively, too, **a slight decrease of memory could be ascertained**. **Intellectually he proved quite normal**. His behaviour was quiet and matter-of-fact. **It is true the patient himself complained of irritability, but was, however, able to control himself outwardly. During a hospital observation of 5 months there were no changes to speak of in his condition, save that the headache grew less**. After leaving the hospital he took up manual work lighter than his former job." (54)
- The former of the above cases [Case No. 1]… best corresponds to the wellknown picture of neurastheniform posttraumatic asthenia. **There had appeared during many years physical changes, which grew acutely worse after a difficult head trauma**." (55)

Case No. 2 (Age: 37; Sport: Boxing)
Age started: 17 years
Age retired: 33 (Had not boxed for four years prior to visit, at age 37)
Length of career: 16 years
Age of symptom onset: Not mentioned (in 1938)
Delay between retirement and symptom onset (Age of symptom onset – Age retired): Not mentioned

- “Has taken part in about 500 matches. Been knocked out twice.” (54)
- “In 1938 received a blow with the fist on his head, after which **the left side of the body has been slower than the right one**, and the doctor ascertained that he had had a palsy. **Has not boxed for 4 years**. **During 2 years the speech has been indistinct and by degrees grown worse**. The **throat feels stiff and sometimes he gets “water in the throat” at night**. **Thinking has become slower** than before. **Memory grown worse**. **At times headache**.” (54)
- **“Neurologically symptoms of a left side hemiplegia are verified.** When walking, the left leg stiffer than the right one. The movements of the left upper and lower limb stiffer and clumsier than those of the right one, which is seen also in the diadochokinetic movements. The muscular strength of the left upper and lower limb weakened. The left side stomach and cremaster reflexes weaker than the right side ones. The left side knee and achilles jerks more active than usual. In Romberg’s test he sways perceptibly, in Fournier’s test likewise. In position reflexes the left hand falls lower. Pain sensibility in the left upper limb, in the left half of the neck, head and face somewhat decreased. Otherwise no sensibility disturbances.” (54)
- **“About the cranial nerves it may be mentioned that the patient says he sees double pictures when looking to either side and up sidewards. The convergence reaction weakened.** The lower and partly the middle branch of the left facial nerve function more faintly than the right one. Uvula wound and withdrawn to the left. The left faucial pillar more contracted and when intoning it places itself higher. **The articulation is very indistinct and stiff, like that of an intoxicated person**. Slight “Silbenstolpern”. In the liquor no pathological changes. **A slight lability of the pulse appear**. Psychically the patient makes a primitive impression. **Clearly seems demented. Thinking and reaction slow and heavy**. **The expressions of the face slack and dull, the look heavy and somnolent.** The patient is **apathetic, unable to take the initiative, and indifferent. The speech slow and indistinct**. Is only with difficulty able to talk about his own life. **Memory is perceptibly weakened**, so that he in the ten words test is not even at the tenth time able to repeat more than 0 words, and after 5 minutes remembers 8 words. **The intellectual stage defined according to Binet-Bobertag 13 years 4 months**… The condition of the patient did not change during a hospital observation of more than a month. Because of his illness he was entirely released from military service.” (54-55)
- “In the latter case we have a typical boxer’s dementia, “punch-drunkness”. It is noteworthy that **the symptoms here had clearly increased during the last 4 years,** when the patient had no longer boxed. **Besides psychical changes, there were also neurological focal symptoms.**” (55)

Critchley (1957) (4)

Number of Cases Described in the Article: 7 (Cases 2, 3, 8-11, & 14)*
Cases with Anger Control Problems: Unknown: Case 10 (noted as losing temper with his fiancée); Not Mentioned: Cases 2, 3, 8, 9, 11, & 14
Cases with Depression: None: Case 3; Not mentioned for cases 2, 8-11, & 14
Cases with Suicidality: Not mentioned for any cases
Cases with Anxiety (Nervousness, Fear, PTSD, Obsessive-Compulsive, Worry, "Neuroses"): Not mentioned for any cases
Cases with Paranoia/Suspiciousness: Not mentioned for any cases
Cases with Personality/Demeanor Change: Case 11; Unknown: Case 3; Not Mentioned: Case 2, 8-10, & 14
Cases with Substance Use: Not mentioned for any cases
Cognitive Change: Cases 2, 3, 8, 9, 11, & 14; Not Mentioned: Case 10
Cases Presented as Psychiatric (e.g., depression and anxiety), not Neurological: 0
Cases with Headaches: Cases 2 & 14; Not Mentioned: Cases 3, 8, 9, 10, & 11
Type of Headaches Described: Persistent (Case 2)
Cases Described as Having a Progressive Course**: Cases 2, 3, 8, 9, 10, & 11
Cases Described as Having a Non-Progressive Course**: 0
Unable to be Classified as Having Progressive or Non-Progressive Course**: Case 14
Delayed Onset: No: Cases 2 & 8; Not Mentioned: 3, 9-11, & 14
Number of Cases with Autopsy Performed: 0

*Note: Victoroff (2013) identified and included 6 cases (cases 2, 3, 8-11) of what Critchley (1957) refers to as "traumatic progressive encephalopathy" from Critchley's (1957) article. This was the greatest number of cases included by any of the four major reviews. Critchley (1957) included a total of 21 cases, 11 of which he identified as examples of the "punch-drunk state" (i.e., traumatic progressive encephalopathy; cases 1-11), and 10 of which he identified as example of a "groggy state" (cases 12-21). Critchley (1957) defines this "groggy state" as the "intermediate between the knock-out (the most acute manifestation of cerebral dysfunction among boxing injuries) and the punch-drunk state (the most chronic among the disorders)" (361). Victoroff (2013) excluded the "groggy state" cases from his review, likely because they do not meet one or more of his criteria. In agreement with Victoroff (2013), we excluded all cases that Critchley (1957) identified as example of the "groggy state" except Case 14. Case 14 is like the cases described previously in this document in terms of the symptoms described. There is a notable lack of detail included in this case report regarding the nature of the boxer's injuries and his medical history. However, it is equally undescriptive compared to some other included cases from Critchley (1957) and Critchley (1957) notes that Case 14 had been diagnosed with "punch-drunkenness." Victoroff excluded Critchley's (1957) Case 4. In agreement with Victoroff (2013), we also excluded this case. Case 4 appears to be a case of epilepsy and he is even described as such by Critchley: "clinical examination was negative, but the E.E.G. was abnormal, with bilateral synchronous rhythmic slow-wave discharges suggestive of idiopathic epilepsy" (359-360).

**Critchley (1957) introduces cases 1-11 with the statement "the following clinical notes illustrate some of the characteristics of traumatic progressive encephalopathy in boxers" (359). We accepted these cases as having a progressive course even though some had insufficient details to classify them as such.

Case 2 (Age: not provided; Sport: Boxing)
Age started: Not mentioned
Age retired: Not mentioned
Length of career: Not mentioned
Age of symptom onset: Not mentioned
Delay between retirement and symptom onset (Age of symptom onset – Age retired): 0 years*
*No delay

- "Incipient Punch-drunk Disability in a First-class Heavyweight." (359)
- "**This boxer, who was always liable to headaches after each contest, had found them more persistent in the last few months.** It was noticed by others that **he was becoming a little slower, and the speech somewhat thick.** On the occasion of his last contest he was knocked out, and headaches thereafter became troublesome. Although the patient maintained that he felt well, he was **noticeably dysarthric and slow in cerebration.** There was a strong suspicion here of an early punch-drunkenness and he was advised to rest." (359)
- "…**he rapidly deteriorated both medically and professionally**, and the diagnosis of **punch-drunkenness** was ultimately agreed upon." (359)

Case 3 (Age: 61; Sport: Boxing)
Age started: Not mentioned
Age retired: Not mentioned
Length of career: 16 years (professional lightweight from 1910-1916)
Age of symptom onset: Not mentioned
Delay between retirement and symptom onset (Age of symptom onset – Age retired): Not mentioned

- "Mild Case of Punch-drunkenness with Dementia, Euphoria, and Dysarthria." (359)
- "This man, aged 61, had fought as a professional lightweight between 1910 and 1926." (359)
- "When examined he was complaining of **poor powers of concentration and forgetfulness.** He would lose the thread in his conversation and had to rely on written memoranda. **His mood was extremely euphoric and his speech grossly dysarthric**." (359)

Case 8 (Age: 27; Sport: Boxing)
Age started: 16 years
Age retired: Likely 27 years (see below bullet point 2)
Length of career: Likely 11 years
Age of symptom onset: 27 years
Delay between retirement and symptom onset (Age of symptom onset – Age retired): 0 years*
*No delay

- "Striatal Type of Punch-drunk Syndrome." (360)
- "Later he appeared in boxing booths, for it was becoming increasingly obvious to him that he was deteriorating. About this time - that is, when aged 27 years - he was rejected at the recruiting office, and **he realized then that his sight was poor and that his gait was unsteady**. **Three months later his hands became tremulous**. His symptoms increased up to a point **and then remained stationary**. On examination **he was found to be slightly forgetful**. **He had a nasal type of dysarthria, his face was expressionless, and he tended to dribble**. He had a **tremor of the outstretched hands, some increase in muscle tonus, and slight but definite ataxia**. **Tendon-jerks were increased** and there was a **right extensor response**. The **gait was titubant, short-steppage, and stiff**." (360)

Case 9 (Age: 37; Sport: Boxing)

Age started: 21 years
Age retired: 31 years
Length of career: 10 years
Age of symptom onset: Not mentioned
Delay between retirement and symptom onset (Age of symptom onset – Age retired): Not Mentioned

- "Cerebellar Type of Punch-drunkenness, reminiscent of Disseminated Sclerosis." (360)
- "This man, aged 37, was a professional boxer between the ages of 21 and 31." (360)
- "He did not think he came to much harm until a particular fight in the Albert Hall when-being distracted by a spectator calling out his name-he was knocked down. He got up after a short count and was floored a second time. This state of affairs went on again and again, and though he finished the fight he subsequently remembered very little of what happened. His symptoms consisted of **progressively increasing tremor and clumsiness of the right upper limb**. Examination revealed a **slight dysarthria, bilateral intention tremor, increased tendon-jerks, but flexor plantar responses**. Appropriate tests brought to light **a mild degree of intellectual deterioration**." (360)
    - Note: The period of time between this particular fight and the onset of his symptoms is not explicitly stated. If this was his last fight, at age 31, and he was seen at age 37, the time period would be 6 years. It would also be reasonable to assume that his symptoms upon presentation were largely due to this injury. However, it is not stated whether he continued to box after this encounter, and his symptoms were due more so to repeated brain injuries.

Case 10 (Age: 27; Sport: Boxing)
Age started: Not mentioned
Age retired: Not mentioned
Length of career: Not mentioned
Age of symptom onset: 24 years
Delay between retirement and symptom onset (Age of symptom onset – Age retired): Not mentioned

- "Striato-cerebellar Type of Punch-drunkenness." (360)
- "A professional welterweight, aged 27, whose record had gradually deteriorated, had been knocked out three times. In 1948 (aged 24) after losing his temper with his fiancée, he started to tremble all over. **This subsided except in the left hand, where the tremor persisted with increasing amplitude**. **After six months it involved the head, and his leg began to drag**. **His articulation altered**. Examination revealed a **fine tremor of the head, which was held in a curious retracted posture**. The **speech was dysarthric and staccato**. **On the left side there was an unusual type of intention tremor, associated with dysdiadochokinesis**. **The gait showed a steppage abnormality of the left leg. Both plantar responses were extensor in type.**" (360)

Case 11 (Age: 39; Sport: Boxing)
Age started: Not mentioned
Age retired: Not mentioned
Length of career: Not mentioned
Age of symptom onset: Not mentioned
Delay between retirement and symptom onset (Age of symptom onset – Age retired): Not mentioned

- “Cerebellar Type of Punch-drunkenness in an Amateur Boxer.” (360)
- “A man aged 39, who had been four times schoolboy champion of Great Britain, **began to develop tremor of the arm during the war,** on which account he was invalided from the Army. **His symptoms increased slightly and he showed an impassive, rather mask-like face; marked dysarthria; titubation of the head, with some hypertrophy of the muscles of the neck; incoordination of the arms and legs, more so on the left; with intention tremor and dysdiadochokinesis**. **Originally of superior intelligence, he later showed both an intellectual falling-off and a curious alteration in personality**.” (360)

Case 14* (Age: not provided; Sport: Boxing)
Age started: Not mentioned
Age retired: Not mentioned
Length of career: Not mentioned
Age of symptom onset: Not mentioned
Delay between retirement and symptom onset (Age of symptom onset – Age retired): Not mentioned

- “Latterly he took longer and longer to recover from each fight, **because of headaches, defective memory, and impaired speech**. His wife did not approve of his boxing and persuaded him to give it up. (**Ex-lightweight professional boxer with punch-drunkenness in which headaches and irritability were conspicuous**.)” (361)

*Note: The clinical details for this case were very limited. However, it was included in this document because the case was described as a case of punch drunk by the authors.

Corsellis & Brierley (1959) (8)

Number of Cases Described in the Article: 2
Cases with Anger Control Problems: Cases 1 & 2
Cases with Depression: Not mentioned for either case
Cases with Suicidality: Not mentioned for either case
Cases with Anxiety (Nervousness, Fear, PTSD, Obsessive-Compulsive, Worry, "Neuroses"): Not mentioned for either case
Cases with Paranoia/Suspiciousness: Cases 1 & 2
Cases with Personality/Demeanor Change: Cases 1 & 2
Cases with Substance Use: Cases 1 & 2
Cognitive Change: Cases 1 & 2
Cases Presented as Psychiatric (e.g., depression and anxiety), not Neurological: 0
Cases with Headaches: Not mentioned for either case
Type of Headaches Described: Not Applicable
Cases Described as Having a Progressive Course: Cases 1 & 2
Cases Described as Having a Non-Progressive Course: 0
Unable to be Classified as Having Progressive or Non-Progressive Course: 0
Delayed Onset: Not mentioned for either case
Number of Cases with Autopsy Performed: 2 (Cases 1 & 2)

Case 1 (Age: 56; Sport: Boxing)
Age started: Not mentioned
Age retired: Not mentioned
Length of career: Not mentioned
Age of symptom onset: Not mentioned
Delay between retirement and symptom onset (Age of symptom onset – Age retired): Not mentioned

- No clinical features, as described at the beginning of this document, are reported. Only post-mortem neuropathological findings are reported. The authors note "one was that of a man of 56 in whom a study of the brain was particularly requested because 'the patient's experience as a boxer may have contributed to his dementia.'" (719)
- "At necropsy… the only localized lesion was a small scar in the corpus callosum (presumably a tear due to past injury). A moderate degree both of cortical atrophy and of hydrocephalus was present. The essential histological findings were the same as those seen in Alzheimer's disease." (719)
- Per Corsellis, Bruton, & Freeman-Browne, 1973 article where this case was also reported:
    - "The **deterioration continued**. He became **incontinent, paranoid, and aggressive**. He ate off the table with his fingers' a cigarette packet he called a flower; he undid buttons when asked to put his tongue out. He continued to have blackouts and a few days after a convulsion he died from bronchopneumonia, aged 57 years." (285)
    - "After his discharge a few years later [after rejoining the Navy when aged 43] he **became 'more vague' and 'spoke more slowly'**. He **began to neglect his appearance** and he **complained that he could not grip things or get on a bus**. His **sight and his memory seemed a little faulty**." (285)
    - "He **wandered away bemused and had to be taken to a mental hospital. He was then 53**. On admission he was **mildly confused and complained of blackouts**. His wife, from whom he was separated, but who was still concerned for him, said that **his personality had been changing for the worse.** A brief **neurological report confirmed this and mentioned a deterioration of habits**. He **could not recall his address or the date**… **During the next year his memory deteriorated further, he was completely disorientated and needed help with dressing**. These features were emphasized in a detailed neurological investigation… He **could not undress himself**." (285)

- “…it was concluded that **‘the cortical atrophy and dementia may be related to his previous experiences as a boxer**.’” (285)
- “He had never drunk alcohol excessively but **he now became violent after two pints of beer, sometimes ‘fighting with his wife**.’” (285)
- “His wife, from whom he was separated, but who was still concerned for him, said that **his personality had been changing for the worse.** A brief **neurological report confirmed this and mentioned a deterioration of habits**.” (285)
- “He worked as a milk roundsman, a bus conductor, and later as a machine operator. When he could no longer manage this, the firm transferred him to labouring and caretaking. After two years he was given notice without warning.” (285)
- “…it was concluded that **‘the cortical atrophy and dementia may be related to his previous experiences as a boxer**.’” (285)

Case 2 (Age: 63; Sport: Boxing)
Age started: Not mentioned
Age retired: Not mentioned
Length of career: Not mentioned
Age of symptom onset: Not mentioned
Delay between retirement and symptom onset (Age of symptom onset – Age retired): Not mentioned

- “A man of 63 with a history of past championship boxing and **severe memory defect** for twenty years was admitted with a diagnosis or ‘Organic Dementia, presenile or punch-drunk.’ Again, after death four years later, the salient features were those of Alzheimer’s Disease, although in addition there was also a haemorrhage destroying the palladium on one side, with considerable hyaline thickening of the smaller vessels throughout the hemispheres.” (720)
- Per Corsellis, Bruton, & Freeman-Browne, 1973 article where this case was also reported:
  - “[His second wife] **first noticed that his memory was poor when, aged about 40**, he was serving in her shop. Later he worked as a labourer, and became unemployed for five years after the loss of an eye (unrelated to boxing). He returned to work as a road sweeper.” (283)
  - “At the **age of 60 he developed a left hemiparesis and was admitted to a mental hospital three years later**. He was **disorientated** and had **a marked loss of recent memory**. He was **paranoid and deluded, and became confused and aggressive**.” (283)
  - “He and his wife had **drinking bouts** and he was **known in his locality as a violent man**, although ‘he had been a good husband’. At the **age of 60 he developed a left hemiparesis and was admitted to a mental hospital three years later**. He was **disorientated** and had **a marked loss of recent memory**. He was **paranoid and deluded, and became confused and aggressive**.” (283)
  - “The **diagnosis was of cerebral arteriosclerosis and organic dementia, probably post-traumatic in origin**. The term **‘punch-drunk’** was used. He **gradually deteriorated over the next four years** and died aged 67.” (283)

Neubuerger, Sinton, & Denst (1959) (9)

Number of Cases Described in the Article: 2
Cases with Anger Control Problems: Case 1; Not present: Case 2
Cases with Depression: Not mentioned for either case
Cases with Suicidality: Not mentioned for either case
Cases with Anxiety (Nervousness, Fear, PTSD, Obsessive-Compulsive, Worry, "Neuroses"): Cases 1 & 2
Cases with Paranoia/Suspiciousness: Case 1; Not Mentioned: Case 2
Cases with Personality/Demeanor Change: Cases 1 & 2
Cases with Substance Use: Not mentioned for either case
Cognitive Change: Cases 1 & 2
Cases Presented as Psychiatric (e.g., depression and anxiety), not Neurological: 0
Cases with Headaches: Case 1; Not Mentioned: Case 2
Type of Headaches Described: Persistent bilateral frontal and temporal (Case 1)
Cases Described as Having a Progressive Course: Cases 1 & 2
Cases Described as Having a Non-Progressive Course: 0
Unable to be Classified as Having Progressive or Non-Progressive Course: 0
Delayed Onset: Cases 1 & 2
Number of Cases with Autopsy Performed: 1 (Case 2)

Case 1 (Age: 46; Sport: Boxing)
Age started: 14
Age retired: 36
Length of career: 22 years
Age of symptom onset: 46 years (Note: this is only when symptoms were first noted medically)
Delay between retirement and symptom onset (Age of symptom onset – Age retired): 10 years

- "It was noted incidentally that **he was tense, tremulous, suspicious, and irritable, with some delusory phenomena**. **Neurologic examination revealed only paresis of upward gaze**. **Four years later he returned because of persistent bilateral frontal and temporal headaches**. At this time a long history of boxing was elicited. He had boxed from the age of 14 to 36 and had 130 professional fights as a light heavy or heavyweight. He was knocked out 30 times. After this, he traveled with a circus as a fighter who would "take on all comers." He claimed never to have been knocked down during this time, but was frequently "out on my feet." **From the age of 50 to 53, he was observed frequently, continuing to complain of headaches, occasional blackout spells, and tremulousness**. He was unable to work consistently, but held odd jobs, such as night clerk in "skid-row" hotels. Recent examinations revealed an **intermittent fine, rapid tremor of the head and neck, which occasionally spread to the arms, particularly the right. A slight increase in tone was noted in the right arm, along with some decrease in ability to perform rapid alternating movements. Strength, tendon reflexes, coordination, and sensation were normal. There was a defect of upward gaze."** (19/403)
- "Simple tests of mental function showed inability to recall five digits forward, to subtract 7 from 100 serially, to perform any but the simplest calculations, and to recall presidents and dates of world wars. **Psychologic tests revealed loss of immediate recall and of the ability to learn new tasks. The full-scale intelligence performance was 84**." (20/404)
- "There was clinical and electroencephalographic evidence of **slowly progressive mental deterioration**." (22/406)

Case 2 (Age: 53; Sport: Boxing)
Age started: 18 years
Age retired: 24 years
Length of career: 6 years
Age of symptom onset: Likely 49 years* (in the year following his admission at age 48 for cholecystectomy, see below)
Delay between retirement and symptom onset (Age of symptom onset – Age retired): 25 years
*Note: This does not include the ‘paralyzed left side’ that he suffered in one of his last fights.

- “A 53-year-old white man was a boxer from the age of 18 to 24, having had 10 bouts before the age of 20.” (20/404)
- “The first clipping in his scrapbook states that he was knocked down three times in the first round; with **his left leg almost paralyzed and barely able to support his weight,** he continued to receive numerous blows and was knocked down again. The fight was stopped in the seventh round. **He quit the profession because of a ‘paralyzed left side,’** but tried an unsuccessful comeback one year later.” (20/404)
- “His first known admission to a hospital was at the age of 48, for a cholecystectomy. **Neurologic and personality changes were not recorded.** His wife stated that **during the following year he became forgetful, confused, irritable, and moody**. He was examined at the Mayo Clinic, where the following observations were recorded: He was an **affable, alert, restless patient, disoriented as to time and place, able to perform only the simplest calculations, and unable to find his way about Rochester unescorted.** He showed an **ataxic gait, decreased speed of motion in the left hand, increased tendon reflexes, and extensor plantar reflexes on the left**.” (20/404)
- “The impression was "**psychotic reaction**—the result of organic brain disease—most likely in the nature of a traumatic encephalopathy (punch drunk)." (20/404)
- “The patient's **condition deteriorated progressively**. Several hospitalizations were necessary because of **confusion, hyperactivity, loquaciousness, and ‘nervous breakdown.’** He died of progressive pulmonary insufficiency, having required oxygen continuously for the last few months of his life. A careful review of all the records indicated that trauma was the only factor that might have played a primary role in the cerebral disorder.” (20/404)
- “The pathological picture in the fatal case was that of a diffuse atrophy of the brain, accentuated in the frontal lobes. The gross appearance was suggestive of Pick’s disease.” (22/406)

Courville (1962) (10)

Number of Cases Described in the Article: 1
Anger Control Problems: Not Mentioned
Depression: Not Mentioned
Anxiety (Nervousness, Fear, PTSD, Obsessive-Compulsive, Worry, “Neuroses”): Not Mentioned
Suicidality: Not Mentioned
Paranoia/Suspiciousness: Not Mentioned
Personality/Demeanor Change: Yes
Substance Use: Yes
Cognitive Change: Yes
Case Presented as Psychiatric (e.g., depression and anxiety), not Neurological: Yes
Headaches: Not Mentioned
Type of Headaches Described: Not Applicable
Progressive Course: Yes
Non-Progressive Course: No
Unable to be Classified as Having Progressive or Non-Progressive Course: No
Delayed Onset: Not Mentioned
Autopsy Performed: Yes

Case 1 (Age: 49; Sport: Boxing)
Age started: Not mentioned
Age retired: Not mentioned
Length of career: 4 years or more (professional career described as relatively short)
Age of symptom onset: Not mentioned
Delay between retirement and symptom onset (Age of symptom onset – Age retired): Not mentioned

- “Development of ‘chronic brain syndrome’ characteristic of ‘punch drunk’ after a career of 4 years or more as a professional boxer. Ultimate placement in a rest home because of **erratic behavior**. **Uncertain habits regarding consumption of alcohol**. Death at age 49 from an episode of hypoglycemia, incident to treatment of diabetes mellitus. Autopsy: Cirrhosis of the liver, atrophy of pancreas with calculosis of pancreatic duct and atrophy of brain.” (161)
- “The patient was admitted to a local hospital in a **confused state**, but he was able to say that he had tripped over a rug at the rest home where he was staying (he could not remember the name of the rest home).” (161)
- “Several features in this case are of interest, although it must be recognized that it is an example of only moderately well developed case of so-called ‘punch drunk,’ or (more accurately) ‘**psychopathic deterioration** of pugilists.’ The patient’s professional career as a boxer was relatively short, which is in keeping with his only **moderately advanced intellectual deterioration**. This state may also have been modified by the presence of diabetes mellitus.” (164)

  Note: Although Courville refers to this patient as having deteriorated, there does not appear to be enough clinical description provided in the case report to definitively classify it as progressive.

Spillane (1962) (11)

Number of Cases Described in the Article: 5
Cases with Anger Control Problems: Case 4; Not Mentioned: Cases 1, 2, 3, & 5
Cases with Depression: Cases 2 & 5; None: Cases 1 & 3; Not Mentioned: Case 4
Cases with Anxiety (Nervousness, Fear, PTSD, Obsessive-Compulsive, Worry, "Neuroses"): Case 2; Not Mentioned: Cases 1 & 3- 5
Cases with Suicidality: Not mentioned for any cases
Cases with Paranoia/Suspiciousness: Not mentioned for any cases
Cases with Personality/Demeanor Change: Case 3; None: Cases 1 & 2; Not Mentioned: Cases 4 & 5
Cases with Substance Use: Cases 1, 2, 4, & 5; Not Mentioned: Case 3
Cases with Cognitive Change: Cases 2, 3, 4; None: Case 1 & 5
Cases Presented as Psychiatric (e.g., depression and anxiety), not Neurological: Case 4
Cases with Headaches: Cases 3 & 4; Not Mentioned: Case 5; None: Cases 1 & 2
Type of Headaches Described: "Thumping" (Case 4)
Cases Described as Having a Progressive Course: Cases 1, 2, 3, & 5
Cases Described as Having a Non-Progressive Course: 0
Unable to be Classified as Having Progressive or Non-Progressive Course: Case 4
Delayed Onset: Cases 2 & 3; None 1 & 5; Not Mentioned: Case 4
Number of Cases with Autopsy Performed: 1 (Case 5)

Case 1 (Age: 45; Sport: Boxing)
Age started: 13 years (professional at age 17)
Age retired: 35 years
Length of career: 18 years
Age of symptom onset: Likely 32 years (13 years ago at the time of visit, aged 45 years)
Delay between retirement and symptom onset (Age of symptom onset – Age retired): -3 years*
*Symptom onset during career

- "A former professional boxer since the age of 17, he had retired from the ring at 35 'because my friends said I was **walking unsteady and throwing a leg out**, and they said that was a sign of punch-drunk.'" (1205)
- "… he had had about 350 bouts and had only once been knocked out." (1205)
- "His **"peculiar" gait had developed in an insidious manner about 13 years ago,** and for several years **he was unaware of it** despite the comments of his wife and friends. **He was said to throw out the left leg at times, and some thought he was drunk**. He only **gradually became aware of this** unsteadiness about eight years ago. **The symptom fluctuated and was aggravated by alcohol**, but he was able to do his work and had never fallen. **If he hurries he catches his left foot in obstacles and frequently stumbles**. **Slight clumsiness of the fingers,** as when tying shoelaces or fastening buttons, **has developed in recent years**. The second symptom, again one which was noticed by others for some years before he became aware of it, was of **slurring of speech**. He has found that he has to speak slowly and carefully if he wishes to be understood. The difficulty seems to consist solely in **defective articulation; there have been no aphasic symptoms. Both the unsteadiness and slurring of speech have gradually increased over the years and seem to fluctuate**. In the past two years two further symptoms made their appearance. **There has been urgency and frequency of micturition with occasional incontinence and bouts of blurring of vision**. The latter are infrequent and sometimes consist of transient altitudinal hemianopia" (1205).
- "He had seen several ophthalmologists, who had found no abnormality. There were **no complaints of headache, vertigo, diplopia, numbness, or paraesthesiae**, and his general health was satisfactory. He complained of dyspepsia and anorexia in 1955 and was investigated in a hospital out-patient department, where it was noted that 'there is a **marked slurring or speech and unsteadiness of gait.**'" (1205)

- "The **positive neurological signs consisted of slurring dysarthria, a spastic-ataxic type of gait, and an extensor plantar reflex (left).** There was no ocular or optic abnormality; no nystagmus; optic disks, fundi, and visual fields were normal. Power in his limbs was good, although there was **some distal weakness in the left lower limb. Finger and hand movements revealed slight clumsiness**. All reflexes were brisk. There was no sensory loss. **He possessed a pleasant, frank personality, and there was nothing to suggest any deterioration.** He was rather slow, but there was **no evidence of intellectual impairment**." (1205)
- "In the past two years he has been kept under observation… **dysarthria and an unsteady gait remain the two chief disabilities. He is euphoric, and psychometric testing indicated that he possesses average intelligence (I.Q. 106) with little evidence of intellectual deterioration**." (1205)

Case 2 (Age: 52; Sport: Boxing)
Age started: Not mentioned
Age retired: 35 years
Length of career: 20 years
Age of symptom onset: Likely 40 years (12 years previously at time of visit, aged 52)
Delay between retirement and symptom onset (Age of symptom onset – Age retired): 5 years

- "He retired from the ring when he was 35 after about 300 professional fights, and at that time his only disability, he recalled, was defective vision of the right eye, the result of injury in one of his fights." (1206)
- "He said he had been knocked out on only one occasion." (1206)
- "His doctor had referred him because of **increasing weakness and lack of control of his right leg**. The patient stated that **this trouble had begun 12 years previously**. He recalled a sudden weakness of his right leg while carrying a sack of coal. At first the symptom was intermittent, but **over the years it has increased and he has found that he catches the toes of the right foot in the ground as he walks**. He stumbles, rarely falls, and there is no story of fluctuation of the complaint. During the past 12 years **there have been periods when he has experienced paraesthesiae and numbness in both his feet**. **His right arm is weak and clumsy at times. He finds difficulty in writing and holding a teacup**. **Ten years ago he began to have difficulty in urination**. There has been frequency, hesitancy, nocturia, occasional dribbling incontinence, and, on one occasion eight years ago, a bout of retention requiring catheterization. There has been no prostatic enlargement. **During the past three years his gait has been increasingly slow and unsteady, and he has often been thought to be drunk.** He is abstemious but has found that **alcohol aggravates his ataxia**. He works regularly and still feels strong. **He has no headache, vertigo, or diplopia**. He was **not aware of any difficulty with speech**, and had **no complaints to make suggesting any alteration of personality or intellectual impairment**." (1206)
- "The **positive neurological signs consisted of slow slurred speech, unsteady gait, moderate right-sided hemiparesis, and partial right optic atrophy**. There was **no dysphasia, he dragged his right foot when walking, and all movements were performed slowly and carefully**. There was **moderate ataxia on formal testing in all limbs, more marked on the right**. **There was no diplopia, ocular movements were normal**, but visual acuity on the right was 6/24 and the optic disk was very pale. Both visual fields showed moderate peripheral contraction. Visual acuity on the left was 6/12. Pupillary reflexes were sluggish on the left and practically absent on the right. **The right pupil was larger than the left.** The **remaining cranial nerves were normal.** There was **diffuse moderate weakness of the right arm and leg; the arm reflexes were normal and symmetrical. The right knee- and ankle-jerks were brisker than the left. Both plantar reflexes were flexor**. Sensory testing was not very reliable, but there was probably some impairment of superficial sensation in the distal parts of the right limbs; **vibration sensation was also impaired on the right side, but joint position sense was apparently completely absent in the right hand and foot**." (1206)
- "**His memory was unreliable**. Psychometric testing revealed **moderate intellectual impairment**." (1206)
- "He has been kept under observation during the past two years and air encephalography was recently repeated. He is still working, although under some difficulty, but **he is slower mentally**. **At times he is**

anxious or depressed. but on the whole his personality has not significantly altered. Psychometry showed that **his level of intelligence was dull normal (I.Q. 81) and that there was significant intellectual deterioration**." (1206)

Case 3 (Age: 69; Sport: Boxing)
Age started: 13 years
Age retired: 32 years
Length of career: 19 years
Age of symptom onset: Likely 59 years (it had been evident for about 10 years, at the time of visit, that his mental faculties were failing)
Delay between retirement and symptom onset (Age of symptom onset – Age retired): Likely 10 years

- "This man, aged 69, retired from the ring at 32… For about ten years it had been evident to his friends that his **mental faculties were failing**. His **memory was seriously impaired**, there were **episodes of gross amnesia and confusion and disorientation**. He was **childish at times, had little insight, and was content and euphoric**. He was **nearly always smiling and optimistic**. Short journeys often resulted in disorientation. On one occasion, while driving a car, he was involved in an accident and sustained a minor head injury, but was confused and disorientated for several days; he had complete amnesia for the accident itself." (1206)
    Note: this car accident likely resulted in a concussion with amnesia.
- "He was found to be **confused, disorientated, and unsteady**, and he complained of **headache**." (1206)
- "His speech was clear, but he was **unsteady on his feet and stumbled frequently**. His pupils were small but active; optic disks were normal. There was **slight generalized tremor of his upper limbs at times**, but power in all limbs was good. He was **ataxic on formal testing**; fell on the Romberg test; **could not stand on one leg or walk heel-toe fashion**. There was **no significant reflex or sensory abnormality. Plantar responses were flexor.** His peripheral and retinal arteries were not sclerotic. On admission **he did not know what hospital he was in, did not know the day of the week, or the year, and was generally childish and petulant in his behaviour**. **He would sulk and complain if left unattended, and smile and giggle when anyone approached his bedside**. His memories of his boxing career were reasonably retained, but places and boxers he could rarely name. He was talkative and happy." (1206-1207)
- "He now has a **progressive dementia.**" (1208)

Case 4 (Age: 33; Sport: Boxing)
Age started: 13 years (professional at age 15)
Age retired: 28 years
Length of career: 15 years
Age of symptom onset: Not mentioned
Delay between retirement and symptom onset (Age of symptom onset – Age retired): Not mentioned

- "This man, aged 33, had started boxing at the age of 13… had about 200 professional bouts and retired when he was 28. He had had about 200 professional bouts, and retired when he was 28. He said he had won 150, lost 40, and had been knocked out on five occasions." (1207)
- "**He was a wild type** but not unpleasant. He served in the Forces, enjoyed the life, had a considerable following, but resented discipline and deserted. He said he acquired and lost a fortune." (1207)
- "He had many friends and possessed considerable charm, but his fighting instincts were a nuisance." (1207)
- "He was a powerfully built man, in excellent physical condition, **without any outward abnormality**. **He admitted that his memory was failing and that he had 'thumping headaches'** when he worked in a bending condition, but he had never sought medical advice. **He felt his sense of balance was uncertain and that he was now intolerant of alcohol**. His speech was quite normal, and I could find **no abnormality on physical examination**." (1207)

- “The fourth patient, aged 33, is in excellent physical health, there is no abnormality on neurological examination, but he is **aggressive and violent**, especially when he has been drinking.” (1208)

Case 5 (Age: 45; Sport: Boxing)
Age started: 10 years (professional at age 18)
Age retired: 32 years
Length of career: 22 years
Age of symptom onset: Likely 32 years (His wife noted ill-health beginning at about this time, see below)
Delay between retirement and symptom onset (Age of symptom onset – Age retired): Likely 0 years*
*No delay

- “A laborer aged 45 entered the Department of Neurology in March 1962. He started to box when he was 10, and turned professional at 18… he retired at the age of 32.” (1207)
- “His wife thought that his ill-health began about that time. The **first symptom noted was progressive slurring of speech.** He had been **drinking heavily for a few years**, and **there were bouts of depression**. However, despite the alcoholism there were long periods of abstention, but his family and friends were puzzled because **his speech remained slurred**. Two pints of beer would often render it incomprehensible. His wife also noticed that **nocturnal urinary incontinence**, which she had formerly attributed to inebriation, persisted during his periods of sobriety. **In the past ten years he has been a patient in a mental hospital on several occasions**. During one such admission, about three years ago, it was observed that he was **dragging his left leg when he walked** and **tended to hold his left arm flexed across his body**.” (1207)
- “He was **morose and depressed**.” (1207)
- “His **speech was thick and slurred and his gait unsteady**. His pupils were normal. There was **nystagmus on right and left lateral gaze**. Power in all limbs was good, reflexes were brisk and symmetrical, and the plantar responses were flexor. There was **some diminution of sensation** (tactile, pin-prick, and vibration) **in the left leg**. B.P. 130/80. **He was slow, his power of concentration and his memory for recent events were poor, but he was not demented**. He was correctly orientated and his **behaviour was quite normal**. A speech therapist formed the view that **in addition to his gross dysarthria there was slight executive dysphasia**. **Psychometry revealed a dull normal level of intelligence (I.Q. 85), with no indication of intellectual deterioration**.” (1207)
- “Lumbar air encephalogram was normal.” (1207)
- “The fifth patient, an alcoholic, had **permanent dysarthria and ataxia.** At necropsy the only intracranial lesions consisted of small foci of softening in the cortex of the parietal, temporal, and left cerebellar lobes and the left internal capsule.” (1209)

Mawdsley & Ferguson (1963) (5)

Number of Cases Described in the Article: 10
Cases with Anger Control Problems: Cases 1, 3, 4, 5, & 8; Not Present: Case 10; Not Mentioned: Cases 2, 6, 7, & 9
Cases with Depression: Case 3 & 5; Not Mentioned: Cases 1, 2, 4, & 6-10
Cases with Suicidality: Not mentioned for any cases
Cases with Anxiety (Nervousness, Fear, PTSD, Obsessive-Compulsive, Worry, "Neuroses"): Not mentioned for any cases
Cases with Paranoia/Suspiciousness: Cases 4 & 5; Not Mentioned: Cases 1-3 & 6-10
Cases with Personality/Demeanor Change: Cases 1, 3, 4, 5, 8, & 10; Not Mentioned: Cases 2, 6, 7, & 9
Cases with Substance Use: Cases 1, 8, & 10; None: Cases 2, 4, & 7; Not Mentioned: 3, 5, 6, & 9
Cases with Cognitive Change: Cases 1-10
Cases Presented as Psychiatric (e.g., depression and anxiety), not Neurological: 0
Cases with Headaches: Cases 3 & 9; Not Mentioned: Cases 1, 2, 4-8, & 10
Type of Headaches Described: Daily, occipital, throbbing (Case 3); Constant, occipital, throbbing headaches which latterly came on at weekly intervals (Case 9)
Cases Described as Having a Progressive Course: Cases 1-3, 5-7, 9, & 10
Cases Described as Having a Non-Progressive Course: 0
Unable to be Classified as Having Progressive or Non-Progressive Course: Cases 4 & 8
Delayed Onset: Cases 3, 4, 6, & 7; None: Cases 1, 2, 5, 9, & 10; Not Mentioned: Case 8
Number of Cases with Autopsy Performed: 1 (Case 10)

Case 1 (Age: 54; Sport: Boxing)
Age started: 14 years (professional at 14)
Age retired: 32 years
Length of career: 18 years
Age of symptom onset: 30 years
Delay between retirement and symptom onset (Age of symptom onset – Age retired): -2 years*
*Symptom onset during career

- "He had 250 fights as a flyweight in a career which lasted until he was 32. He was knocked out four times... and after one bout had a period of amnesia lasting 12 hours." (795)
- "**From the age of 30** he noticed **slurring of his speech**, **difficulty in sleeping** at night, and **increasing lethargy and drowsiness during the day**. **Shortly after he had given up boxing he became unsteady in his gait** and **clumsy with his hands**, fastening laces and buttons and writing became difficult. His **speech and gait slowly deteriorated**." (795)
- "In his youth he had been aggressive, and throughout his boxing career was a heavy drinker. He is still **liable to outbursts of rage and violence but drinks little alcohol**, because small amounts aggravate his ataxia. When he was 34 he **was discharged from the Army on psychiatric grounds** and he has not worked since. Between the ages of 37 and 44 he **was admitted to mental hospitals on three occasions after episodes of violence.** His family noted a **progressive deterioration in his memory in the past 20 years**. His wife commented, too, on a **falling off in his social habits**, and on the slovenliness of his dress." (795)
- "There was clinical and psychometric evidence of **dementia with a distinct defect of retentive memory**. His **speech was slurred; his gait was ataxic, particularly when he turned.** His **pupils were small; there was restriction of upward gaze and fine nystagmus on lateral deviation of the eyes**. Muscular power was excellent, but he showed **slight intention tremor in the finger-nose test and was ataxic in the heel-knee test. Fine finger movements were impaired**. The **tendon reflexes were brisker on the left side** and the **left plantar response was equivocally extensor**." (795-796)

Case 2 (Age: 58; Sport: Boxing)
Age started: 16 years (professional at 16)
Age retired: 36 years
Length of career: 20 years
Age of symptom onset: Likely 36 years (Retired because of unsteadiness in legs, see below)
Delay between retirement and symptom onset (Age of symptom onset – Age retired): Likely 0 years*
*No delay

- "500 professional bouts between the ages of 16 and 36 in fly, bantam, and featherweight divisions; in addition, he estimated that he fought 1000 times in local booths… He was never knocked out but received many beatings, and fights were stopped because of his helpless state." (796)
- "He retired from the ring because his **legs were becoming unsteady**. People accused him of being drunk though he is a lifelong teetotaller. The **unsteadiness of his gait slowly worsened**. When he was about 38 his family and his friends commented on the **slurring of his speech**, and **2 years later he noticed tremor of his hands**. His **disability gradually increased** though he managed to work as a road sweeper until 10 years ago… He has **had frequent falls during the past 5 years and now cannot walk unaided**" (796)
- "A pleasant, garrulous man who **clinically showed a gross defect of retentive memory which was confirmed on psychometric testing**. His **speech was dysarthric and monotonous**, his **gait distinctly ataxic**. The **pupils were small**; there was **restriction of upward gaze**, and **fine nystagmus on full lateral deviation of the eyes**. His **facial expression was fixed**, and he showed a **constant, coarse tremor of the arms and hands which was more obvious on the right**. **Tone was increased in his arms and legs**. **Finger movements were clumsy**. **Plantar responses were flexor**." (796)

Case 3 (Age: 69; Sport: Boxing)
Age started: 16 years
Age retired: 24 years (after a blow which impaired vision in his left eye)
Length of career: 8 years
Age of symptom onset: 50 years
Delay between retirement and symptom onset (Age of symptom onset – Age retired): 26 years

- "A man of 69 boxed as a flyweight from the age of 16. He had about 100 fights; he lost 40, and was knocked out four times." (796)
- "At the age of 50 he and his family noticed a **deterioration in his memory**. **This defect was severe for recent events and progressed.** On occasion he forgot where to get off the bus on his way to work and would forget in mid-sentence what he wanted to say. **He became increasingly depressed and irritable**, and was apt to fall asleep during the day and to have **occipital, throbbing headaches almost daily."** (796)
- "The patient was a reserved, **depressed man** with unscarred face and hands." (796)
- "Clinical testing showed **grossly defective memory, which was confirmed by psychometry**. **Speech and gait were normal**. The left optic disc was abnormally pale. Visual acuity in the left eye was 6/60. There was **sustained nystagmus on lateral deviation of the eyes**. **Both plantar responses were equivocally extensor**." (796)

Case 4 (Age: 57; Sport: Boxing)
Age started: 15 years
Age retired: 24 years
Length of career: 9 years
Age of symptom onset: 24 years (convulsions), 52 years (paranoid hallucinations)
Delay between retirement and symptom onset (Age of symptom onset – Age retired): No delay for convulsions, 28 years (other symptoms)

- "A man of 57 fought as a flyweight 204 times between the ages of 15 and 24, also acted as a sparring partner and had over a thousand bouts in booths. He does not remember being knocked out, though several fights were stopped to save him from further punishment." (796)
- "He stopped boxing at the age of 24 because he had a **grand-mal convulsion**. For some years thereafter he had fits at intervals of a few days; their frequency gradually decreased, but he still has at least one major attack each month." (796)
- "On four occasions during the past five years, he experienced **paranoid hallucinatory states which were accompanied by clouding of consciousness and usually followed a series of grand-mal fits**. He **became violent at such times**, and **twice was admitted to a mental hospital**. These **psychotic states lasted 3 or 4 days, and recovery was spontaneous and complete on each occasion**. In the past 3 years his wife noticed **deterioration in his memory**." (796)
- "A **gross defect of retentive memory was apparent clinically, and psychometric testing showed evidence of intellectual deterioration**. The only abnormal neurological signs were **fine nystagmus on lateral deviation of the eyes and severe impairment of fine hand movements**." (796-797)

Case 5 (Age: 55; Sport: Boxing)
Age started: 15 years (professional at 15)
Age retired: 30 years (retired professionally at 21)
Length of career: 15 years
Age of symptom onset: 30 years
Delay between retirement and symptom onset (Age of symptom onset – Age retired): 0 years*
*No delay

- "A 55-year-old man fought 100 times as a lightweight professional boxer between the ages of 15 and 21. Thereafter he had many other fights in local booths and acted as a sparring partner until the age of 30. He was knocked out only once, but was beaten often." (797)
- "When he was 30 **tremor of his right hand became manifest**, particularly when he lifted a cup to his lips. The **tremor slowly increased in amplitude**, and his writing steadily deteriorated into illegibility. When he was about 35 **his gait became unsteady and his speech became slurred**. **These symptoms have progressed**. Though he was always a querulous person, quick to take offence, **his domestic life was fairly placid until about 15 years ago.** He then began to show **defects of memory and developed paranoid delusions**. He was **twice admitted to psychiatric units after outbursts of violence**." (797)
- "He was **morose and irritable**." (797)
- "There was **clinical and psychometric evidence of dementia and gross memory defect**. He was **grossly dysarthric**; **his gait was spastic and ataxic**; but his muscular development and power were exceptionally good. There **was cog-wheel rigidity of all four limbs, more obvious on the right side**. He had a constant **'pill-rolling' tremor of both hands at rest and gross intention tremor on purposive movements of the right arm**. There was **ataxia of the left arm and in the heel-shin tests**. **Both plantar responses were extensor.**" (797)

Case 6 (Age: 55; Sport: Boxing)
Age started: 12 years (professional at 16)
Age retired: 30 years
Length of career: 18 years
Age of symptom onset: 33 years
Delay between retirement and symptom onset (Age of symptom onset – Age retired): 3 years

- "A man of 55 boxed as an amateur between the ages of 12 and 16. He had 90 bouts during this time and later fought 320 times as a professional flyweight, retiring when he was 30. He was knocked out twelve times and often was badly beaten; on a dozen occasions he had long-continued amnesia after fights. At the age of 33 his **left arm and hand became weak and unsteady**. **5 years ago his wife noticed slurring of his speech and unsteadiness of his gait**. His **writing slowly deteriorated**. During the past 4 years, **on seven occasions he lost consciousness for 30 to 45 minutes**." (797)
- "The patient showed **clinical and psychometric evidence of intellectual impairment and memory defect**. His **facial expression was " fixed " and parkinsonian**, and his **speech was dysarthric**. He **had an ataxic gait**. There was **sustained nystagmus on lateral deviation of the eyes and restriction of upward gaze**. **Tone was increased in the left arm and leg**. **Fine movements of the left hand were clumsy**, and there was **gross intention tremor on purposive movements of the left arm**. There were similar, but slighter, signs on the right**.** There was **ataxia in the heel-knee test**. **Tendon reflexes were brisker on the left side**. **Both plantar responses were flexor**." (797)

Case 7 (Age: 63; Sport: Boxing)
Age started: 16 years
Age retired: 26 years
Length of career: 10 years
Age of symptom onset: 38 years
Delay between retirement and symptom onset (Age of symptom onset – Age retired): 12 years

- "A man of 63 fought as a welterweight for 10 years. He began as a boy of 16 and said he had boxed nightly until he was 26. He was knocked out once. When he was about 38 a nursing sister drew his attention to his **staggering gait**. Thereafter many people called him punch-drunk though he was conscious of little disability until his early fifties. He then noticed **unsteadiness of his gait and heaviness of his legs**, and found he was unable to run or to walk quickly. He became aware that his **speech was slurred**, though **his wife had commented on this years before**." (797)
- "His **unsteadiness of gait and his difficulty with speech slowly worsened**. He is **now unable to walk unaided**. **In the past 10 years he and his wife had noted a deterioration in his memory."** (797)
- "The patient's **speech was dysarthric**, his **gait was grossly ataxi**c, and he was **unable to walk without support**. **Upward gaze was restricted**. He had **nystagmus on full lateral deviation of the eyes, slower and coarser in amplitude on looking to the right**. There was **intention tremor of the right hand in the finger-nose test and gross ataxia in each leg when performing the heel-knee tests**. An **increase in tone of "clasp-knife" type was found in both legs**. The **tendon reflexes were exaggerated, particularly on the right side. Both plantar responses were extensor**." (797)
- "A large air-filled subarachnoid space around the cerebellum suggesting advanced cerebellar atrophy (fig. 4) was the **only abnormal finding** [Lumbar air-encephalography]." (797)

Case 8 (Age: 63; Sport: Boxing)
Age started: 14 years (professional at 14)
Age retired: 35 years
Length of career: 21 years
Age of symptom onset: Not mentioned
Delay between retirement and symptom onset (Age of symptom onset – Age retired): Not mentioned

- "A man of 59 followed his father and brothers into the ring as a professional flyweight, and fought about 600 contests between the ages of 14 and 35. He was knocked out five times, once for an hour." (797)
- "He gave up boxing because of **deterioration in his vision**. His only complaint was of poor eyesight. He denied any other disability and it was impossible to obtain a detailed history from him**. He had always been a heavy beer drinker and was involved in many brawls and had convictions for assault. Because of his violent behaviour he was admitted to a mental hospital when he was 40**. His wife left him 3 years ago because of his **drunkenness and violence**. He has not worked for three years. Whilst unemployed he lived as a tramp, begging drinks and sleeping in public lavatories, until he was admitted to a hostel 18 months ago. During his stay there he made himself a nuisance. He frequently escaped to get drunk and often assaulted his fellow-inmates." (798)
    - Note: It seems as if these aggressive outbursts may have been in part related to a personality trait and/or a result of his drinking, rather than a result of repetitive head trauma.
- "There was **gross impairment of intellect and memory**. His **facial expression was fixed**. His **speech was slow and dysarthric**. He **walked with an ataxic gait on a broad base, taking short, shuffling steps**. The optic fundi showed **macular degeneration**. Visual acuity in the right eye was 6/36. The left eye was amblyopic. The **pupils were small and reacted sluggishly to light.** There was a **defect in conjugate upward deviation and in convergence of the eyes.** The **jaw jerk was brisk**. **There was rigidity of all four limbs, and a coarse tremor of the hands. Fine movements of the fingers were impaired,** and there was **slight intention tremor in both hands**. **The tendon reflexes were brisk, particularly in the arms. Hoffman's sign was positive bilaterally. Both plantar responses were equivocally extensor. The abdominal reflexes were unobtainable**." (798)

Case 9 (Age: 33; Sport: Boxing)
Age started: 14 years
Age retired: 26 years
Length of career: 12 years
Age of symptom onset: 19 years
Delay between retirement and symptom onset (Age of symptom onset – Age retired): -7 years*
*Symptom onset during career

- "A metallurgist of 33 had a successful career as a light-heavyweight amateur boxer. He fought about 80 times between the ages of 14 and 26 and was never knocked out, though one of his fights was stopped when he was concussed and this bout was followed by a period of amnesia lasting 6 hours. When he was 19 he noticed that **his speech, previously precise and clipped, was becoming slurred**. This was apparent when he spoke quickly and when he was tired. At this time, too, **his memory deteriorated and his handwriting became untidy**. In the next two years these symptoms persisted, and he attributed his failure in examinations to the **falling off in his memory**. Fearing he was becoming punch-drunk he stopped boxing regularly, and fought only occasionally in the next 5 years. About the time he stopped boxing completely he began to have **almost constant occipital, throbbing headaches, which latterly came on at weekly intervals**. He **remained conscious of a speech difficulty**, particularly on the telephone, and **believed that his memory was faulty**. His only neurological abnormality was **slight but definite slurring dysarthria**." (798)
- "Lumbar air-encephalography showed **no abnormality**." (798)

Case 10 (Age: 51; Sport: Boxing)
Age started: 14 years
Age retired: 35 years
Length of career: 21 years
Age of symptom onset: Likely 35 years (see below)
Delay between retirement and symptom onset (Age of symptom onset – Age retired): 0 years*
*No delay

- "This patient died recently at the age of 51." (798)
- "He began his career as a boy of 14 fighting nightly in local booths. He had 300 bouts as a professional bantam weight… He was never knocked out but often lost on points later in his career." (798)
- "He had a **set, expressionless face and slurring of his speech, and was noticeably clumsy in the use of his hands**. His wife noticed a **progressive mental impairment from the age of 35**." (798)
- "**During the last 10 years of his life he spent most of the day sitting apathetically at home**. He became slovenly in his dress and habits, wearing the same clothes and underclothes for weeks on end unless he was badgered to change. He took no interest in his family's welfare. **The lethargy was in striking contrast to the liveliness and alertness described by people who knew him in his twenties.** Never a heavy drinker, he was easily rendered ataxic by small amounts of alcohol. He was an amiable man and had **no record of aggressive or violent behaviour.** He was partially impotent from the age of 35, and this was absolute after the age of 47. **On three occasions he lost consciousness for a few seconds**. He died from a squamous-cell carcinoma of the floor of his mouth." (798)
- "There was **clinical evidence of dementia**. He had **pronounced slurring dysarthria and an expressionless parkinsonian-like face**. The **tendon reflexes were abnormally brisk**. The **abdominal reflexes could not be elicited.** The **plantar responses were flexor**." (798)

Payne (1968) (12)

Number of Cases Described in the Article: 6
Cases with Anger Control Problems: Cases 2, 3 & 4; Not Mentioned: Cases 1, 5, & 6
Cases with Depression: Cases 1-4; Not Mentioned: Cases 5 & 6
Cases with Suicidality: Not mentioned for any cases
Cases with Anxiety (Nervousness, Fear, PTSD, Obsessive-Compulsive, Worry, “Neuroses”): Not mentioned for any cases
Cases with Paranoia/Suspiciousness: Case 2; Not Mentioned: Cases 1 & 3-6
Cases with Personality/Demeanor Change: Cases 1-4; Not Mentioned: 5 & 6
Cases with Substance Use: Cases 1-5; Not Mentioned: Case 6
Cases with Cognitive Change: Case 3; None: Case 4; Not Mentioned: 1, 2, 5, & 6
Cases Presented as Psychiatric (e.g., depression and anxiety), not Neurological: Case 1
Cases with Headaches: Cases 2, 3, & 4; Not Mentioned: Cases 1, 5, & 6
Type of Headaches Described: Not Specified
Cases Described as Having a Progressive Course: Case 2
Cases Described as Having a Non-Progressive Course: 0
Unable to be Classified as Having Progressive or Non-Progressive Course: Cases 1 & 3-6
Delayed Onset: None: Case 2; Not Mentioned: Cases 1 & 3-6
Number of Cases with Autopsy Performed: 6 (Autopsy performed on all cases)

Note: This paper focuses much more heavily on postmortem neuropathological findings than on clinical features. Payne states in the introduction that “this is a report of the post mortem findings in the brains of six ex-professional boxers” (173). However, there are some clinical features described for most of the cases. These are presented below.

Case 1 (Age: Not Mentioned; Sport: Boxing)
Age started: Not mentioned
Age retired: Not mentioned
Length of career: 12 years
Age of symptom onset: Not mentioned
Delay between retirement and symptom onset (Age of symptom onset – Age retired): Not mentioned

- “There is no record of the number of fights he took part in or the number of times he was knocked out.” (175)
- “He drank alcohol but the amount is unknown. In 1914 he was admitted to hospital with ‘**Manic Depressive Psychosis**’ and thereafter he spent many years in mental institutions. He had a **guilt complex** about his mis-spent youth.” (175)

Case 2 (Age: 46; Sport: Boxing)
Age started: Early teens (specific age not specified; professional at 20 years)
Age retired: 33 years
Length of career: 13 years (professional only)
Age of symptom onset: 33 years
Delay between retirement and symptom onset (Age of symptom onset – Age retired): 0 years*
*No delay

- “An ex-professional lightweight boxer of 46 years of age. He began boxing in his early teens and became a professional at about 20 years of age. It was said that he had been knocked out only once although he had taken many beatings in over 300 contests.” (176)
- “He… gave up boxing at 33 years of age; then he went to work in a factory. At about that time he **complained of headaches** and the noise of the machinery in the factory.” (176)

- "He took only an occasional drink of alcohol but he was a compulsive gambler. At 43 years of age he had left ventricular failure due to malignant hypertension. Examination at the time noted that he had **slurring of speech and unsteady gait**. His hypertension was treated but he became **depressed**, partly because of unemployment, and his **dysarthria and ataxia became more severe**. Episodes of strife occurred between his mother and himself and **he became violent and paranoid.** In the month before his death one of the attacks led him to an attempt to set his house on fire. On admission to hospital he had **dysarthria, ataxia, papilloedema and a left III nerve palsy**. He was **emotionally labile** and required constant supervision." (176)

Case 3 (Age: 46; Sport: Boxing)
Age started: 19 years
Age retired: 31 years
Length of career: 12 years
Age of symptom onset: Not specified ("after giving up boxing," see below)
Delay between retirement and symptom onset (Age of symptom onset – Age retired): Not mentioned

- "During his boxing career he had more than 200 contests and he admitted to having been knocked out on some fifty occasions. He became a heavy drinker of spirits and was treated in several hospitals for alcoholism." (177)
- After giving up boxing his main complaint was of **headaches and lack of concentration** although he also suffered with **insomnia, enuresis and periods of depression.** Eight years after the end of his boxing career it was first noted that he had **slurring of speech**. On most of his many admissions to hospital he was **pleasant and cooperative** although **on a few occasions he was abusive and threatening** to the medical staff and to other patients." (177)
- "…his **speech was slightly abnormal and there was mild impairment of coordination in his left arm.** In the last year of his life his **memory was poor**, he was **sometimes confused and vague** and he **became dirty and untidy in his dress**." (177)
- "He was found dead in bed after an alcoholic bout…" (177)

Case 4 (Age: 45; Sport: Boxing)
Age started: 19 years
Age retired: 32 years
Length of career: 14 years professional in addition to "several" years as an amateur
Age of symptom onset: Likely 40 years (see below) ("after giving up boxing," see below)
Delay between retirement and symptom onset (Age of symptom onset – Age retired): Not mentioned

- "One year later [likely age 40; one year after the ensuing seven years after retiring at age 32] he was under care as an alcoholic; **his speech was slurred and his gait shuffling**. He had been drinking about ten pints of beer a day for many years but at the time **two pints of beer made him impulsively violent**." (178)
- "His intemperate habits had resulted in marital disharmony. In the following five years he undertook repeated medical treatment for his alcoholism but in the intervals he was consuming large quantities of beer. During this period **he complained of headaches, depression, lack of concentration, insomnia and enuresis**: **his speech was persistently slurred, he had nystagmus, incoordination of his limbs and a mild sensory impairment in the left leg.** His intelligence quotient was at the lower level of normal, there was **no evidence of intellectual deterioration**." (178)
- "He died suddenly as the result of myocardial infarction." (178)

Case 5 (Age: 44; Sport: Boxing)
Age started: 15 years
Age retired: 28 years
Length of career: 13 years
Age of symptom onset: Not mentioned
Delay between retirement and symptom onset (Age of symptom onset – Age retired): Not mentioned

- "He drank alcohol, but this never appeared to present a social problem. Some of his acquaintances thought that he was quite normal while **others considered that he was 'early punchy.' His speech was occasionally indistinct and he was sometimes unsteady on his feet**. He died age 44 years from coronary atheroma and thrombosis." (181)
- "Cases 5 and 6 did not complain of any symptoms" (186)

Case 6 (Age: Not Mentioned; Sport: Boxing)
Age started: 12 years
Age retired: Not mentioned
Length of career: Professional career lasted for 4 years
Age of symptom onset: Not mentioned
Delay between retirement and symptom onset (Age of symptom onset – Age retired): Not mentioned

- "The official records show that he had 28 professional contests of which he won 17 and lost 11; he was not counted out in any of these contests although on three occasions the referee stopped the contest, once because he was unable to defend himself. His boxing career ended prematurely because of a prison sentence. He died four years later as the result of a stab wound of the heart." (181)
- "Cases 5 and 6 did not complain of any symptoms" (186)

Johnson (1969) (6)

Number of Cases Described in the Article: 15 (Cases 1-3 & 5-16)
Cases with Anger Control Problems: Cases 1, 6, 9, & 10; Not Mentioned: Cases 2, 3, 5, 7, 8, & 11-16
Cases with Depression: Case 8; Not Mentioned: Cases 1-3, 5-7, & 9-16
Cases with Suicidality: Not Mentioned for any cases
Cases with Anxiety (Nervousness, Fear, PTSD, Obsessive-Compulsive, Worry, "Neuroses"): Not Mentioned for any cases
Cases with Paranoia/Suspiciousness: Cases 1, 6, & 8-10; None: Case 12; Not Mentioned: Cases 2, 3, 5, 7, 11, & 13-16
Cases with Personality/Demeanor Change: Cases 1, 6, & 8-10; None: Cases 2, 3, 5, 7, 12, & 14; Not Mentioned: Cases 11, 13, 15, & 16
Cases with Substance Use: Cases 1, 3, 9-11, & 13; None: Case 2; Not Mentioned: Cases 5-8, 12, & 14-16
Cases with Cognitive Change: Cases 1, 2, 5-11, 13, 15, &16; None: Case 12; Unknown: Case 3; Not Mentioned: Case 14
Cases Presented as Psychiatric (e.g., depression and anxiety), not Neurological: Cases 6, 8, 9, & 10
Cases with Headaches: Not mentioned for any cases
Type of Headaches Described: Not Applicable
Cases Described as Having a Progressive Course: Cases 1, 2, 8, 11, 12, 15, & 16
Cases Described as Having a Non-Progressive Course: Cases 3, 5-7, 9, 10, 13, & 14
Unable to be Classified as Having Progressive or Non-Progressive Course: 0
Delayed Onset: None: Cases 1 & 6; Not Mentioned: Cases 2, 3, 5, & 7-16
Number of Cases with Autopsy Performed: 0

*Johnson (1969) presents 17 cases, four of which he describes in detail (Cases 1, 6, 10, and 17). However, throughout the article, Johnson provides scattered descriptions of the clinical features of different cases. Although limited, these descriptions were extracted for each case and are described below. Consistent with Victoroff (2013), Case 17 was not included because it appears that his symptoms resulted from one championship fight and a leucotomy, after which he developed epilepsy. Case 4 was also excluded because Johnson wrote, "Case 4 was not considered to show any evidence of traumatic encephalopathy" (49). Johnson states that 10 cases were already reported by Mawdsley and Ferguson (1963) and are now being assessed 4-5 years later. However, he does not state which 10 cases these are.

Case 1 (Age: 60; Sport: Boxing)
Age started: Not mentioned
Age retired: 32 years
Length of career: Not mentioned
Age of symptom onset: 32 years
Delay between retirement and symptom onset (Age of symptom onset – Age retired): 0 years*
*No delay

- "**Progression in severity of the neurological symptoms** was evident in eight cases (**1**, 2, 8, 11, 12, 15, 16, 17) and was confirmed in the four years of the follow-up… **Ataxia** combined with **festination of gait** were the most disabling features." (45-47)
- "In those with progressive neurological disorder only two (**1** and 16) had **progressive psychiatric disability.**" (47)
- "**Mental torpor**, with a **slow, sluggish response to questions** and an **absence of spontaneity in conversation**, was present in three cases (2, 8, 15), although it was a more **prominent feature** in the cases with **dementia** (**1**, 16, 17)." (47)
- "Three cases (**1**, 16, 17) suffered from **post-traumatic dementia** with **mental torpor** and **flat affective responses…**" (47)

- “A 60-year-old ex-professional flyweight developed **slurred speech**, **tremor of the hands** and **defects of recent memory at the age of 32 years**, **which forced his retirement**. He **had always been an impulsive, aggressive person** and was discharged from the Army as a ‘psychopathic personality.’ His **drinking pattern became ‘compulsive’ in type** and he was subsequently **admitted to mental hospital on four occasions for uncontrollable outbursts of rage**.” (47)
- “**Over the past 20 years** there has been a **progressive, insidious deterioration of memory and intellect**. According to his wife he mislaid objects forgot the names of familiar persons and would reiterate three or four times the same account of some recent happening. **His personal habits have deteriorated** and he has become slovenly in his dress and has had to be pressed by his family to work. **About 15 years ago he developed partial impotence** and would make **furious sexual demands on his wife**. **Morbid ideas of jealousy developed** about her and he would have **frequent outbursts of temper in his accusations**. His sexual interests were maintained, although he has been completely impotent for ten years.” (47)
- “At the age of 55 years **psychometric testing showed gross intellectual deterioration**, whilst the AEG showed ventricular dilatation, cortical atrophy and a **cavum septum pellucidum**. **Neurological and psychiatric symptoms have progressed**. He now presents severe dementia and is disabled with severe Parkinsonian symptoms, although he still lives at home with his family.” (47)
- “Diagnosis: **Dementia** due to traumatic encephalopathy.” (47)
- “In Cases **1** and 16, **dementia was paralleled by progressive neurological disability**.” (47)
- “**Persistent accusations against the wife’s supposed sexual infidelity** led to the primary **psychiatric referral** in five cases (**1**, 6, 8, 9, 10).” The **morbid jealously persisted** in all cases at follow-up, **except in Case 1**, who was **by then severely demented.”** (48)
- “Cases **1** and 6 had been **totally impotent for fifteen to twenty years**…” (48)
- “**Severe personality disorders** were present in four cases (**1**, 8, 9, 10). In three of these (**1**, 9, 10) **impulsive aggressive behavior was prominent as a life-long trait** and justified the description of ‘**explosive, psychopathic personality**.’ **Rage reaction**, **uncontrolled outbursts of anger and violence**, were prominent in the case histories of these men after their boxing careers and were attributed by them to their decreased tolerance to alcohol. All three cases had had numerous emergency **psychiatric admissions for impulsive acts of violence,** of short duration…” (48)
- “Occupational adjustments related to the extent of the **neurological and psychiatric disability**. Eight patients (**1**, 6, 9, 11, 13, 15, 16, 17) were permanently unemployed.” (48)
- In three cases (**1**, 16, 17) there was **dementia due to a progressive cerebral degenerative process**.” (50)
- “The patient’s **accusations and suspicions of the wife’s infidelity occurred episodically** and were seen as **abnormal psychogenic reactions** in sensitive personalities. It is difficult not to presume that impotence in these cases had played some part as a pathoplastic feature in determining this particular reaction” (51-52).
- “Aggression must be a prominent personality characteristic of any successful pugilist. Three cases (**1**, 9, 10) had always been described as **sensitive, suspicious, impulsive and aggressive**, particularly when primed with alcohol, and all justified the label of “explosive psychopaths.” (51)
- “It is noteworthy in this series that only four were heavy social drinkers (Cases 9, 10, 11, 13) and only two (**1** and 3) had been compulsive drinkers in early adult life.” (52)

Case 2 (Age: Not mentioned: Sport: Boxing)
Age started: Not mentioned
Age retired: Not Mentioned
Length of career: Not mentioned
Age of symptom onset: Not Mentioned
Delay between retirement and symptom onset (Age of symptom onset – Age retired): Not Mentioned

- "**Progression in severity of the neurological symptoms** was evident in eight cases (1, **2**, 8, 11, 12, 15, 16, 17) and was confirmed in the four years of the follow-up… **Ataxia** combined with **festination of gait** were the most disabling features." (45-47)
- "A **defect of memory** affecting the **retention and immediate recall of recent events** was present in 11 cases (**2**, 3, 5, 6, 7, 8, 9, 10, 11, 13, 15) and has usually been present since the end of the boxer's career." (47)
- "**Mental torpor**, with a **slow, sluggish response to questions** and an **absence of spontaneity in conversation**, was present in three cases (**2**, 8, 15)" (47).
- "Seven cases (**2**, 3, 4, 5, 7, 12, 14) **remained well adjusted** in marriage and occupation and showed **no personality deviation**." (48)
- "Case 2 showed a **severe neurological, progressive disease**, but had a **normal air encephalogram** and **no evidence of brain damage on psychometric testing**. It is presumed that the responsible lesion in Case 2 was limited to the brain stem and did not interfere with higher cerebral function." (49)
- "One of the most seriously affected boxers (Case **2**) was a life-long teetotaller." (52)

Case 3 (Age: Not mentioned: Sport: Boxing)
Age started: Not mentioned
Age retired: Not Mentioned
Length of career: Not mentioned
Age of symptom onset: Not Mentioned
Delay between retirement and symptom onset (Age of symptom onset – Age retired): Not Mentioned

- "…there was **no evidence of progression of the neurological syndrome** in eight cases (**3**, 5, 6, 7, 9, 10, 13, 14)." (47)
- "A **defect of memory** affecting the **retention and immediate recall of recent events** was present in 11 cases (2, **3**, 5, 6, 7, 8, 9, 10, 11, 13, 15) and has usually been present since the end of the boxer's career." (47)
- "In Case 3 a **severe registration and retention defect** was present and resembled that seen in a Korsakov state, although other features of this syndrome were absent. The patient had been a heavy drinker early in his career and since **little evidence of the traumatic encephalopathy was present** it was felt that this retention defect was largely **attributable to alcohol**." (47)
- "Seven cases (2, **3**, 4, 5, 7, 12, 14) **remained well adjusted** in marriage and occupation and showed **no personality deviation**." (48)
- "It is noteworthy in this series that only four were heavy social drinkers (Cases 9, 10, 11, 13) and only two (1 and **3**) had been compulsive drinkers in early adult life… In only once case (Case **3**) could alcohol be held to be the major aetiological factor in the organic psychosyndrome." (52)
- "Cases **3**, 5, 6, 7, 9, 10, 13, 14 **had not deteriorated over the years of the follow-up of this study**, and indeed according to relatives' accounts they had **deteriorated little since the end of their fighting careers**." (52)

Case 5 (Age: Not mentioned: Sport: Boxing)
Age started: Not mentioned
Age retired: Not Mentioned
Length of career: Not mentioned
Age of symptom onset: Not Mentioned
Delay between retirement and symptom onset (Age of symptom onset – Age retired): Not Mentioned

- "…there was **no evidence of progression of the neurological syndrome** in eight cases (3, **5**, 6, 7, 9, 10, 13, 14)." (47)
- "A **defect of memory** affecting the **retention and immediate recall of recent events** was present in 11 cases (2, 3, **5**, 6, 7, 8, 9, 10, 11, 13, 15) and has usually been present since the end of the boxer's career." (47)
- "Seven cases (2, 3, 4, **5,** 7, 12, 14) **remained well adjusted** in marriage and occupation and showed **no personality deviation**." (48)
- "Cases 3, **5**, 6, 7, 9, 10, 13, 14 **had not deteriorated over the years of the follow-up of this study**, and indeed according to relatives' accounts they had **deteriorated little since the end of their fighting careers**." (52)

Case 6 (Age: 60; Sport: Boxing)
Age started: Not mentioned
Age retired: 30 years
Length of career: Not mentioned
Age of symptom onset: 30 years
Delay between retirement and symptom onset (Age of symptom onset – Age retired): 0 years*
*No delay

- "…there was **no evidence of progression of the neurological syndrome** in eight cases (3, 5, **6**, 7, 9, 10, 13, 14)." (47)
- "A **defect of memory** affecting the **retention and immediate recall of recent events** was present in 11 cases (2, 3, 5, **6**, 7, 8, 9, 10, 11, 13, 15) and has usually been present since the end of the boxer's career." (47)
- "**Persistent accusations against the wife's supposed sexual infidelity** led to the primary **psychiatric referral** in five cases (1, **6**, 8, 9, 10)." The **morbid jealously persisted** in all cases at follow-up, except in Case 1, who was by then severely demented." (48)
- "A 60-year-old light-weight, ex-professional boxer **retired at 30 years from the ring when he developed severe intention tremor of the righthand**, **ataxic gait** and **poor memory for recent events**. The condition **progressed for five years** but **has since been relatively stationary**. At **45 years he became impotent**, although making excessive sexual demands upon his wife. **He then began to accuse her of being pregnant by his son, brother, nephew and later his neighbours**. He became **increasingly querulous and argumentative**, and was **finally admitted to a local psychiatric unit where he was diagnosed as 'paranoia due to brain injury**.' He was noted to have a **marked impairment in retentive memory**: there were no first rank symptoms of schizophrenia. Four E.C.T.s made him more irritable, and the treatment was abandoned and he returned home. Over the past fifteen years his family have tolerated his **incessant suspicion and accusations about his wife's supposed infidelity with neighbours**. Owing to his **quarrelsome attitudes** he has not worked for ten years. **Apart from some recent further deterioration in his memory, the neurological and psychiatric syndromes have changed little over the past fifteen years**." (48)
- "Diagnosis: Chronic symptomatic **paranoid psychosis**." (48)
- "Cases 1 and **6** had been **totally impotent for fifteen to twenty years**" (48).

- "Occupational adjustments related to the extent of the **neurological and psychiatric disability**. Eight patients (1, **6**, 9, 11, 13, 15, 16, 17) were permanently unemployed." (48)
- "A **persistent psychosis** was present…Case 6… had a **chronic paranoid psychosis** which a **chronic amnesic state** due to underlying brain damage." (48-49)
- "Case 6 was a chronic organic psychosyndrome due to brain damage, with a florid delusional system relating to impotence and morbid jealousy of the wife." (51)
- "In Case 6 morbid jealousy was a central persistent delusion of a chronic organic psychosis of 15 years' duration." (51)
- "Cases 3, 5, **6**, 7, 9, 10, 13, 14 **had not deteriorated over the years of the follow-up of this study**, and indeed according to relatives' accounts they had **deteriorated little since the end of their fighting careers**." (52)

Case 7 (Age: Not mentioned: Sport: Boxing)
Age started: Not mentioned
Age retired: Not Mentioned
Length of career: Not mentioned
Age of symptom onset: Not Mentioned
Delay between retirement and symptom onset (Age of symptom onset – Age retired): Not Mentioned

- "…there was **no evidence of progression of the neurological syndrome** in eight cases (3, 5, 6, **7**, 9, 10, 13, 14)." (47)
- "A **defect of memory** affecting the **retention and immediate recall of recent events** was present in 11 cases (2, 3, 5, 6, **7**, 8, 9, 10, 11, 13, 15) and has usually been present since the end of the boxer's career." (47)
- "Seven cases (2, 3, 4, 5, **7**, 12, 14) **remained well adjusted** in marriage and occupation and showed **no personality deviation**." (48)
- "Cases 3, 5, 6, **7**, 9, 10, 13, 14 **had not deteriorated over the years of the follow-up of this study**, and indeed according to relatives' accounts they had **deteriorated little since the end of their fighting careers**." (52)

Case 8 (Age: Not mentioned; Sport: Boxing)
Age started: Not mentioned
Age retired: Not Mentioned
Length of career: Not mentioned
Age of symptom onset: Not Mentioned
Delay between retirement and symptom onset (Age of symptom onset – Age retired): Not Mentioned

- "**Progression in severity of the neurological symptoms** was evident in eight cases (1, 2, **8**, 11, 12, 15, 16, 17) and was confirmed in the four years of the follow-up… **Ataxia** combined with **festination of gait** were the most disabling features." (45-47)
- "A **defect of memory** affecting the **retention and immediate recall of recent events** was present in 11 cases (2, 3, 5, 6, 7, **8**, 9, 10, 11, 13, 15) and has usually been present since the end of the boxer's career." (47)
- "**Mental torpor**, with a **slow, sluggish response to questions** and an **absence of spontaneity in conversation**, was present in three cases (2, **8**, 15)" (47)
- "**Persistent accusations against the wife's supposed sexual infidelity** led to the primary **psychiatric referral** in five cases (1, 6, **8**, 9, 10)." The **morbid jealously persisted** in all cases at follow-up, except in Case 1, who was by then severely demented." (48)
- "Cases **8** and 10 had been **partially impotent**…" (48)
- "**Severe personality disorders** were present in four cases (1, **8**, 9, 10). (48)

- “Case 8 has a sensitive, querulous, suspicious type of personality and had always been ‘touchy.’ His **development of a morbid jealously syndrome in late life** was seen as a personality reaction related to brain damage.” (48)
- “Case 8 had an **episode of endogenous depression** at the age of 30 years which responded to E.C.T.” (49)
- “The patient’s **accusations and suspicions of the wife’s infidelity occurred episodically** and were seen as **abnormal psychogenic reactions** in sensitive personalities. It is difficult not to presume that impotence in these cases had played some part as a pathoplastic feature in determining this particular reaction…” (51-52)

Case 9 (Age: Not mentioned: Sport: Boxing)
Age started: Not mentioned
Age retired: Not Mentioned
Length of career: Not mentioned
Age of symptom onset: Not Mentioned
Delay between retirement and symptom onset (Age of symptom onset – Age retired): Not Mentioned

- “…there was **no evidence of progression of the neurological syndrome** in eight cases (3, 5, 6, 7, **9**, 10, 13, 14).” (47)
- “A **defect of memory** affecting the **retention and immediate recall of recent events** was present in 11 cases (2, 3, 5, 6, 7, 8, **9**, 10, 11, 13, 15) and has usually been present since the end of the boxer’s career.” (47)
- “**Persistent accusations against the wife’s supposed sexual infidelity** led to the primary **psychiatric referral** in five cases (1, 6, 8, **9**, 10).” The **morbid jealously persisted** in all cases at follow-up, except in Case 1, who was by then severely demented.” (48)
- “**Severe personality disorders** were present in four cases (1, 8, **9**, 10). In three of these (1, **9**, 10) **impulsive aggressive behavior was prominent as a life-long trait** and justified the description of ‘**explosive, psychopathic personality**.’ **Rage reaction**, **uncontrolled outbursts of anger and violence**, were prominent in the case histories of these men after their boxing careers and were attributed by them to their decreased tolerance to alcohol. All three cases had had numerous emergency **psychiatric admissions for impulsive acts of violence,** of short duration.” (48)
- “Occupational adjustments related to the extent of the **neurological and psychiatric disability**. Eight patients (1, 6, **9**, 11, 13, 15, 16, 17) were permanently unemployed.” (48)
- “**Transient paranoid-hallucinatory states** occurred in Cases **9** and 12. Case 9 has **twenty emergency psychiatric admissions** because of **aggressive post-ictal behavior**, **related to post-traumatic epilepsy** which commenced at the end of his boxing career.” (49)
- “Aggression must be a prominent personality characteristic of any successful pugilist. Three cases (1, **9**, 10) had always been described as **sensitive, suspicious, impulsive and aggressive**, particularly when primed with alcohol, and all justified the label of “explosive psychopaths.” (51)
- “It is noteworthy in this series that only four were heavy social drinkers (Cases **9**, 10, 11, 13).” (52)
- “Cases 3, 5, 6, 7, **9**, 10, 13, 14 **had not deteriorated over the years of the follow-up of this study**, and indeed according to relatives’ accounts they had **deteriorated little since the end of their fighting careers**.” (52)

Case 10 (Age: 40; Sport: Boxing)
Age started: Not mentioned
Age retired: 33 years
Length of career: Not mentioned
Age of symptom onset: Not mentioned
Delay between retirement and symptom onset (Age of symptom onset – Age retired): Not mentioned

- "…there was **no evidence of progression of the neurological syndrome** in eight cases (3, 5, 6, 7, 9, **10,** 13, 14)." (47)
- "A **defect of memory** affecting the **retention and immediate recall of recent events** was present in 11 cases (2, 3, 5, 6, 7, 8, 9, **10**, 11, 13, 15) and has usually been present since the end of the boxer's career." (47)
- "**Persistent accusations against the wife's supposed sexual infidelity** led to the primary **psychiatric referral** in five cases (1, 6, 8, 9, **10**). The **morbid jealously persisted** in all cases at follow-up, except in Case 1, who was by then severely demented." (48)
- "Cases 8 and **10** had been **partially impotent**…" (48)
- "**Severe personality disorders** were present in four cases (1, 8, 9, **10**). In three of these (1, 9, **10**) **impulsive aggressive behavior was prominent as a life-long trait** and justified the description of '**explosive, psychopathic personality**.' **Rage reaction**, **uncontrolled outbursts of anger and violence**, were prominent in the case histories of these men after their boxing careers and were attributed by them to their decreased tolerance to alcohol. All three cases had had numerous emergency **psychiatric admissions for impulsive acts of violence,** of short duration…" (48)
- "A 40-year-old former bantam-weight had 200 recognized professional bouts and about 500 unrecognized contests on fair grounds and boxing booths up to the age of 33." (48)
- "He had **always** been **an impulsive, aggressive person**…" (48)
  Note: The author mentions how this case had always been impulsive. Therefore, his violent outbursts may not be completely attributable to sequalae of repetitive neurotrauma.
- "During his Army Service the patient was court-martialled and discharged as "psychopathic". He was **frequently involved in drunken brawls**, and was well known to become 'fighting mad' after a few drinks. **He had three prison sentences on account of his behaviour, and finally, while serving a prison sentence for attacking his second wife, he was transferred to a mental hospital under Section 72**. His first two marriages ended in divorce because of his **violent outbursts based upon his constant suspicion of his wives' infidelity, associated with his recurrent impotence**. His third marriage, since leaving hospital, is so far well adjusted, although his sensitive, suspicious attitudes have to be tactfully countered by his wife. **He has a marked amnesia**, **slurred speech** and **bilateral cerebellar signs in his upper limbs**. According to records of his previous neurological examinations **these symptoms are not progressive**." (48)
- "Diagnosis: **Aggressive psychopathic personality** associated with brain damage and alcoholism." (48)
- "Aggression must be a prominent personality characteristic of any successful pugilist. Three cases (1, 9, 10) had always been described as **sensitive, suspicious, impulsive and aggressive**, particularly when primed with alcohol, and all justified the label of "explosive psychopaths." (51)
- "It is noteworthy in this series that only four were heavy social drinkers (Cases 9, **10**, 11, 13)." (52)
- "The patient's **accusations and suspicions of the wife's infidelity occurred episodically** and were seen as **abnormal psychogenic reactions** in sensitive personalities. It is difficult not to presume that impotence in these cases had played some part as a pathoplastic feature in determining this particular reaction." (51-52)
- "Cases 3, 5, 6, 7, 9, **10**, 13, 14 **had not deteriorated over the years of the follow-up of this study**, and indeed according to relatives' accounts they had **deteriorated little since the end of their fighting careers**." (52)

Note: The author notes that "it is difficult not to presume that impotence in these cases had played some part as a pathoplastic feature in determining [morbid jealousy and/or accusations and suspicions of the wife's infidelity] in these brain-damaged boxers." (51-52)

Case 11 (Age: Not mentioned; Sport: Boxing)
Age started: Not mentioned
Age retired: Not Mentioned
Length of career: Not mentioned
Age of symptom onset: Not Mentioned
Delay between retirement and symptom onset (Age of symptom onset – Age retired): Not Mentioned

- "**Progression in severity of the neurological symptoms** was evident in eight cases (1, 2, 8, **11**, 12, 15, 16, 17) and was confirmed in the four years of the follow-up… **Ataxia** combined with **festination of gait** were the most disabling features." (45-47)
- "A **defect of memory** affecting the **retention and immediate recall of recent events** was present in 11 cases (2, 3, 5, 6, 7, 8, 9, 10, **11**, 13, 15) and has usually been present since the end of the boxer's career." (47)
- "Occupational adjustments related to the extent of the **neurological and psychiatric disability**. Eight patients (1, 6, 9, **11**, 13, 15, 16, 17) were permanently unemployed." (48)
- "It is noteworthy in this series that only four were heavy social drinkers (Cases 9, 10, **11**, 13)." (52)

Case 12 (Age: Not mentioned; Sport: Boxing)
Age started: Not mentioned
Age retired: Not Mentioned
Length of career: Not mentioned
Age of symptom onset: Not Mentioned
Delay between retirement and symptom onset (Age of symptom onset – Age retired): Not Mentioned

- "**Progression in severity of the neurological symptoms** was evident in eight cases (1, 2, 8, 11, **12**, 15, 16, 17) and was confirmed in the four years of the follow-up…**Ataxia combined with festination of gait** were the most disabling features, and in Case 12 had led to **invalidism in a motorized chair**." (45-47)
- "…progressive neurological symptoms rendered the patient a physical invalid, yet there was **no evidence of psychic impairment either clinically or psychometrically**." (47)
- "Seven cases (2, 3, 4, 5, 7, **12**, 14) **remained well adjusted** in marriage and occupation and showed **no personality deviation**. Case 12 was particularly outstanding in his normal personality adjustment in spite of **severe, progressive neurological disability**." (48)
- "**Transient paranoid-hallucinatory states** occurred in Cases 9 and **12**… Case 12 had a single acute paranoid-hallucinatory psychosis after taking Preludin for slimming; there was no residual personality defect." (49)
    - Note: It seems that this psychosis was due to the medication, not repetitive neurotrauma.

Case 13 (Age: Not mentioned: Sport: Boxing)
Age started: Not mentioned
Age retired: Not Mentioned
Length of career: Not mentioned
Age of symptom onset: Not Mentioned
Delay between retirement and symptom onset (Age of symptom onset – Age retired): Not Mentioned

- "there was **no evidence of progression of the neurological syndrome** in eight cases (3, 5, 6, 7, 9, 10, **13**, 14)." (47)
- "A **defect of memory** affecting the **retention and immediate recall of recent events** was present in 11 cases (2, 3, 5, 6, 7, 8, 9, 10, 11, **13**, 15) and has usually been present since the end of the boxer's career." (47)
- "Occupational adjustments related to the extent of the **neurological and psychiatric disability**. Eight patients (1, 6, 9, 11, **13**, 15, 16, 17) were permanently unemployed." (48)
- "It is noteworthy in this series that only four were heavy social drinkers (Cases 9, 10, 11, **13**)." (52)
- "Cases 3, 5, 6, 7, 9, 10, **13**, 14 **had not deteriorated over the years of the follow-up of this study**, and indeed according to relatives' accounts they had **deteriorated little since the end of their fighting careers**." (52)

Case 14 (Age: Not mentioned: Sport: Boxing)
Age started: Not mentioned
Age retired: Not Mentioned
Length of career: Not mentioned
Age of symptom onset: Not Mentioned
Delay between retirement and symptom onset (Age of symptom onset – Age retired): Not Mentioned

- "…there was **no evidence of progression of the neurological syndrome** in eight cases (3, 5, 6, 7, 9, 10, 13, **14**)." (47)
- "Seven cases (2, 3, 4, 5, 7, 12, **14**) **remained well adjusted** in marriage and occupation and showed **no personality deviation**." (48)
- "Cases 3, 5, 6, 7, 9, 10, 13, **14 had not deteriorated over the years of the follow-up of this study**, and indeed according to relatives' accounts they had **deteriorated little since the end of their fighting careers**." (52)

Case 15 (Age: Not mentioned; Sport: Boxing)
Age started: Not mentioned
Age retired: Not Mentioned
Length of career: Not mentioned
Age of symptom onset: Not Mentioned
Delay between retirement and symptom onset (Age of symptom onset – Age retired): Not Mentioned

- “**Progression in severity of the neurological symptoms** was evident in eight cases (1, 2, 8, 11, 12, **15**, 16, 17) and was confirmed in the four years of the follow-up… **Ataxia** combined with **festination of gait** were the most disabling features.” (45-47)
- “A **defect of memory** affecting the **retention and immediate recall of recent events** was present in 11 cases (2, 3, 5, 6, 7, 8, 9, 10, 11, 13, **15**) and has usually been present since the end of the boxer’s career.” (47)
- “**Mental torpor**, with a **slow, sluggish response to questions** and an **absence of spontaneity in conversation**, was present in three cases (2, 8, **15**), although it was a more prominent feature in the cases with dementia…” (47)
- “Occupational adjustments related to the extent of the **neurological and psychiatric disability**. Eight patients (1, 6, 9, 11, 13, **15**, 16, 17) were permanently unemployed.” (48)

Case 16 (Age: Not mentioned; Sport: Boxing)
Age started: Not mentioned
Age retired: Not Mentioned
Length of career: Not mentioned
Age of symptom onset: Not Mentioned
Delay between retirement and symptom onset (Age of symptom onset – Age retired): Not Mentioned

- “**Progression in severity of the neurological symptoms** was evident in eight cases (1, 2, 8, 11, 12, 15, **16**, 17) and was confirmed in the four years of the follow-up. In Case 16 the neurological disability was **confirmed as progressive** from the clinical history in hospital. **Ataxia combined with festination of gait** were the most disabling features…” (45-47)
- “In those with progressive neurological disorder only two (**1** and 16) had **progressive psychiatric disability.**” (47)
- “**Mental torpor**, with a **slow, sluggish response to questions** and an **absence of spontaneity in conversation**, was present in three cases (2, 8, 15), although it was a more **prominent feature** in the cases with **dementia** (1, **16**, 17).” (47)
- “Three cases (1, 16, 17) suffered from **post-traumatic dementia** with **mental torpor** and **flat affective responses**…” (47)
- “In Cases 1 and **16**, **dementia was paralleled by progressive neurological disability**.” (47)
- “Cases **16** and 17 were **permanently in hospital.**” (47)
- “Case 16 was **so severely affected both intellectually and neurologically** that it was felt unjustified to carry out air encephalography on him.” (47)
- “Occupational adjustments related to the extent of the **neurological and psychiatric disability**. Eight patients (1, 6, 9, 11, 13, 15, **16**, 17) were permanently unemployed.” (48)
- “In three cases (1, **16**, 17) there was **dementia due to a progressive cerebral degenerative process**.” (50)

Roberts (1969) (13)

Number of Cases Described in the Article: 11*
Cases with Anger Control Problems: Cases 2 & 6; Not Mentioned: Cases 1, 3-5, & 7-11
Cases with Depression: Case 5; Not Mentioned: Cases 1-4, & 6-11
Cases with Suicidality: Not mentioned for any cases
Cases with Anxiety (Nervousness, Fear, PTSD, Obsessive-Compulsive, Worry, "Neuroses"): Not mentioned for any cases
Cases with Paranoia/Suspiciousness: Cases 1 & 6; Not Mentioned: Cases 2-5, & 7-11
Cases with Personality/Demeanor Change: Cases 2, 5, & 6; None: Cases 4, 8, & 10; Not Mentioned: Cases 1, 3, 7, 9, & 11
Cases with Substance Use: Cases 2, 4, 5, & 11; None: Cases 3, 8, & 10; Not Mentioned: 1, 6, 7, & 9
Cases with Cognitive Change: Cases: 1, 5, 6, & 11; None: Cases 2, 4, & 7-10; Unknown: Case 3
Cases Presented as Psychiatric (e.g., depression and anxiety), not Neurological: Case 1
Cases with Headaches: Cases 5 & 7; Not mentioned: Cases 1-4, 6, & 8-11
Type of Headaches Described: Not specified
Cases Described as Having a Progressive Course: Cases 1, 2, 4, 6, & 7
Cases Described as Having a Non-Progressive Course: Cases 8, 10, & 11
Unable to be Classified as Having Progressive or Non-Progressive Course**: Cases 3, 5, & 9
Delayed Onset: None: Cases 1, 2, 6, & 9-11; Unknown: Case 3; Not Mentioned Cases 4, 5, 7, & 8
Number of Cases with Autopsy Performed: 0

*As is noted by Gardner et al. (2014) and Victoroff et al. (2013), Roberts reports 37 cases. However, he only provides details on 11 of these cases (Cases 1-11). Therefore, we only included these 11 cases for the purposes of this document.

**For these cases, the evidence for a progressive course is very limited to a comment made by the boxers' wives or colleagues, the progression of only a one/a couple symptoms, or the appearance of a couple more symptoms over time. Roberts' general tone is that the disease does not have a progressive course, or progresses only very slowly, except for a few cases.

Case 1 (Age: 60; Sport: Boxing)
Age started: 15 years (professional at 16)
Age retired: 29 years
Length of career: 13 years
Age of symptom onset: Not specified, but likely within his last year of boxing, see below
Delay between retirement and symptom onset (Age of symptom onset – Age retired): Not specified, but symptom onset was likely during career

- "This man, aged 60,… had been a patient in a **mental hospital** for seven [years]." (27-28)
- "He had no serious injuries or illness in the past, apart from, towards the end of his career, what was probably cellulitis of the hand which he held responsible for the **tremor** he had been aware of since." (28)
  Note: His hand tremor is presented as a consequence of cellulitis and not a neurological symptom of repetitive neurotrauma. Roberts doesn't say whether it is due to one or the other.
- "He had started boxing at the age of fifteen and had had about fifty amateur contests before his first professional fight at sixteen. He had fought mostly at middleweight, often every week and usually contests of twelve or fifteen three-minute rounds, with a few twenty two-minute round fights. He did not think he had been knocked out but had certainly been knocked down many times. The longest post traumatic amnesia he recalled extended for the last seven rounds of a fight which he won… he thought he had had about two hundred promoted professional contests." (28)

- “His relatives had noticed that his **speech was slurred** and that he had **developed a tremor in his left arm**, and his manager that he was **limping with his left leg in his last year boxing**. He had been **forgetful for many years**, and the **tremor in his left arm and the dragging of his left leg had become more apparent** as he grew older.” (28)
- “The death of his wife precipitated a **paranoid delusional illness** which necessitate his admission, finally for long-term care, to a mental hospital.” (28)
- “Examined he was found to be **devoid of insight and euphoric**, but it was possible to elicit some poorly systematized paranoid delusions about his wife’s relatives who he felt had dispossessed him of his inheritance. He was **demented** as judged by the **extreme slowness or this thinking and his stinking inability to register, during the course of the interview, various pieces of information**, including greetings from another boxer whom he had known well, in spite of the fact that these were several times repeated to him. His recall of details of his boxing career seemed accurate, as far as names and places were concerned, but was **hopelessly inaccurate for dates**, even those when he was champion. Tested with Raven’s Matrices he scores in the fiftieth percentile, and in the twenty-fifth percentile with the Mill Hill Vocabulary. **These tests, used on his initial admission to hospital and repeated seven years later, did not show any decline in the scores, nor had his dementia, as judges clinically and by his behavior generally, progresses notably in that time**.” (28)
- “His **speech was slurred, his face expressionless**, and there was **an increase in tone more marked in the left than the right limbs**. **All his movements were slow** and he was **markedly akinetic on attempting to walk**, **his gait thereafter short-paced and hemi-paretic with the left arm partially flexed and the leg intorted and dragging**. He was **unsteady on his feet and unable to walk tandem** fashion but there was **only dubious pyramidal weakness of the left limbs, no ataxia**. There was **an intermittent rhythmical parkinsonian rest tremor of the left limbs**. The **tendon reflexes were brisker in the left arm than the right** and **both plantar responses were extensor**. He was **normotensive and apart from a few scattered rhonchi** in his chest general examination was unremarkable.” (29)
- “In the first case described it seemed likely that his disability, due to the extra-pyramidal lesion, had **become more obvious as he grew older**, but it was **difficult to be sure that this indicated something other than the normal degenerative processes of ageing**. His **dementia had clearly progressed over the years**, and the **development of a paranoid illness** in the setting of his dementia was a certain measure of this.” (44)

Case 2 (Age: 50; Sport: Boxing)
Age started: 12 years (professional at 18)
Age retired: Likely 31 years (10-year professional career followed by 3 years in boxing booths)
Length of career: 19 years
Age of symptom onset: Likely 28 years (since his last promoted (i.e., professional) fight)
Delay between retirement and symptom onset (Age of symptom onset – Age retired): -3 years*
*Symptom onset during career

- “He fought mostly at lightweight, ten and twelve two-minute round contests and denied having been knocked out.” (29)
- “His wife had noticed that **his speech had been slurred** and **he had been unsteady on his feet since his last promoted fight** in which he had received a severe beating. He had been admitted to hospital for observation at the time and had **remained unwell for several days**. **His condition had remained little changed** except that he had become increasingly sensitive to alcohol, small quantities apparently making him very drunk, until the **gradual development ten years later of a progressively worsening tremor of his limbs**. **Although his memory did not seem to have deteriorated, he had become increasingly liable to outbursts of uncontrollable temper**.” (30)

- “Examined, he was found to be **emotionally labile, tearful and laughing alternately during the course of the interview**. His **memory**, as judged by his recall of the details of his career and the development of his symptoms, **was not grossly impaired**, but formal psychometric tests were not done.” (30)
- “His **speech was so slurred as to be almost unintelligible**, **his face was impassive**, and there was a **severe asymmetrical parkinsonian tremor of all four limbs, left more than right at rest, increased by emotion and abolished on volition. Tremor also affected his head and trunk**, his **gait was unsteady and festinant**, and **his limbs were rigid**, **more so on the left than the right**. **All tendon reflexes were pathologically brisk**, there was **sustained clonus at knees and ankles**, and **both plantar responses were extensor**. The **visual acuity in the left eye was impaired to perception of finger movements by an extensive retinal detachment** and **conjugate elevation of the eyes was poor**. There was irregularity of calibre of the arteries of the fundi keeping with his hypertension of 210/120.” (30)
- “The history was **quite clear on the point of progression** in two of these, the **second** and fourth cases [Case 4], in that an **extra-pyramidal tremor had made its appearance some years later** on a background of established dysarthria first noticed during their last years boxing.” (44)

Case 3 (Age: 58; Sport: Boxing)
Age started: 14 years (professional at 15)
Age retired: 30 years
Length of career: 16 years
Age of symptom onset: Not specified (The past ten years (i.e., since age 48) and perhaps longer, see below)
Delay between retirement and symptom onset (Age of symptom onset – Age retired): Not specified

- “Over the course of the next fifteen years he had over three hundred promoted professional contests of which he thought he had lost less than a quarter. Most were as a lightweight and were twelve or fifteen two-minute round contests. He had been knocked unconscious once for a few seconds, and the only fights stopped against him were for cut eyes. He recalled one lengthy post traumatic amnesia extending for the last three rounds of a fight…” (31)
- “He retired at the age of thirty when he began slowing up and losing too many fights.” (31)
- “Both he and his wife had been aware that **his speech, for at least the past ten years and perhaps longer,** had been **‘thick and getting thicker.’**” (31)
- “Examined, it was apparent that his **mentation was extremely slow** and that he had **difficulty recalling names and places in his boxing career**. He scored in the fiftieth percentile on Raven’s Matrices, learned his digit span of seven plus one at the fourth repetition, recalled accurately only one of the pair of Binet designs at the first attempt, but both at the second presentation and both accurately after fifteen minutes delay.” (31)
- **“His cerebellar dysarthria was so marked as to make his speech almost unintelligible at times**, there was a **mild degree of ataxia of gait on beginning to walk**, **on getting out of a chair and on turning, and the left leg was slightly circumducted walking**. There was a **terminal intention tremor in both arms and all movements were markedly slow**. **An inconstant rhythmical tremor, apparent at rest and suppressed on volition, was evident in the right arm when he was engaged in tests of intellectual function**. The only focal weakness present was in **the right triceps which was wasted and this was associated with a depressed triceps tendon reflex**. The **left plantar response was extensor**. He was **normotensive and general examination was otherwise unremarkable**.” (31)

Case 4 (Age: 48; Sport: Boxing)
Age started: 9 years (professional at 18)
Age retired: 30 years
Length of career: 21 years
Age of symptom onset: Not specified ("speech thick since his last years in boxing," see below)
Delay between retirement and symptom onset (Age of symptom onset – Age retired): Not mentioned

- "He had fought regularly, almost every week during the busiest period of his career, and recalled once having five fights in ten days. He had never been knocked unconscious but had been counted out several times. He recollected several post traumatic amnesias lasting several rounds." (32)
- "His wife had been aware of no alteration in this speech or personality, but **he himself thought that his speech had been 'a bit thick' since his last years boxing**, and he had been very conscious of the fact, and his children commented on it, that **increasingly over the years small quantities of alcohol had made him look and feel drunk**. When, during the course of the examination, **an inconstant parkinsonian tremor of his right arm was drawn to his attention, he claimed it had been present only for a few months**. He **felt that this memory had deteriorated in recent years**, so that he had great difficulty remembering people's names and errands his wife gave him." (32)
- "Examined, there was **no overt intellectual abnormality** and he appeared to have **good insight into his minor disabilities**. He scored in the twenty-fifth percentile on Raven's Matrices and on the synonym selection test of the Mill Hill Vocabulary. He learnt his digit span plus one at the third repetition, but his immediate recall of one of a pair of Binet designs was inaccurate, though after fifteen minutes delay he was able to reproduce faultlessly one of the original designs and his own version of the other." (32)
- "His **speech was slurred, his face expressionless, and there was an inconstant rhythmical parkinsonian tremor of his right arm at rest, abolished on volition**. The **tendon reflexes were brisker in the right than the left arm but the tone was only marginally increased in the right arm and both plantar responses were flexor**. There was **no apparent disequilibrium**, and his **gait was normal**. He was **normotensive** and, apart from clinical evidence of chronic bronchitis, **general examination was unremarkable**." (33)
- "The history was **quite clear on the point of progression** in two of these, the second [Case 2] and **fourth** cases, in that an **extra-pyramidal tremor had made its appearance some years later** on a background of established dysarthria first noticed during their last years boxing." (44)

Case 5 (Age: 53; Sport: Boxing)
Age started: 15 years (professional at 18)
Age retired: Not mentioned
Length of career: 10 years (professional only)
Age of symptom onset: Not mentioned
Delay between retirement and symptom onset (Age of symptom onset – Age retired): Not mentioned

- "He was discharged from the services as punchdrunk." (33)
- "He had once been involved in a road traffic accident in which he had been knocked unconscious for a few seconds. He drank excessively and had done for years… He had finally been divorced by his wife because of his **increasingly trying behavior**." (33)
- "He had ninety-six promoted fights in the course of the next ten years, mostly at light heavyweight, and had been knocked unconscious on two occasions, for seconds only at a time, though it had been necessary for him to retire on half a dozen occasions when he was taking a severe beating." (33)
- "The records made by the neurologist who had examined him twenty years earlier, during the war, were available. At the time his **speech was slurred** and **he was complaining of headaches**, **loss of memory and depression**. An independent account was also available from his present general practitioner who had known him as a **chronic alcoholic for many years**." (33)

- “Examined, **he appeared apathetic, his speech slurred and his mentation slow**. **His memory for the details of his professional career was very poor**, judging by the record he had kept, and he had considerable difficulty grasping news of former colleagues mentioned to him during the course of the interview. No formal psychometric tests were used, but according to his own account of his poor scholastic performance at school **he was constitutionally below average intelligence**, **though his evident memory difficulties and slow mentation were a certain measure of a degree of dementia superimposed.”** (33)
- “**In addition to his dysarthria, his face was expressionless, he was markedly unsteady on his feet and there was a moderate ataxia in the left more than the right arm.** There was **some spasticity in the pronators of the forearms and wrists, on the left more than the right**, the **tendon reflexes were brisker in the left limbs**, and the **left plantar response was extensor**.” (34)
- “General examination was precluded by the patient’s unwillingness to co-operate further.” (34)
- “…it seemed that the **ataxia** and evidence of **pyramidal lesions were new developments**, though the **dysarthria and sluggish mentation were not**.” (45)

Case 6 (Age: 57; Sport: Boxing)
Age started: 17 years
Age retired: 27 years
Length of career: 10 years
Age of symptom onset: 27 years
Delay between retirement and symptom onset (Age of symptom onset – Age retired): 0 years*
*No delay

- “… had been unemployed for twenty years since a mining accident. He had been buried in a fall of rock which had fractured lumbar vertebrae and left tibia and fibula, and caused a cauda equina or conscious lesion… There was a post traumatic amnesia of about eight hours though he was not unconscious when admitted to hospital according to the notes made at the time.” (34)
- In the ten years of his career he had a hundred and ten promoted professional contests, mostly at heavyweight, admitted to several extensive post traumatic amnestic episodes and to being knocked out on four occasions but no longer than seconds each time.” (34)
- “He retired from the ring, at the age of twenty-seven, when his wife and friends told him he was **getting ‘punchy.’**” (34)
- “His wife and a professional colleague, who were interviewed, and the individual himself had been aware that he **dribbled and that his speech was slurred and his gait unsteady since his last years in the ring**. The wife felt that his **speech had remained unchanged,** and certainly his **dribbling had cleared up, until his mining accident. Since then his speech and gait had progressively deteriorated and his memory, always poor, had become so defective that in recent years he did not know what day it was**. The professional colleague, who had known him well, did **not think there had been much change in the years immediately following the mining accident**, and said it had been **well recognized locally that the man had been made punch drunk** by the champion heavy weights he had sparred with for too long**. In recent years he had become increasingly irritable and liable to outbursts of uncontrollable temper**, and **two years earlier had had a severe paranoid delusional illness**, making unfounded accusations against his wife.” (35)
- “Examined, he was **euphoric and evidently demented, but insight was retained and he asserted that he was punchdrunk** and that ‘punchdrunk is a terrible thing.’ His **mentation was markedly slow** and he was **unable to recall any details of his boxing career without considerable prompting**. He was **disorientated in time and place.** He scored in the twenty-fifth percentile in Raven’s Matrices and in the synonym selection test of the Mill Hill Vocabulary. His digit span was six but he was quite unable to learn another digit in addition, and although his copy of the Binet designs was accurate, he was unable to recall either.” (35)
- “There was a **mild dysphasia, his speech was so slurred as to be occasionally unintelligible**, his **face was impassive,** and there was **nystagmus on lateral gaze.** His **gait was unsteady and high-stepping and there**

was titubation of head and trunk. All four limbs were ataxic, more so in the right arm and left leg. There was **slight spasticity in the pronators of the right forearm and right quadriceps**, the **tendon reflexes were brisker on that side**, and **both plantar responses were indefinite extensors**" (35)
- "A small area of sacral sensory loss, depressed ankle and absent left knee jerks, together with his urinary incontinence, which necessitates wearing a urinal, were sequelae of the conus or root lesions caused by the mining accident." (35)
  - Note: Roberts (1969) identifies these symptoms as sequalae of this patient's mining accident, and does not attribute them to repetitive neurotrauma.
- "General examination was unremarkable apart from evidence of chronic bronchitis and a blood pressure of 145/105." (35)

Case 7 (Age: 57; Sport: Boxing)
Age started: 12 years (professional at 18)
Age retired: 26 years
Length of career: 12 years
Age of symptom onset: Not mentioned
Delay between retirement and symptom onset (Age of symptom onset – Age retired): Not mentioned

- "This man, aged 57, had been a labourer and subsequently a driver for many years until a minor head injury, which did not cause loss of consciousness, **incapacitated him with headaches** and phobic symptoms, necessitating his retirement." (35)
- "He denied ever being knocked out or experiencing any memory disturbance during his career." (35)
- "His wife had noticed **for years that his speech was slurred when he was excited**, but thought that **this had become much more obvious since his mild head injury**, **since when she had also noticed that he was unsteady on his feet and tended to drag his left leg**." (36)
  - Note: A description of the mild head injury, referred to above, is provided here: "a minor head injury, which did not cause loss of consciousness, incapacitated him with **headaches and phobic symptoms**, necessitating his retirement [from being a driver]." (35-36)
- "Examined, **apart from his poor recall of the details of his boxing career**, there was **no overt clinical evidence of intellectual defect**. He scores at the fiftieth percentile in the Raven's Matrices and in the synonym selection test of the Mill Hill Vocabulary, learned his digit span of five plus one at the second repetition and recalled accurately, both immediately and after fifteen minutes delay, both Binet designs." (36)
- "His **speech was markedly slurred,** his **gait was unsteady with a mild degree of truncal ataxia,** and there was an **asymmetrical ataxia of the left limbs more than the right**. He was **generally hypotonic**, though the **tendon reflexes were brisk**, **more so on the left than the right**. **Both plantar responses were flexor**. **The only weakness present was characteristic of a ruptured supraspinatus tendon. General examination was unremarkable** apart from a blood pressure of 145/100." (36)
- "The evidence for progression in these lesions among this group [cases similar to Case 7, not described in detail by Roberts (1969)] was poor except in the case of the individual reported in detail [Case 7] whose **condition seemed to have worsened**, **or at least become more noticeable** to his relatives, after an apparently trivial head injury…" (45)

Case 8 (Age: 45; Sport: Boxing)
Age started: 11 years (professional at 17)
Age retired: Not specified, likely 30 years (13-year professional career starting at age 17)
Length of career: 13 years (professional only)
Age of symptom onset: Not mentioned
Delay between retirement and symptom onset (Age of symptom onset – Age retired): Not mentioned

- "In the next thirteen years he had fought a hundred and thirty-seven professional contests, mostly at bantamweight, and at his busiest as many as twenty a year. He had been knocked out for seconds only on three occasions, but had once felt dazed for an hour afterwards." (37)
- "**His wife commented that he was forgetful and agreed that his speech was slurred**, **but did not think there had been any change in the latter or in his equable temperament since she had known him a year before he became a professional boxer**. **He himself had noted with surprise that his speech was slurred** when he had tape recorded himself, but did not think it was due to boxing, and was aware of no other disabilities." (37-38)
- "Examined, **he seemed mildly euphoric**, but there was **no clinical suggestion of intellectual impairment, and his memory, as judged by his detailed recall of his career, was good**. He scored in the ninety-fifth percentile in Raven's Matrices, and in the fiftieth percentile in the synonym selection test of the Mill Hill Vocabulary. He learned his digit span of six plus one at the third repetition, and his recall of the Binet designs, both immediate and delayed, was accurate." (38)
- "There was an **obvious slurring dysarthria**, his **face was expressionless**, and **in repose a mild inconstant ocular divergence made him appear glassy eyed**. **All his movements were slow**, and **repetitive movements of his arms were a little clumsily performed**, but there was **no definite ataxia.** He was **unsteady walking tandem and on turning**, and his **gait was mildly spastic**, **as were his forearm pronators and both quadriceps, more so in the left limbs than the right**. The **tendon reflexes were pathologically but symmetrically brisk**, there were **a few beats of clonus at both ankles**, but the **plantar responses were flexor and the jaw jerk normal**. There was **no weakness and no sensory deficit**. He was normotensive and **general examination was unremarkable**." (38)

Case 9 (Age: 58; Sport: Boxing)
Age started: 13 years (professional at 19)
Age retired: 28 years
Length of career: Not mentioned
Age of symptom onset: Not specified (unsteady on feet during last years boxing, see below)
Delay between retirement and symptom onset (Age of symptom onset – Age retired): No delay (see age of symptom onset)

- "…had been a driver for thirty years since his retirement from the ring. A philanthropic admirer, who had followed closely his boxing career to the end, had offered him the job telling him it was pitiful to see what had become of him." (39)
- "He had been knocked out on three occasions, but for seconds only though he recalled a few long post traumatic amnesias lasting for several hours, coming to on each occasion at home or on his way there." (39)
- "**His wife considered him punchdrunk in that his speech was slurred when he was excited**, and **he dribbled and had done for years**. He himself was not aware of this but had noticed that he was **unsteady on his feet during his last years boxing and that this had become worse in recent years so that he often lost his balance**. He was aware that **his memory was poor**." (39-40)
- "Examined, there was **no impression of intellectual defect clinically,** and he was **notably insightful and frank.** He recalled the details of his boxing career accurately except for the dates. He learnt his digit span of six plus one at the fifth repetition, and his immediate recall of the two Binet designs was accurate, though he was unable to recall one of the figures accurately after fifteen minutes delay. His immediate recall of a

flower, a colour and an address were accurate. He scored in the fiftieth percentile in the Raven's Matrices and in the twenty-fifth on the synonym selection test of the Mill Hill Vocabulary." (40)

- "His **speech was slurred and his gait unsteady, particularly on turning**. There was **slight clumsiness, not amounting to definite ataxia, in all four limbs, but no weakness**. **Slight spasticity was evident in both quadriceps** and the **tendon reflexes were pathologically but symmetrically brisk**. The **left plantar response was an indefinite extensor and the right flexor**. He was normotensive, and **apart from clinical evidence of severe chronic bronchitis and emphysema general examination was unremarkable**." (40)

Case 10 (Age: 35; Sport: Boxing)
Age started: 11 years (professional at 19)
Age retired: Not mentioned
Length of career: Not mentioned
Age of symptom onset: Not specified (Speech slurred about the time he retired, see below)
Delay between retirement and symptom onset (Age of symptom onset – Age retired): Possibly no delay (see age of symptom onset)

- "He was knocked unconscious once, did not think he had been carried from the ring, but recalled that the post traumatic amnesia lasted till he was in the dressing room." (41)
- "**His wife was aware of no change in his amiable and equable temperament** in the years she had known him, **considered his memory as good as her own** and **had never seen him unsteady on his feet**. She admitted that **she had noticed that his speech was slurred at about the time he retired from boxing, but did not think there had been any change, for better or worse, since**." (41)
- "Examined, he was **undoubtedly jovial but by no means pathologically so**, and there was **no clinical impression of intellectual defect**. His history of his boxing career was accurate and detailed. He learnt his digit span of seven plus one at the fifth repetition. His immediate and delayed recall of the two Binet designs was accurate. He scored at the twenty-fifth percentile in Raven's Matrices, and at the fiftieth in the synonym selection test of the Mill Hill Vocabulary" (41)
- "The **only evidence of neurological lesions was a slight, but quite definite, slurring dysarthria, and an extensor plantar response on the left**." (41)
- "There was **no suggestion of progression** in any of these cases [Cases 10 and 11 and seven similar cases]." (46)

Case 11 (Age: 39; Sport: Boxing)
Age started: 17 years (professional at 17)
Age retired: Not mentioned
Length of career: Not mentioned
Age of symptom onset: Not specified, but likely at the time of retirement as he retired "when, on medical grounds, renewal of his license was refused." (42)
Delay between retirement and symptom onset (Age of symptom onset – Age retired): No delay

- "He had been knocked out on three occasions, for seconds only, but admitted to feeling groggy for short periods during many fights." (42)
- "An independent account was not available from his wife, but the findings of a neurologist, who examined him when the question of renewal of his license was under consideration **fifteen years earlier**, were. **He had been noted then to be dysarthric and very slow in cerebration**. **He himself was aware that his memory was appalling** having for several years to write everything down he needed to remember, and commented on the fact that **it had always astonished him how slurred his speech was when he heard it on tape recordings**." (42-43)
- "Examined, **it was evident that his memory, judged by his recall of the details of his boxing career, was poor**, indeed the area championship he claimed to have held was not the one he had. **His mentation was**

not, however, notably slow, and he scored in the fiftieth percentile in Raven's Matrices and in the twenty-fifth in the synonym selection test of the Mill Hill Vocabulary. He learned his digit span of seven plus one at the second repetition but was unable to recall accurately, either immediately or after fifteen minutes delay, more than one of the pair of Binet designs." (43)

- "He had a **severe slurring dysarthria, but there were no other abnormal physical signs in his central nervous system** and he was clearly otherwise extremely fit." (43)
- "There was **no suggestion of progression** in any of these cases [Cases 10 and 11 and seven similar cases]." (46)

Corsellis, Bruton, and Freeman-Browne (1973) (14)

Number of Cases Described in the Article: 15*
Cases with Anger Control Problems: Cases 1, 3, 5-8, & 10-13; Not Mentioned: Cases 2, 4, 9, 14, & 15
Cases with Depression: Not mentioned for any cases
Cases with Suicidality: Case 9; Not Mentioned: Cases 1-8 & 10-15
Cases with Anxiety (Nervousness, Fear, PTSD, Obsessive-Compulsive, Worry, "Neuroses"): Not mentioned for any cases
Cases with Paranoia/Suspiciousness: Cases 3, 6, 10, & 13; Not Mentioned: Cases 1, 2, 4, 5, 7-9, 11, 12, 14, & 15
Cases with Personality/Demeanor Change: Cases: 1-3, 6-8, 10, &13; Not Mentioned: Cases 4, 5, 9, 11, 12, 14, &15
Cases with Substance Use: Cases 1, 3-10, & 13; None: Cases 2, 14, &15; Not Mentioned: 11 & 12
Cases with Cognitive Change: Cases 1-11, & 13; None: Cases 12 &14; Not Mentioned: Case 15
Cases Presented as Psychiatric (e.g., depression and anxiety), not Neurological: Case 9
Cases with Headaches: Case 7; Not Mentioned: Cases 1-6 & 8-15
Type of Headaches Described: "Violent" (Case 7)
Cases Described as Having a Progressive Course: Cases 1-8, 10 & 13
Cases Described as Having a Non-Progressive Course: Cases 12, 14, & 15
Unable to be Classified as Having Progressive or Non-Progressive Course: Cases 9 & 11
Delayed Onset: Cases 8 & 11; None: Cases 2-6; Not Mentioned: 7, 9, 10, & 12-15
Number of Cases with Autopsy Performed: 15 (Autopsy performed on all cases)

Case 1 (Age: 63; Sport: Boxing)
Age started: 11 years
Age retired: Not mentioned, likely 25 years
Length of career: 14 years
Age of symptom onset: Not mentioned, likely in his 20s (see below)
Delay between retirement and symptom onset (Age of symptom onset – Age retired): Not mentioned

- "As a young man he was quiet, generous, and abstemious. He married in his early 20s… soon his life became more hectic and 'he changed completely.' He **wenched and drank and gambled heavily**. His **memory began to fail him**." (271)
- "He had three car accidents; in one he suffered severe scalp lacerations and an injury to the right eye, followed by three months in hospital." (271)
- "His marriage broke up and he **drifted away from his family**…" (271)
- "He had **violent outbursts**, he was **'knocked out' by only a small amount of alcohol**, his **behavior was 'disgusting.'** His brother remarked that 'his brain was not functioning – he made **mistakes in reckoning**.' He could not settle in a job and he became **vagrant**." (271)
- "At the age of 62 he was found lying neglected and louse-ridden in the boiler house of a hotel. In hospital he could answer questions to the point but his **speech was slurred and indistinct**. He was continent. A routine physical examination revealed a **right-sided ptosis** and a **cataract of the right eye**. He was **unsteady on his feet**." (271)

Case 2 (Age: 77; Sport: Boxing)
Age started: Not specified, “boxed from boyhood” (272)
Age retired: early 30s
Length of career: Not mentioned
Age of symptom onset: Not mentioned, likely during or before his earl 30s (see below)
Delay between retirement and symptom onset (Age of symptom onset – Age retired): No delay

- “He retired in his early 30s after a severe battering and was **described at that time as ‘a little old man**… playing the part of an animated punching bag…’” (272)
- “**By the age of 50 he staggered** slightly when he walked and **his speech was slow and slurred.**” (273)
- “**During the following decade he became childlike**, often wanting to be cuddled and reassured. He **suffered two head injuries** which were not severe but **they may have aggravated the deterioration in memory and in behaviour**…” (273)
- “**When he was 67** he developed an acute appendicitis and was noticed to be **confused, incontinent of urine, and disoriented; his memory was severely impaired**. **His manner was still childish**; he would sulk when left alone and would smile happily when approached. He retained some idea of his boxing cusses but had forgotten the details. He **spoke indistinctly**. He was **markedly ataxic,** walking on a wide base and often stumbling. He **could not stand on one leg** and **heel-toe walking led him to fall to the right**. He had **nystagmus to the right and a tremor of his upper limbs**. Muscle power, tendon reflexes, pupillary responses, and blood pressure were all normal.” (273)
- “He died demented and doubly incontinent, in a psychiatric hospital at the age of 77.” (273)

Case 3 (Age: 63; Sport: Boxing)
Age started: 16 years
Age retired: Not specified, likely just soon before 30 years (see below)
Length of career: 13 years
Age of symptom onset: Not specified, likely around time of retirement, according to wife (see below)
Delay between retirement and symptom onset (Age of symptom onset – Age retired): No delay

- “He suffered a neck injury (unrelated to boxing) when about 26 and boxed less after this, becoming a manager when approaching 30. His wife met him shortly after this and she recalled that **‘he had already begun to get a bit muddled.’ By the age of 36 he would often fall backwards. He began to suspect his mother of theft and his wife of infidelity**.” (274-275)
- “He was seen in hospital **when aged 44** because of back pain. Osteoarthritis of his cervical vertebrae was found on x-ray; **a rigid gait was noted, both arms were slightly spastic; early Parkinsonism was queried**.” (275)
- “**At 50, slurring of speech** was mentioned and the term **‘punch-drunk’** was used.” (275)
- “**Six years later** a **neurological report included an extra-pyramidal tremor of both hands and ataxia of both legs**. There was a **slight dysarthria**.” (275)
- “A further report, **when he was 61**, stated that **his unsteadiness and shaking hands had become worse**. He was ‘**dribbling a bit,**’ he had **Parkinsonian facies**, his **speech was difficult to understand**, and his **memory was poor**. His **wife recalled that he had a bad temper**, ‘**the scenes were awful**,’ ‘**he raved at night**’ but was ‘so kind and nice afterwards.” (275)
- “It was concluded in hospital that he had ‘**diffuse degenerative brain disease affecting extrapyramidal and pyramidal systems as well as causing mental impairment**.’ He **could not talk or swallow properly**; he had **several falls** and after the last one he died from bronchitis and bronchopneumonia when aged 63.” (275)

Case 4 (Age: 69; Sport: Boxing)
Age started: 15 years
Age retired: 40 years
Length of career: 25 years
Age of symptom onset: mid-30s
Delay between retirement and symptom onset (Age of symptom onset – Age retired): Symptom onset during career

- "As a young man he was considered shrewd and not unintelligent." (276)
- "In his **mid-30s he became unsteady on his feet**, and **after the age of 40 he had a shuffling gait**. His **legs were apt to give way** and he **developed a marked tremor of the hands**. He had **difficulty in dressing himself**." (276)
- "He drank heavily for a few years at this time but cut his intake down drastically when he found that he could no longer take it." (276)
- "**When he was 57** he attended hospital for backache and **was found to have a spastic gait and slurred speech**, both of which were recorded as having been present for some years. **At the age of 64** he was reinvestigated in a neurosurgical unit. A **history of progressive unsteadiness on his feet and mental deterioration with impairment of memory were described by his wife**. He **lost all sense of date and time** and **did not recognize his relatives**. His **speech was dysarthric**, he had a **wide-based ataxic gait,** and **Romberg's sign was positive.** He was considered **mildly demented**. **No tremor was recorded**. Air studies showed general enlargement of the ventricles without displacement; the septum was not mentioned, and the **punch-drunk syndrome and spinal osteoarthritis were diagnosed**. **When aged 69** he entered hospital with acute retention of urine. He had a **tremor of both legs and a mask-like facies**. He developed a chest infection and died three weeks later." (276-277)

Case 5 (Age: 61; Sport: Boxing)
Age started: As a child (professional at 18)
Age retired: Not mentioned
Length of career: 18 years
Age of symptom onset: about 30 years (see below)
Delay between retirement and symptom onset (Age of symptom onset – Age retired): Symptom onset during career

- "He was always aggressive and spiteful and was so unpopular with the crowds that they liked to see him beaten… He drank excessively in his 20s" (278)
    - Note: His aggression appeared to be a lifelong personality trait, which may not be due to his boxing career.
- "**Towards the end of his career at the age of about 30 he was no longer able to look after his personal affairs**. He **became enuretic when about 35 and eventually had to be cared for in a home**. He was noted to be **'irascible, forgetful, but clean and tidy and quite nimble on his feet**.'" (278)
- "**When 60 years old he developed a right facial weakness and a right homonymous hemianopia**. In the same year, and a few months before he died, he was **transferred to a psychiatric hospital**. Death was attributed to bronchopneumonia and cardiac ischaemia." (278)

Case 6 (Age: 83; Sport: Boxing)
Age started: 13 years
Age retired: Not specified, likely 38 years ("six years later" (279) from the age of 32)
Length of career: 25 years
Age of symptom onset: 32 years
Delay between retirement and symptom onset (Age of symptom onset – Age retired): -6 years*
*Symptom onset during career

- "He sometimes took severe punishment and was knocked out occasionally." (279)
- "He was fit **until the age of 32 when his legs 'began to give way', he developed a 'hoping-dancing walk', and his speech became slurred**. His memory was good." (279)
- "His **two marriages broke up**, largely because of his **violent nature**. When 65 years old he went to live with his son but **aggressive outbursts led to his removal to an old people's home**. He became more subdued but he still had the occasional scuffle and one of these brought him to a **psychiatric hospital**." (279)
- "On admission he was **orientated but his memory was poor**. He **thought people were robbing him**. He was **dysarthric, and ataxic, with a slow shuffling gait**. He had a **Parkinsonian look and some weakness of his left leg**. He was **incontinent of urine**. His death, at 83, was attributed to bronchopneumonia." (279)

Case 7 (Age: 62; Sport: Boxing)
Age started: 16 years
Age retired: Not specified, likely 36 years
Length of career: 20 years
Age of symptom onset: Not mentioned
Delay between retirement and symptom onset (Age of symptom onset – Age retired): Not mentioned

- "He retired from the ring after a particularly damaging fight, and started to teach physical training and boxing in boarding schools. He gave this up after a few years at a time when he was **drinking heavily and beginning to become moody and violent**." (280)
- "**When about 54 he began to be 'very unsteady on his feet' and kept falling about**. He had **spells of 'going within himself' followed by aggressive attacks on his wife and his home**. He complained of **violent headaches**; his sexual demands gave his wife little rest. He **wandered, out, half-dressed, at night and would importune for money**. He had **'glassy-looking eyes'**; he was **doubly incontinent at times**. He spoke normally and had no tremor**; he could not be left alone**. **When 59 he was admitted to a psychiatric hospital and found to be grossly demented**. The summary stated that '**examination of the CNS, including gait, revealed no abnormality**." (280)
- "The **dementia deepened and in the last two years of his life** 'he aged, became like an old man'; he developed bronchopneumonia and died at the age of 62." (280)

Case 8 (Age: 71; Sport: Boxing)
Age started: 17 years
Age retired: 40 years
Length of career: 23 years
Age of symptom onset: 60 years
Delay between retirement and symptom onset (Age of symptom onset – Age retired): 20 years

- "He **began to go downhill when he was 60 years old**. **He then lost consciousness for two hours and on recovery he was ataxic and had some weakness of his left side**. **Several similar episodes occurred during the next 10 years**; **between them he remained ataxic with a tendency to fall**. He **had a tremor** and could not tie a bow. The **last attack affected his right side**, and his **intellect and behavior then deteriorated.** He **became violent towards his wife** but would afterwards apologize. He burnt all his newspaper cuttings and then asked for them back. He would **try to go unclothed into the street**. He died at home aged 71." (281)

Case 9 (Age: 72; Sport: Boxing)
Age started: Not mentioned
Age retired: Not mentioned
Length of career: Not mentioned
Age of symptom onset: 31 years
Delay between retirement and symptom onset (Age of symptom onset – Age retired): Not mentioned

- "This man was a patient in a **psychiatric hospital for the last seven years of his life**." (282)
- "On his return to England, **when aged 31, his brother noticed that there was a tremor of his left hand and that his speech was slurred and hoarse.** The hoarseness had followed a blow to the throat." (282)
- "He **started drinking heavily and drifted from job to job**. He **tried to kill himself when aged 65** and was **admitted to a psychiatric hospital**. **A Parkinsonian facies** was noted, **with tremor and rigidity of the left upper limb**. The **left vocal chords were markedly thickened and he was dysarthric**. The blood pressure ranged around 210/130, and **he was considered to be demented possibly as a result of cerebral trauma**. A **transient left-sided hemiparesis was noticed nine months before his death** from bronchopneumonia at the age of 72 years." (282)

Case 10 (Age: 67; Sport: Boxing)
Age started: Not mentioned
Age retired: Not mentioned
Length of career: Not mentioned
Age of symptom onset: about 40 years
Delay between retirement and symptom onset (Age of symptom onset – Age retired): Not mentioned

- "[His second wife] **first noticed that his memory was poor when, aged about 40**, he was serving in her shop." (283)
- "He and his wife had **drinking bouts** and he was **known in his locality as a violent man**, although 'he had been a good husband'. At the **age of 60 he developed a left hemiparesis and was admitted to a mental hospital three years later**. He was **disorientated** and had **a marked loss of recent memory**. He was **paranoid and deluded, and became confused and aggressive**." (283)
- "The **diagnosis was of cerebral arteriosclerosis and organic dementia, probably post-traumatic in origin**. The term **'punch-drunk'** was used. He **gradually deteriorated over the next four years** and died aged 67." (283)

Case 11 (Age: 67; Sport: Boxing)
Age started: Not mentioned
Age retired: about 35 years
Length of career: Not mentioned
Age of symptom onset: 67 years
Delay between retirement and symptom onset (Age of symptom onset – Age retired): 32 years

- "During the last six years of his life **he remained bright and alert and worked regularly** as a road sweeper. **When aged 67 he was admitted to hospital in heart failure** with a **history of increasing confusion**. He was **disorientated and aggressive**; **Argyll Robertson pupils were present**." (284)
- "He died three months later, aged 67." (284)

Case 12 (Age: 91; Sport: Boxing)
Age started: Not mentioned
Age retired: Not mentioned
Length of career: Not mentioned
Age of symptom onset: Not mentioned
Delay between retirement and symptom onset (Age of symptom onset – Age retired): Not mentioned

- "He **spent his last years in a home for the partially disabled as he had become almost blind**, **for reasons that were never stated**. He was **solitary and placid** but **occasionally became aggressive**. **He was considered active and mentally alert up to the time of his death, at 91**, from cardiac ischaemia." (284)
- "It was noted at the post-mortem examination that there were no stigmata of his former profession and that he was a **'remarkably well-preserved old man.'**" (284)

Case 13 (Age: 57; Sport: Boxing)
Age started: 14 years
Age retired: Not mentioned
Length of career: Not mentioned
Age of symptom onset: Not mentioned
Delay between retirement and symptom onset (Age of symptom onset – Age retired): Not mentioned

- "He won 'several cups and many canteens of cutlery'; he took part in exhibition bouts; he was knocked out several times." (285)
- "[His daughter] recalled that he was 'always **having accidents if he rode on a bicycle**' and **when aged 34 he was concussed after an accident**. This needed outpatient treatment; he was delirious at home the following night and needed a week to recover." (285)
- "After his discharge a few years later [after rejoining the Navy when aged 43] he **became 'more vague' and 'spoke more slowly'**. He **began to neglect his appearance** and he **complained that he could not grip things or get on a bus**. His **sight and his memory seemed a little faulty**. He had never drunk alcohol excessively but **he now became violent after two pints of beer, sometimes 'fighting with his wife**.'" (285).
- "He **wandered away bemused and had to be taken to a mental hospital. He was then 53**. On admission he was **mildly confused and complained of blackouts**. His wife, from whom he was separated, but who was still concerned for him, said that **his personality had been changing for the worse.** A brief **neurological report confirmed this and mentioned a deterioration of habits**. He **could not recall his address or the date**. No incoordination was found but a **'little dyspraxia'** was queried. **During the next year his memory deteriorated further, he was completely disorientated and needed help with dressing**. These features were emphasized in a detailed neurological investigation. In addition his **speech was slurred, and like his movements was slow and tremulous**. He **could not undress himself**." (285)

- "…it was concluded that **'the cortical atrophy and dementia may be related to his previous experiences as a boxer.'**" (285)
- "The **deterioration continued**. He became **incontinent, paranoid, and aggressive**. He ate off the table with his fingers; a cigarette packet he called a flower; he undid buttons when asked to put his tongue out. He continued to have blackouts and a few days after a convulsion he died from bronchopneumonia, aged 57 years." (285)

Case 14 (Age: 61; Sport: Boxing)
Age started: Not specified, sometime after joining the Royal Air Force at age 18
Age retired: Not mentioned
Length of career: Not mentioned
Age of symptom onset: Not mentioned
Delay between retirement and symptom onset (Age of symptom onset – Age retired): Not mentioned

- "He was a placid, popular man, happily married with five children. He drank and smoked little. He suffered no injuries during his boxing career and had no serious illnesses. His wife said that his **eyesight and hearing**, his **speech and his memory were normal up to the time of his death**, from a cerebral haemorrhage, at the age of 61." (286)

Case 15 (Age: 58; Sport: Boxing)
Age started: Not mentioned
Age retired: Not mentioned
Length of career: Not mentioned
Age of symptom onset: Not mentioned
Delay between retirement and symptom onset (Age of symptom onset – Age retired): Not mentioned

- "**No neurological or psychological abnormality was noted at any time**. He neither drank nor smoked. His work record was satisfactory until he dies from a fractured skull and cerebral haemorrhage after a road traffic accident at the age of 58." (287)

Harvey & Newsom Davis (1974) (15)

Number of Cases Described in the Article: 1
Anger Control Problems: Yes Depression: Yes
Anxiety (Nervousness, Fear, PTSD, Obsessive-Compulsive, Worry, “Neuroses”): Not Mentioned
Paranoia/Suspiciousness: Yes Personality/Demeanor Change: Yes
Substance Use: Not Mentioned
Cognitive Change: Yes Suicidality: Not mentioned
Case Presented as Psychiatric (e.g., depression and anxiety), not Neurological: No
Headaches: Not mentioned
Type of Headaches Described: Not applicable
Progressive Course: Yes Non-Progressive Course: No
Unable to be Classified as Having Progressive or Non-Progressive Course: No
Delayed Onset: No Autopsy Performed: No

Case 1 (Age: 25; Sport: Boxing)
Age started: 14 years (professional at 21)
Age retired: Not specified, likely 24 years (“three and a half years later” (928) from age 21)
Length of career: Not specified, likely about 10 years
Age of symptom onset: Not specified, but after 16 professional bouts
Delay between retirement and symptom onset (Age of symptom onset – Age retired): Symptom onset during career (after 16 bouts in a 25-bout professional career)

- “As an amateur, he lost about 10 fights on points, took no severe beatings, was not knocked out, and experienced no episodes of amnesia related to bouts… At the age of 21 he became a professional… He was knocked out once, but another fight was stopped because of the injuries he was receiving.” (928)
- His family first noticed that this **speech was slurred** after he had had 16 professional bouts. **Six months later he started to shuffle slightly when walking, his legs appearing stiff**. Despite his success in his last few fights, **he was aware of his slowness on his feet** and of increasing difficulty in avoiding trouble in the ring.” (928)
- “He retired from the ring a year before his admission to hospital. During that time, **his gait and speech deteriorated.** He **developed lability of mood, becoming more aggressive and socially unstable**. An **acute depressive illness with paranoid features then necessitated his admission to a psychiatric unit**, where for a few days he received a small dose of chlorpromazine followed by trifluoperazine 5 mg. twice daily for about two weeks. It was recognized that **he also had a neurological disorder,** and he was therefore later transferred to the national Hospital at which time he had not taken phenothiazines for four weeks.” (928)
- “He was **cooperative but mentally slow**. He had a verbal I.Q. of 95 and a performance I.Q. of 88. He was **normotensive**. **Bilateral ptosis** was present. He had a **symmetrical extrapyramidal disorder** characterized by an **expressionless face, generalized poverty of movement, cogwheel rigidity in all four limbs, and typically extrapyramidal speech and gait**. His **tendon reflexes were brisk**, with **left extensor plantar and variable right plantar response**. There was no sensory deficit, and sphincter function was normal.” (928)
- “After discharge he had **another episode of disturbed behavior and had to be readmitted** for a period to a psychiatric hospital.” (928)
- “The development in the young boxer of **extrapyramidal and pyramidal dysfunction, coupled with depressive and paranoid mood swings,** leaves us in no doubt that he had the traumatic encephalopathy of boxers. This clinical diagnosis is supported by the radiological demonstration of a **cavum septum pellucidum** which is a characteristic feature of this syndrome.” (928)
- “In our patient **the onset of symptoms was at a relatively early age** and at the time in his career when most of his boxing experience had been as an amateur.” (928)

Kaste, Vilkki, Sainio, Kuurne, Katevuo, & Meurala (1982) (16)

Number of Cases Described in the Article: 14*
Cases with Anger Control Problems: Not mentioned for any cases
Cases with Depression: Not mentioned for any cases; Cases with Suicidality: Not mentioned for any cases
Cases with Anxiety (Nervousness, Fear, PTSD, Obsessive-Compulsive, Worry, "Neuroses"): Not mentioned for any cases
Cases with Paranoia/Suspiciousness: Not mentioned for any cases
Cases with Personality/Demeanor Change: None: Cases 3-14; Unknown: Cases 1 & 2
Cases with Substance Use: None: Cases 1-14 (exclusion criteria included alcoholism and heavy drinking)
Cases with Cognitive Change: Cases 1 & 2; None: Cases 3-14
Cases Presented as Psychiatric (e.g., depression and anxiety), not Neurological: 0
Cases with Headaches: Not mentioned for any cases; Type of Headaches Described: Not applicable
Cases Described as Having a Progressive Course: Not mentioned for any cases
Cases Described as Having a Non-Progressive Course: Not mentioned for any cases
Unable to be Classified as Having Progressive or Non-Progressive Course: Cases 1-14
Delayed Onset: Not mentioned for any cases
Number of Cases with Autopsy Performed: 0
*The authors report the neurological findings, psychological test findings, electroencephalogram (EEG) and brainstem auditory evoked potential (BEP) findings, and computed tomography (CT) findings of 14 boxers (8 amateurs and 6 professionals) with a mean age of 31 years, one of whom was still actively competing. Four of the amateurs had had one concussion, two of them twice outside the boxing ring. Although they summarize the findings in Table 2 (pg. 1187), they only provide descriptions for Cases 1 and 2. The descriptions of these two cases are limited and presented together, but they are included below. The neurological status and psychological test performance of all other cases were normal (Table 2, pg. 1187). The article was coded in the summary table based on the authors' overall opinions and conclusions from the entire sample and CSP findings were recorded in Appendix C. Reasons for exclusion were moderate to severe head injuries caused outside the boxing ring, exposure to organic solvents, alcoholism, heavy drinking, cerebrovascular disorders, and other known reasons for brain atrophy.

Cases 1 & 2 (Case 1 Age: 53; Case 2 Age: Not specified, between 19-53; Sport: Boxing)
Age started: Not mentioned
Age retired: Not mentioned
Length of career: Not mentioned
Age of symptom onset: Not mentioned
Delay between retirement and symptom onset (Age of symptom onset – Age retired): Not mentioned

- "Twelve of the boxers had psychological test results which suggested brain injury, although only two professionals had definite deviation from normal." (1186)
- "Only one professional (no. 1) had **abnormal neurological findings: apraxia and slight unsteadiness and slight slowness and uncertainty in mental functions were observed**. He had been treated for mild hypertension and diabetes for 2 years. He was one of two professionals (nos 1 and 2) who also had had episodes of **embarrassing inappropriate behaviour** which were attributed to their boxing careers. The neurological status of the other boxers, including subject 2, was within normal limits (table II)." (1186)
- "Two professionals (nos 1 and 2), however, showed **obvious deviations from the normal [psychological] test performance**. They both had subjective symptoms and objective signs of brain injury (Table 2)." (1187)
- "Although only one of the boxers, the oldest, had **dysfunctions that affected his normal daily living and social relations**, another, the second oldest, had already had episodes of **inappropriate behaviour** which were attributed to his boxing career." (1187)

Ross, Cole, Thompson, & Kim (1983) (17)

Number of Cases Described in the Article: 40*
Cases with Anger Control Problems: Not mentioned for any cases
Cases with Depression: Not mentioned for any cases
Cases with Suicidality: Not mentioned for any cases
Cases with Anxiety (Nervousness, Fear, PTSD, Obsessive-Compulsive, Worry, “Neuroses”): Not mentioned for any cases
Cases with Paranoia/Suspiciousness: Not mentioned for any cases
Cases with Personality/Demeanor Change: Not mentioned for any cases
Cases with Substance Use: Not mentioned for any cases
Cases with Cognitive Change: Not specified for any cases
Cases Presented as Psychiatric (e.g., depression and anxiety), not Neurological: 0
Cases with Headaches: Not specified for any cases
Type of Headaches Described: Not specified for any cases
Cases Described as Having a Progressive Course: Not mentioned for any cases
Cases Described as Having a Non-Progressive Course: Not mentioned for any cases
Unable to be Classified as Having Progressive or Non-Progressive Course: Cases 1-40
Delayed Onset: Not mentioned for any cases
Number of Cases with Autopsy Performed: 0

*The authors report the findings of their examination of 40 boxers, two of whom were still actively boxing at the time of the study. Thirty-eight underwent a head CT scan and 24 underwent a full neurological examination with electroencephalogram (EEG), of whom 22 also had a CT scan. The authors report in Table 1 that 6 boxers had positive neurological examinations. However, they do not report which signs or symptoms contributed each positive exam and they do not include any individual case reports or descriptions of clinical features. They do mention specific symptoms that were common among the group, which are described below.

Cases from this article could not be coded for inclusion in the statistical analyses.

- “In the patient population examined, symptoms included **headaches**, **visual problems**, and **speech difficulty**. Neurological findings included **memory loss**, **ataxia or tandem gait**, **diminished tendon reflexes,** and **loss of pin-prick sensation**.” (212)

Casson, Siegel, Sham, Campbell, Tarlau, & DiDomenico (1984) (18)

Number of Cases Described in the Article: 18*
Cases with Anger Control Problems: None of the cases
Cases with Depression: None of the cases
Cases with Suicidality: None of the cases
Cases with Anxiety (Nervousness, Fear, PTSD, Obsessive-Compulsive, Worry, "Neuroses"): None of the cases
Cases with Paranoia/Suspiciousness: None of the cases
Cases with Personality/Demeanor Change: None of the cases
Cases with Substance Use: None of the cases
Cases with Cognitive Change: Cases 1-10, & 12-15; None: Cases 16-18; Unknown: Case 11
Cases Presented as Psychiatric (e.g., depression and anxiety), not Neurological: 0
Cases with Headaches: Not mentioned for any cases
Type of Headaches Described: Not applicable
Cases Described as Having a Progressive Course: Not mentioned for any cases
Cases Described as Having a Non-Progressive Course: Not mentioned for any cases
Unable to be Classified as Having Progressive or Non-Progressive Course: Cases 1-18
Delayed Onset: Not mentioned for any of the cases
Number of Cases with Autopsy Performed: 0
*The authors present the findings from the neurological examinations, EEGs, head CT scans, and neuropsychological testing of 18 former and active boxers (13 former, 5 active). The boxers had not retired from boxing for medical, neurological, or psychiatric reasons and had no known history of neurological, psychiatric, or serious mental illness, or known history of drug or alcohol abuse. None had any history of aberrant behavior of psychiatric disturbance. The authors only report individual clinical features in Table 2 (2664-2665). They refer to them in the context of the group in text. Although they do not provide any individual case reports or descriptions of clinical features, the relevant clinical features specific to each case, as reported/written in Table 2, are presented below. Impairment index was calculated by the authors as a ratio of abnormal neuropsychological test scores over the number of tests administered. The article was coded in the summary table based on the findings of the overall sample and the conclusions and opinions presented by the authors.

Case 1 (Age: 37; Sport: Boxing)
Age started: Not mentioned
Age retired: Not mentioned, but at least one year before the present study (inclusion criteria for sample)
Length of career: 12 years Age of symptom onset: Not mentioned
Delay between retirement and symptom onset (Age of symptom onset – Age retired): Not mentioned

- Times Knocked Out: 0; Episodes of Amnesia: 0
- Neurological Examination: **Impaired recent memory**
- Trail Making Test: Normal
- Digit Symbol Test: Normal
- Wechsler Memory Test
  - Verbal immediate: Abnormal
  - Verbal delayed: Abnormal
  - Visual immediate: Abnormal
  - Visual delayed: Abnormal
- Bender Gestalt Test
  - Standard administration: Normal
  - 5-s recall administration: Abnormal
- Impairment Index: 0.50

Case 2 (Age: 42; Sport: Boxing)
Age started: Not mentioned
Age retired: Not mentioned, but at least one year before the present study (inclusion criteria for sample)
Length of career: 10 years
Age of symptom onset: Not mentioned
Delay between retirement and symptom onset (Age of symptom onset – Age retired): Not mentioned

- Times Knocked Out: 1; Episodes of Amnesia: 0
- Neurological Examination: **Mild OMS** (Organic Mental Syndrome – disorientation, confusion, and memory loss)
- Trail Making Test: Abnormal
- Digit Symbol Test: Abnormal
- Wechsler Memory Test
    - Verbal immediate: Abnormal
    - Verbal delayed: Abnormal
    - Visual immediate: Abnormal
    - Visual delayed: Abnormal
- Bender Gestalt Test
    - Standard administration: Abnormal
    - 5-s recall administration: Abnormal
- Impairment Index: 1.00

Case 3 (Age: 33; Sport: Boxing)
Age started: Not mentioned
Age retired: Not mentioned, but at least one year before the present study (inclusion criteria for sample)
Length of career: 13 years
Age of symptom onset: Not mentioned
Delay between retirement and symptom onset (Age of symptom onset – Age retired): Not mentioned

- Times Knocked Out: 2; Episodes of Amnesia: 0
- Neurological Examination: Normal
- Trail Making Test: Normal
- Digit Symbol Test: Normal
- Wechsler Memory Test
    - Verbal immediate: Abnormal
    - Verbal delayed: Abnormal
    - Visual immediate: Normal
    - Visual delayed: Normal
- Bender Gestalt Test
    - Standard administration: Normal
    - 5-s recall administration: Abnormal
- Impairment Index: 0.38

Case 4 (Age: 37; Sport: Boxing)
Age started: Not mentioned
Age retired: Not mentioned, but at least one year before the present study (inclusion criteria for sample)
Length of career: 10 years
Age of symptom onset: Not mentioned
Delay between retirement and symptom onset (Age of symptom onset – Age retired): Not mentioned

- Times Knocked Out: 0; Episodes of Amnesia: 0
- Neurological Examination: Normal
- Trail Making Test: Normal

- Digit Symbol Test: Abnormal
- Wechsler Memory Test
    - Verbal immediate: Abnormal
    - Verbal delayed: Abnormal
    - Visual immediate: Abnormal
    - Visual delayed: Abnormal
- Bender Gestalt Test
    - Standard administration: Abnormal
    - 5-s recall administration: Abnormal
- Impairment Index: 0.88

Case 5 (Age: 39; Sport: Boxing)
Age started: Not mentioned
Age retired: Not mentioned, but at least one year before the present study (inclusion criteria for sample)
Length of career: 14 years
Age of symptom onset: Not mentioned
Delay between retirement and symptom onset (Age of symptom onset – Age retired): Not mentioned

- Times Knocked Out: 1; Episodes of Amnesia: 2
- Neurological Examination: Normal
- Trail Making Test: Abnormal
- Digit Symbol Test: Abnormal
- Wechsler Memory Test
    - Verbal immediate: Abnormal
    - Verbal delayed: Abnormal
    - Visual immediate: Abnormal
    - Visual delayed: Abnormal
- Bender Gestalt Test
    - Standard administration: Normal
    - 5-s recall administration: Abnormal
- Impairment Index: 0.88

Case 6 (Age: 58; Sport: Boxing)
Age started: Not mentioned
Age retired: Not mentioned, but at least one year before the present study (inclusion criteria for sample)
Length of career: 8 years
Age of symptom onset: Not mentioned
Delay between retirement and symptom onset (Age of symptom onset – Age retired): Not mentioned

- Times Knocked Out: 2; Episodes of Amnesia: 2
- Neurological Examination: Normal (**cortical release phenomena**)
- Trail Making Test: Normal
- Digit Symbol Test: Normal
- Wechsler Memory Test
    - Verbal immediate: Abnormal
    - Verbal delayed: Abnormal
    - Visual immediate: Abnormal
    - Visual delayed: Abnormal
- Bender Gestalt Test
    - Standard administration: Normal
    - 5-s recall administration: Abnormal
- Impairment Index: 0.75

Case 7 (Age: 46; Sport: Boxing)
Age started: Not mentioned
Age retired: Not mentioned, but at least one year before the present study (inclusion criteria for sample)
Length of career: 22 years
Age of symptom onset: Not mentioned
Delay between retirement and symptom onset (Age of symptom onset – Age retired): Not mentioned

- Times Knocked Out: 4; Episodes of Amnesia: 0
- Neurological Examination: **Dysarthria nystagmus**
- Trail Making Test: Abnormal
- Digit Symbol Test: Normal
- Wechsler Memory Test
  - Verbal immediate: Abnormal
  - Verbal delayed: Abnormal
  - Visual immediate: Abnormal
  - Visual delayed: Abnormal
- Bender Gestalt Test
  - Standard administration: Abnormal
  - 5-s recall administration: Abnormal
- Impairment Index: 0.88

Case 8 (Age: 30; Sport: Boxing)
Age started: Not mentioned
Age retired: Not mentioned, but at least one year before the present study (inclusion criteria for sample)
Length of career: 15 years
Age of symptom onset: Not mentioned
Delay between retirement and symptom onset (Age of symptom onset – Age retired): Not mentioned

- Times Knocked Out: 0; Episodes of Amnesia: 0
- Neurological Examination: Normal
- Trail Making Test: Abnormal
- Digit Symbol Test: Abnormal
- Wechsler Memory Test
  - Verbal immediate: Abnormal
  - Verbal delayed: Abnormal
  - Visual immediate: Abnormal
  - Visual delayed: Abnormal
- Bender Gestalt Test
  - Standard administration: Normal
  - 5-s recall administration: Abnormal
- Impairment Index: 0.88

Case 9 (Age: 37; Sport: Boxing)
Age started: Not mentioned
Age retired: Not mentioned, but at least one year before the present study (inclusion criteria for sample)
Length of career: 5 years
Age of symptom onset: Not mentioned
Delay between retirement and symptom onset (Age of symptom onset – Age retired): Not mentioned

- Times Knocked Out: 2; Episodes of Amnesia: 5
- Neurological Examination: Normal
- Trail Making Test: Abnormal

- Digit Symbol Test: Normal
- Wechsler Memory Test
  - Verbal immediate: Abnormal
  - Verbal delayed: Abnormal
  - Visual immediate: Normal
  - Visual delayed: Normal
- Bender Gestalt Test
  - Standard administration: Abnormal
  - 5-s recall administration: Abnormal
- Impairment Index: 0.62

Case 10 (Age: 55; Sport: Boxing)
Age started: Not mentioned
Age retired: Not mentioned, but at least one year before the present study (inclusion criteria for sample)
Length of career: 5 years
Age of symptom onset: Not mentioned
Delay between retirement and symptom onset (Age of symptom onset – Age retired): Not mentioned

- Times Knocked Out: 0; Episodes of Amnesia: 0
- Neurological Examination: **Mild OMS** (Organic Mental Syndrome – disorientation, confusion, and memory loss)**, right Babinski**
- Trail Making Test: Abnormal
- Digit Symbol Test: Abnormal
- Wechsler Memory Test
  - Verbal immediate: Abnormal
  - Verbal delayed: Abnormal
  - Visual immediate: Abnormal
  - Visual delayed: Abnormal
- Bender Gestalt Test
  - Standard administration: Abnormal
  - 5-s recall administration: Abnormal
- Impairment Index: 1.00

Case 11 (Age: 40; Sport: Boxing)
Age started: Not mentioned
Age retired: Not mentioned, but at least one year before the present study (inclusion criteria for sample)
Length of career: 20 years
Age of symptom onset: Not mentioned
Delay between retirement and symptom onset (Age of symptom onset – Age retired): Not mentioned

- Times Knocked Out: 3; Episodes of Amnesia: 4
- Neurological Examination: Normal
- Trail Making Test: Abnormal
- Digit Symbol Test: Normal
- Wechsler Memory Test
  - Verbal immediate: Not administered
  - Verbal delayed: Not administered
  - Visual immediate: Not administered
  - Visual delayed: Not administered
- Bender Gestalt Test
  - Standard administration: Normal
  - 5-s recall administration: Abnormal

- Impairment Index: 0.50

Case 12 (Age: 60; Sport: Boxing)
Age started: Not mentioned
Age retired: Not mentioned, but at least one year before the present study (inclusion criteria for sample)
Length of career: 15 years
Age of symptom onset: Not mentioned
Delay between retirement and symptom onset (Age of symptom onset – Age retired): Not mentioned

- Times Knocked Out: 0; Episodes of Amnesia: 0
- Neurological Examination: **Mild OMS** (Organic Mental Syndrome – disorientation, confusion, and memory loss)
- Trail Making Test: Abnormal
- Digit Symbol Test: Abnormal
- Wechsler Memory Test
  - Verbal immediate: Not administered
  - Verbal delayed: Not administered
  - Visual immediate: Not administered
  - Visual delayed: Not administered
- Bender Gestalt Test
  - Standard administration: Abnormal
  - 5-s recall administration: Abnormal
- Impairment Index: 1.00

Case 13 (Age: 27; Sport: Boxing)
Age started: Not mentioned
Age retired: Not mentioned, but at least one year before the present study (inclusion criteria for sample)
Length of career: 6 years
Age of symptom onset: Not mentioned
Delay between retirement and symptom onset (Age of symptom onset – Age retired): Not mentioned

- Times Knocked Out: 0; Episodes of Amnesia: 0
- Neurological Examination: Normal
- Trail Making Test: Abnormal
- Digit Symbol Test: Abnormal
- Wechsler Memory Test
  - Verbal immediate: Abnormal
  - Verbal delayed: Abnormal
  - Visual immediate: Abnormal
  - Visual delayed: Abnormal
- Bender Gestalt Test
  - Standard administration: Abnormal
  - 5-s recall administration: Abnormal
- Impairment Index: 1.00

Case 14 (Age: 24; Sport: Boxing)
Age started: Not mentioned
Age retired: Still active
Length of career: 8 years
Age of symptom onset: Not mentioned
Delay between retirement and symptom onset (Age of symptom onset – Age retired): Not mentioned

- Times Knocked Out: 0; Episodes of Amnesia: 0

- Neurological Examination: Normal
- Trail Making Test: Abnormal
- Digit Symbol Test: Normal
- Wechsler Memory Test
    - Verbal immediate: Abnormal
    - Verbal delayed: Abnormal
    - Visual immediate: Abnormal
    - Visual delayed: Abnormal
- Bender Gestalt Test
    - Standard administration: Abnormal
    - 5-s recall administration: Abnormal
- Impairment Index: 0.88

Case 15 (Age: 25; Sport: Boxing)
Age started: Not mentioned
Age retired: Still active
Length of career: 6 years
Age of symptom onset: Not mentioned
Delay between retirement and symptom onset (Age of symptom onset – Age retired): Not mentioned

- Times Knocked Out: 1; Episodes of Amnesia: 0
- Neurological Examination: Normal
- Trail Making Test: Normal
- Digit Symbol Test: Normal
- Wechsler Memory Test
    - Verbal immediate: Abnormal
    - Verbal delayed: Abnormal
    - Visual immediate: Abnormal
    - Visual delayed: Abnormal
- Bender Gestalt Test
    - Standard administration: Normal
    - 5-s recall administration: Abnormal
- Impairment Index: 0.62

Case 16 (Age: 23; Sport: Boxing)
Age started: Not mentioned
Age retired: Still active
Length of career: 4 years
Age of symptom onset: Not mentioned
Delay between retirement and symptom onset (Age of symptom onset – Age retired): Not mentioned

- Times Knocked Out: 2; Episodes of Amnesia: 1
- Neurological Examination: Normal
- Trail Making Test: Not administered
- Digit Symbol Test: Not administered
- Wechsler Memory Test
    - Verbal immediate: Abnormal
    - Verbal delayed: Abnormal
    - Visual immediate: Normal
    - Visual delayed: Normal
- Bender Gestalt Test
    - Standard administration: Normal

    - 5-s recall administration: Normal
- Impairment Index: 0.33

Case 17 (Age: 18; Sport: Boxing)
Age started: Not mentioned
Age retired: Still active
Length of career: ¼ year
Age of symptom onset: Not mentioned
Delay between retirement and symptom onset (Age of symptom onset – Age retired): Not mentioned

- Times Knocked Out: 0; Episodes of Amnesia: 0
- Neurological Examination: Normal
- Trail Making Test: Not administered
- Digit Symbol Test: Not administered
- Wechsler Memory Test
    - Verbal immediate: Abnormal
    - Verbal delayed: Abnormal
    - Visual immediate: Normal
    - Visual delayed: Normal
- Bender Gestalt Test
    - Standard administration: Normal
    - 5-s recall administration: Normal
- Impairment Index: 0.33

Case 18 (Age: 18; Sport: Boxing)
Age started: Not mentioned
Age retired: Still active
Length of career: 1 year Age of symptom onset: Not mentioned
Delay between retirement and symptom onset (Age of symptom onset – Age retired): Not mentioned

- Times Knocked Out: 0; Episodes of Amnesia: 0
- Neurological Examination: Normal
- Trail Making Test: Not administered
- Digit Symbol Test: Not administered
- Wechsler Memory Test
    - Verbal immediate: Abnormal
    - Verbal delayed: Abnormal
    - Visual immediate: Normal
    - Visual delayed: Normal
- Bender Gestalt Test
    - Standard administration: Normal
    - 5-s recall administration: Normal
- Impairment Index: 0.33

Roberts, Whitwell, Acland, & Bruton (1990) (19)

Number of Cases Described in the Article: 1
Anger Control Problems: Not mentioned
Depression: Not mentioned
Anxiety (Nervousness, Fear, PTSD, Obsessive-Compulsive, Worry, "Neuroses"): Not mentioned
Suicidality: Not mentioned
Paranoia/Suspiciousness: Not mentioned
Personality/Demeanor Change: Not mentioned
Substance Use: Not Mentioned
Cognitive Change: Yes
Case Presented as Psychiatric (e.g., depression and anxiety), not Neurological: No
Headaches: Not mentioned
Type of Headaches Described: Not applicable
Progressive Course: No
Non-Progressive Course: No
Unable to be Classified as Having Progressive or Non-Progressive Course: Yes*
Delayed Onset Not Applicable
Autopsy Performed: Yes

*Note: The authors appear to believe that "punch drunk syndrome" (dementia pugilistica) is progressive, as they state that it is "a condition characterized by damage to the pyramidal, extrapyramidal, and cerebellar systems and by progression from affective disturbance and memory loss, through psychosis, to progressive dementia." However, the clinical description of this case is so limited, and there is not enough evidence to clearly classify this specific case as progressive or non-progressive.

Case 1 (Age: 76; Gender: Female; Physical Abuse Victim)
Age of first exposure: Not mentioned
Age of last exposure: Not mentioned
Duration of physical abuse: Not specified, but husband had been violent for many years (see below)
Age of symptom onset: Not mentioned
Delay between last exposure and symptom onset (Age of symptom onset – Age retired): Not applicable

- "A 76-year-old woman was admitted to hospital unconscious after being found at home with multiple injuries. She had rib fractures, multiple bruises and abrasions to the head, and **signs of left-sided weakness**. She had a history of a stroke and **had become demented over the past few years, this manifesting predominantly as memory loss and mental confusion**. Relatives told us that **her husband had been violent towards her for many years**, particularly in relation to his drinking, and the patient had often been seen with cuts and bruises." (918)
- "The appearance of the brain of this repeatedly battered woman resembled that seen in dementia pugilistica." (919)
- "Our case helps to fill the gap in the evidence for the concept that head injury can be followed by **Alzheimer-type degeneration**." (919)

Hoff, Knabe, Bovier, & Bouras (1991) (20)

Number of Cases Described in the Article: 1
Anger Control Problems: Not Mentioned
Depression: Not Mentioned
Anxiety (Nervousness, Fear, PTSD, Obsessive-Compulsive, Worry, "Neuroses"): Not Mentioned
Suicidality: Not Mentioned
Case Presented as Psychiatric (e.g., depression and anxiety), not Neurological: No
Paranoia/Suspiciousness: Not Mentioned
Personality/Demeanor Change: Not Mentioned
Substance Use: Not Mentioned
Cognitive Change: Unknown
Headaches: Not Mentioned
Type of Headaches Described: Not applicable
Progressive Course: Yes   Non-Progressive Course: No
Unable to be Classified as Having Progressive or Non-Progressive Course: No
Delayed Onset: Not Mentioned
Autopsy Performed: Yes
Note: This article is heavily focused on neuropathology with no mention of the clinical features of traumatic encephalopathy. We included descriptions of the clinical features of this case. However, they all seem to be due to the patient's severe autism with self-injury behavior (SIB). The authors provide evidence that the neurofibrillary tangles (NFTs) in her brain had a similar distribution to the pattern observed in cases of dementia pugilistica (324).

Case 1 (Age: 24; Sex: Female; Autism diagnosis with head banging and self-injury behavior)
Age of first head banging behavior: 7 years
Age of last head banging behavior: Not mentioned
Duration of head banging: Not mentioned
Age of symptom onset: Not mentioned
Delay between last head banging behavior and symptom onset (Age of symptom onset – Age retired): Not mentioned

- "A psychological evaluation showed a 6-month mental retardation. At this time, the first symptoms of infantile autism were observed…" (321)
- "However, when she was 7 years old, she began to display a very serious form of SIB including head-hiting, head-banging, eye-gouging, and self-biting. She became blind as a bilateral detachment of the retina and dullness of the cornea resulted from repeated head and eye trauma. She was later hospitalized in the psychiatric clinic where she stayed until her death. The next 14 years in psychiatric care were characterized by continuous SIB, particularly directed at her head, which she knocked incessantly against the walls, bed sides or persons." (322)
- "She also used to pull out her hair and scream for hours. She was apparently completely insensitive to pain. She played with a few object and could not tolerate changes in her daily schedule and environment (whenever changes occurred, she would immediately display SIB and scream for hours)." (322)
- "All intended therapeutic measures remained without notable effect, including intensive behavior therapy and psychotropic drugs. She progressively lost her ability to walk and stopped using the few words she had learned." (322)
- "A neurological examination a few days before her death revealed no sign of cerebral or neuromuscular palsy." (322)
- "Moreover, chronic SIB may cause long-term brain lesions which could be related to the **progressive** deterioration observed in our patient." (325)

Hof, Bouras, Buée, Delacourte, Perl, & Morrison (1992) (21)

Number of Cases Described in the Article: 3
Cases with Anger Control Problems: Not mentioned for any cases
Cases with Depression: Cases 1, 2, & 3
Cases with Suicidality: Not mentioned for any cases
Cases with Anxiety (Nervousness, Fear, PTSD, Obsessive-Compulsive, Worry, “Neuroses”): Not mentioned for any cases
Cases with Paranoia/Suspiciousness: Not mentioned for any cases
Cases with Personality/Demeanor Change: Not mentioned for any cases
Cases with Substance Use: Not mentioned for any cases
Cases with Cognitive Change: Cases 1, 2, & 3
Cases Presented as Psychiatric (e.g., depression and anxiety), not Neurological: 0
Cases with Headaches: Not mentioned for any cases
Type of Headaches Described: Not applicable
Cases Described as Having a Progressive Course: 0
Cases Described as Having a Non-Progressive Course: 0
Unable to be Classified as Having Progressive or Non-Progressive Course: Cases 1, 2, & 3
Delayed Onset: Not mentioned for any cases
Number of Cases with Autopsy Performed: 3

Note: The authors briefly present some of the clinical features of three cases (Case 1 was previously reported by Constantinidis & Tissot (1967)) of former professional boxers with dementia pugilistica. However, they present the clinical features as a group in the Materials and Methods section and say that all three boxers demonstrated these symptoms. We included this small description below. It is limited beyond merely listing symptoms. In other words, it does not give much insight into the development of these symptoms, their severity, and their impact relative to each other. Additionally, the introduction, results, and discussion sections are focused on neuropathology differences between dementia pugilistica and Alzheimer’s disease, so there is little other information to use for coding in the Summary table. However, the article was coded in the Summary table. All four major reviews included the two additional cases (Cases 2 and 3).

Cases 1-3 (Case 1 Age: 58; Case 2 Age: 63; Case 3 Age: 69; Sport: Boxing)
Ages started: Not mentioned
Ages retired: Not mentioned
Length of careers: > 25 years for Cases 1-3
Ages of symptom onset: Not mentioned
Delays between retirement and symptom onset (Age of symptom onset – Age retired): Not mentioned

- “The clinical presentation of all three boxers was consistent with the diagnosis of dementia pugilistica and revealed **severe memory disturbances**, **impairment of judgement** and **abstract thinking**, **temporospatial disorientation**, and **dysphoria**, combined with a **constellation of neurological symptoms** including to various degrees **extrapyramidal rigidity, resting tremor, gait disturbances, hypomimia and dysarthria.**” (24)

Jordan, Kanik, Horwich, Sweeney, Relkin, Petito, & Gandy (1995) (22)

Number of Cases Described in the Article: 1
Anger Control Problems: Not mentioned
Depression: Not mentioned
Anxiety (Nervousness, Fear, PTSD, Obsessive-Compulsive, Worry, “Neuroses”): Not mentioned
Suicidality: Not mentioned
Paranoia/Suspiciousness: Not mentioned
Personality/Demeanor Change: Not mentioned
Substance Use: Not mentioned
Cognitive Change: Yes
Case Presented as Psychiatric (e.g., depression and anxiety), not Neurological: No
Headaches: Not mentioned
Type of Headaches Described: Not applicable
Progressive Course: Yes
Non-Progressive Course: No
Unable to be Classified as Having Progressive or Non-Progressive Course: No
Delayed Onset: Yes
Autopsy Performed: Yes

Case 1 (Age: 71, Sport: Boxing)
Age started: Not mentioned
Age retired: Not mentioned
Length of career: 11 years
Age of symptom onset: Not mentioned
Delay between retirement and symptom onset (Stated in article): 37 years

- “He enjoyed a career of 11 years of professional boxing that included 83 fights, of which only 16 were losses (3 of these were knockouts).” (698)
- “A **neurological syndrome** of **progressive cognitive decline began 37 years after his last bout (10 years antemortem),** and **an acute right hemiplegia developed 8 years antemortem**, **following which he no longer recognized his family**. Six weeks antemortem, he suffered a fall and died following two successive left parietooccipital cerebral hemorrhages.” (698)

Geddes, Vowles, Robinson, & Sutcliffe (1996) (23)

Number of Cases Described in the Article: 1
Anger Control Problems: Not mentioned
Depression: Not mentioned
Anxiety (Nervousness, Fear, PTSD, Obsessive-Compulsive, Worry, "Neuroses"): Not mentioned
Suicidality: Not mentioned
Paranoia/Suspiciousness: Not mentioned
Personality/Demeanor Change: Not mentioned
Substance Use: Not Mentioned
Cognitive Change: Unknown
Case Presented as Psychiatric (e.g., depression and anxiety), not Neurological: No
Headaches: Not mentioned
Type of Headaches Described: Not applicable
Progressive Course: No
Non-Progressive Course: No
Unable to be Classified as Having Progressive or Non-Progressive Course: Yes
Delayed Onset: Not applicable
Autopsy Performed: Yes

Note: The author essentially conveys that this case had no clinical symptoms and is described as "asymptomatic." They focus on his neurofibrillary tangles (NFTs) and compare them to NFTs in Alzheimer's disease.

Case 1 (Age: 23; Sport: Boxing)
Age started: 11 years (professional for 4 years)
Age retired: 23 years (death from acute subdural haemmorhage sustained in a boxing match)
Length of career: 12 years
Age of symptom onset: Not applicable
Delay between retirement and symptom onset (Age of symptom onset – Age retired): Not applicable

- "[He] has suffered only one knock-out, in his penultimate contest. He had not previously sustained a serious head injury as a result of a fight, had **no neurological symptoms**, although he was described as being **'somewhat forgetful.'** He had not undergone psychometric testing." (12)
- "During his last contest, however, our subject sustained a severe head injury, as a result he developed an acute subdural haemorrhage with marked swelling of the underlying hemisphere… he died 46 h after admission, despite evacuation of the haematoma." (12-13)

Williams & Tannenberg (1996) (24)

Number of Cases Described in the Article: 1
Anger Control Problems: Yes
Depression: Not mentioned
Anxiety (Nervousness, Fear, PTSD, Obsessive-Compulsive, Worry, "Neuroses"): Yes
Suicidality: Not mentioned
Paranoia/Suspiciousness: Not mentioned
Personality/Demeanor Change: Not mentioned
Substance Use: Yes
Cognitive Change: Yes
Case Presented as Psychiatric (e.g., depression and anxiety), not Neurological: No
Headaches: No (The patient died during the night after earlier complaining of a headache, but headaches are otherwise not mentioned, and headaches do not appear to have been a common or chronic symptom)
Type of Headaches Described: Not applicable
Progressive Course: No
Non-Progressive Course: No
Unable to be Classified as Having Progressive or Non-Progressive Course: Yes
Delayed Onset: Not mentioned
Autopsy Performed: Yes

Case 1 (Age: 33; Sport: Circus clown / dwarf throwing)
Age started: Not mentioned
Age retired: Not mentioned
Length of career: 15 years in the circus, 8-10 years in dwarf throwing
Age of symptom onset: Not mentioned
Delay between retirement and symptom onset (Age of symptom onset – Age retired): Not mentioned, but symptom onset occurred prior to retirement from dwarf throwing, as he was still participating at time of death.

- "A 33-yr-old male achondroplastic dwarf died during the night after earlier complaining of a headache. He was known to abuse alcohol (100 to 400g alcohol per day) and, according to his mother, he had been diagnosed as having cirrhosis." (102)
- "He was a frequent attender at hospital with [alcohol] withdrawal seizures. The hospital notes recorded **aggression**, **poor concentration**, **impatience, panic symptoms** and **blackout**. Clinical examination had revealed **ataxia**, **nystagmus**, **mild Rombergism** and **brisk reflexes** and he had received treatment for Wernicke's encephalopathy. The **clinical problems were attributed to alcohol abuse and dietary indiscretions**. A diagnosis of dementia pugilistica had not been considered." (102)
- "Four years prior to his death, he had been sacked for persistent drunkenness from his job as a circus clown. He had worked in the circus 15 years and had apparently been knocked unconscious "a dozen times" during circus routines, without receiving hospitalization." (102)

Note: The authors describe how this case's clinical features were attributed to his alcohol abuse and poor diet at his visits to the hospital. The authors do not mention whether or not they believe his clinical symptoms were due to traumatic encephalopathy or these other factors.

Jordan, Relkin, Ravdin, Jacobs, Bennett, & Gandy (1997) (25)

Number of Cases Described in the Article: 30
Cases with Anger Control Problems: Not mentioned for any cases
Cases with Depression: Case 29; Not Mentioned: Cases 1-28 & 30
Cases with Suicidality: Not mentioned for any cases
Cases with Anxiety (Nervousness, Fear, PTSD, Obsessive-Compulsive, Worry, “Neuroses”): Not mentioned for any cases
Cases with Paranoia/Suspiciousness: Not mentioned for any cases
Cases with Personality/Demeanor Change: Cases 19, 25, & 26; Not Mentioned: Cases 1-18, 20-24, & 27-30
Cases with Substance Use: Cases 10, 11, 13, 14, 25, & 26; Not Mentioned: 1-9, 12, 15-24, & 27-30
Cases with Cognitive Change: Cases 1, 10, 13-15, 17-19, 22, 28, & 29; None: Cases 2, 4, 6-9, 11, 12, 16, 20, 21, 23, 24, 26, 27, & 30; Unknown: Cases 3, 5, & 25
Cases Presented as Psychiatric (e.g., depression and anxiety), not Neurological: 0
Cases with Headaches: Not mentioned for any cases
Type of Headaches Described: Not applicable
Cases Described as Having a Progressive Course: 0
Cases Described as Having a Non-Progressive Course: 0
Unable to be Classified as Having Progressive or Non-Progressive Course: 30*
Delayed Onset: Not mentioned for any cases
Number of Cases with Autopsy Performed: 0

*Note: The authors provide a list of neurologic findings and other clinical features of disease for each case. However, they do not provide narrative descriptions (i.e., case reports), time of symptom onset, or any information regarding the progression of symptomology. Therefore, we are unable to classify each specific case as having a progressive or non-progressive course.

The authors do not provide individual narrative case reports in text. However, they do list clinical features of the 30 cases in Table 3 (138). Although limited relative to other narrative descriptions in this document, relevant individual clinical features of each case are presented below. The authors summarize the clinical features of the cases in text, which informed coding in the summary table, in addition to the quotations provided below. The age at which the boxers started boxing, retired, and experienced symptom onset were not reported. Therefore, this information is not included at the beginning of each case. The authors did include each case’s Mini-Mental State Examination (MMSE) score, which quantifies cognitive functioning; their Chronic Brain Injury (CBI) scale, which quantifies clinical findings of motor, cognitive, and psychiatric deficits; and the authors’ chronic traumatic brain injury (CTBI) classification in Table 3. These variables are provided at the beginning of each case instead. On the MMSE, a score of 28-30 indicates normal cognitive functioning, a score of 20-27 indicates mild impairment, a score of 10-19 indicates moderate impairment, and a score of ≤9 indicates severe impairment. The CBI ranges from 0-9. A score of 0 is classified as normal, a score of 1-2 indicates mild impairment, a score of 3-4 indicates moderate impairment, and a score of >4 indicates severe impairment.

Case 1 (Age: 70; Sport: Boxing)
MMSE Score: 3
CBI Score: 8
CTBI Classification: Probable

- “Neurologic Examination: **Severe dementia**; **severe spasticity**; **agitation**; **dysarthria**; **gait ataxia**” (138)

Case 2 (Age: 25; Sport: Boxing)
MMSE Score: 28
CBI Score: 0
CTBI Classification: Normal

- "Neurologic Examination: Normal" (138)

Case 3 (Age: 48; Sport: Boxing)
MMSE Score: 28
CBI Score: 2
CTBI Classification: Probable

- "Neurologic Examination: **Posturing on stressed gait; decreased simple and complex attention; mild dysarthria**" (138)

Case 4 (Age: 29; Sport: Boxing)
MMSE Score: 29
CBI Score: 0
CTBI Classification: Normal

- "Neurologic Examination: Normal" (138)

Case 5 (Age: 31; Sport: Boxing)
MMSE Score: N/A
CBI Score: 1
CTBI Classification: Probable

- "Neurologic Examination: **Difficulty with Tandem gait**" (138)
- "Comment: Complained of **difficulties with balance**" (138)

Case 6 (Age: 29; Sport: Boxing)
MMSE Score: 30
CBI Score: 0
CTBI Classification: Normal

- "Neurologic Examination: Normal" (138)

Case 7 (Age: 57; Sport: Boxing)
MMSE Score: 29
CBI Score: 0
CTBI Classification: Normal

- "Neurologic Examination: Normal" (138)

Case 8 (Age: 57; Sport: Boxing)
MMSE Score: 28
CBI Score: 0
CTBI Classification: Normal

- "Neurologic Examination: Normal" (138)

Case 9 (Age: 36; Sport: Boxing)
MMSE Score: 30
CBI Score: 0
CTBI Classification: Normal

- "Neurologic Examination: Normal" (138)

Case 10 (Age: 59; Sport: Boxing)
MMSE Score: 27
CBI Score: 2
CTBI Classification: Possible

- “Neurologic Examination: **Mild dysarthria**” (138)
- “Comment: Regular alcohol use” (138)

Case 11 (Age: 39; Sport: Boxing)
MMSE Score: 30
CBI Score: 0
CTBI Classification: Normal

- “Neurologic Examination: Normal” (138)
- “Comment: Occasional recreational drug use; regular alcohol use” (138)

Case 12 (Age: 70; Sport: Boxing)
MMSE Score: 27
CBI Score: 1
CTBI Classification: Possible

- “Neurologic Examination: Normal” (138)

Case 13 (Age: 65; Sport: Boxing)
MMSE Score: 25
CBI Score: 2
CTBI Classification: Possible

- “Neurologic Examination: **Difficulty with tandem gait**; **positive Romberg sign**” (138)
- “Comment: History of alcohol abuse” (138)

Case 14 (Age: 76; Sport: Boxing)
MMSE Score: 24
CBI Score: 2
CTBI Classification: Possible

- “Neurologic Examination: **Mild-moderate memory impairment**” (138)
- “Comment: Regular alcohol use” (138)

Case 15 (Age: 69; Sport: Boxing)
MMSE Score: 22
CBI Score: 4
CTBI Classification: Probable

- “Neurologic Examination: **Severe dysarthria; slight increases tone in lower extremities; ataxic gait; slight dysmetria on left**” (138)

Case 16 (Age: 67; Sport: Boxing)
MMSE Score: 29
CBI Score: 0
CTBI Classification: Normal

- “Neurologic Examination: Normal” (138)

Case 17 (Age: 64; Sport: Boxing)
MMSE Score: 27
CBI Score: 2
CTBI Classification: Possible
- "Neurologic Examination: **Mild hyperreflexia on left; resting tremor** and **mild cogwheel rigidity**" (138)

Case 18 (Age: 37: Sport: Boxing)
MMSE Score: 20
CBI Score: 5
CTBI Classification: Probable
- "Neurologic Examination: **Flat affect with pseudobulbar-type laughing outburst; dysarthria; mild paratonia of right upper extremity; ankle clonus; slowed rapid alternating movements; ataxic gait; memory impairment**" (138)

Case 19 (Age: 41; Sport: Boxing)
MMSE Score: 17
CBI Score: 5
CTBI Classification: Probable
- "Neurologic Examination: **Mild pseudobulbar affect; dysarthria; memory impairment; dysmetria; ataxic gait**" (138)

Case 20 (Age: 35; Sport: Boxing)
MMSE Score: 29
CBI Score: 0
CTBI Classification: Normal
- "Neurologic Examination: Normal" (138)

Case 21 (Age: 64; Sport: Boxing)
MMSE Score: 30
CBI Score: 2
CTBI Classification: Probable
- "Neurologic Examination: **Moderate dysarthria and incoordination; slight increased tone in right lower extremity with wide based ataxic gait; hyperreflexia in right upper extremity**" (138)

Case 22 (Age: 60; Sport: Boxing)
MMSE Score: 27
CBI Score: 2
CTBI Classification: Probable
- "Neurologic Examination: **Difficulty with sharpened Romberg sign**" (138)

Case 23 (Age: 68; Sport: Boxing)
MMSE Score: 29
CBI Score: 1
CTBI Classification: Possible
- "Neurologic Examination: **Mild tremor with titubation**" (138)

Case 24 (Age: 47; Sport: Boxing)
MMSE Score: 30
CBI Score: 1
CTBI Classification: Probable

- “Neurologic Examination: **Mild dysarthria** and **cogwheel rigidity**” (138)

Case 25 (Age: 31; Sport: Boxing)
MMSE Score: 28
CBI Score: 2
CTBI Classification: Possible

- “Neurologic Examination: **Mild dysmetria** and **difficulty with Romberg sign**; **hypomania** and **increased talkativeness**” (138)
- “Comment: One episode of head trauma outside of the ring with loss of consciousness; still boxing; history of drug abuse” (138)
- “One boxer with mild CTBI was diagnosed with bipolar disorder during his boxing career and displayed hypomania and increased talkativeness during the examination.” (139)

Case 26 (Age: 39; Sport: Boxing)
MMSE Score: 29
CBI Score: 3
CTBI Classification: Probable

- “Neurologic Examination: Anisocoria; abducens palsy diplopia on lateral gaze; dysmetria; ataxia; pseudobulbar affect and **behavioral disinhibition**” (138)
- “Comment: History of alcohol abuse; cavum septum on CT” (138)

Case 27 (Age: 30; Sport: Boxing)
MMSE Score: 29
CBI Score: 0
CTBI Classification: Normal

- “Neurologic Examination: Normal”

Case 28 (Age: 64; Sport: Boxing)
MMSE Score: 25
CBI Score: 3
CTBI Classification: Probable

- “Neurologic Examination: **Mild dysarthria; gait ataxia; resting tremor**” (138)

Case 29 (Age: 37; Sport: Boxing)
MMSE Score: 27
CBI Score: 3
CTBI Classification: Probable

- “Neurologic Examination: **Slight increased tone in lower extremities; abnormal sharpened Romberg sign; mild rigidity; mild depression**” (138)
- “Comment: Complained of **memory loss**” (138)

Case 30 (Age: 23; Sport: Boxing)
MMSE Score: 29
CBI Score: 0
CTBI Classification: Normal

- “Neurologic Examination: Normal” (138)

Geddes, Vowles, Nicoll, & Révész (1999) (26)

Number of Cases Described in the Article: 4*
Cases with Anger Control Problems: Not mentioned for any cases
Cases with Depression: Case 2; Not Mentioned: Cases 1, 3, & 4
Cases with Suicidality: Not mentioned for any cases
Cases with Anxiety (Nervousness, Fear, PTSD, Obsessive-Compulsive, Worry, "Neuroses"): Not mentioned for any cases
Cases with Paranoia/Suspiciousness: Case 2; Not Mentioned: Cases 1, 3, & 4
Cases with Personality/Demeanor Change: Case 2; Not Mentioned: Cases 1, 3, & 4
Cases with Substance Use: Not mentioned for any cases
Cases with Cognitive Change: Unknown: Cases 1, 3, & 4; Not Mentioned: Case 2
Cases Presented as Psychiatric (e.g., depression and anxiety), not Neurological: Case 2
Cases with Headaches: Not mentioned for any cases
Type of Headaches Described: Not applicable
Cases Described as Having a Progressive Course: 0
Cases Described as Having a Non-Progressive Course: 0
Unable to be Classified as Having Progressive or Non-Progressive Course: 4*
Delayed Onset: None: Case 2; Not Applicable: Cases 1, 3, & 4
Number of Cases with Autopsy Performed: 3 (Cases 1-3)

*Note: The authors provide a very limited description for each case in terms of their clinical features. The article focuses on neuronal cytoskeletal changes in young adults with history of mild head injury. The case descriptions describe the history of each case, but clinical features are very limited and when they are mentioned, their age of onset and progression are not mentioned. The authors make no reference to these features in any other parts of the article. This article included 5 cases. One of the cases (case #5) has been included in prior reviews of the CTE literature, but it does not appear to be a case of CTE or TES. This was a 23-year-old man who had a personal history of a "severe head injury" from which he was deemed to have made a full recovery. He died from an acute subdural hematoma and brain swelling from a head injury in a football match. This case (case #5) was excluded. Other cases who died due to acute subdural hematoma were included because they had a long history of repetitive neurotrauma (case #1 and #3). It is debatable whether to include case #4 because that person had no sporting history and had lifelong severe intractable epilepsy. We included it in part because past reviewers included it.

Case 1 (Age: 23; Sport: Boxing)
Age started: 11 years (professional at 19)
Age retired: 23 years
Length of career: 12 years (4 professional)
Age of symptom onset: Not Mentioned
Delay between retirement and symptom onset (Age of symptom onset – Age retired): Not applicable

- Case 1 in Geddes et al. (1999) was reported by Geddes et al. (1996)
- "He had no history of a severe head injury during his career, until his final fight, as a result of which he developed an acute subdural haematoma. He died 48 h after the contest, despite neurosurgical intervention." (172)
- There are no additional clinical features presented in Geddes et al. (1999)

Case 2 (Age: 28; Sport: Boxing)
Age started: 16 years
Age retired: 21 years
Length of career: 5 years
Age of symptom onset: 20 years (considering hospitalization for paranoid schizophrenia as first “symptom”)
Delay between end of career and symptom onset (Age of symptom onset – Age retired): -1 year*
*Symptom onset during career

- “**At the age of 20** he was admitted to a **psychiatric hospital** with a diagnosis of **paranoid schizophrenia** which responded to major tranquillisers. He was readmitted with an **acute psychotic illness at the age of 25**, **this type more depressive in nature, and then again 2 years later**. He died unexpectedly the following year during a grand mal seizure. He had no history of a serious head injury during his boxing career.” (172)

Case 3 (Age: 28; Sport: Not applicable; Grand mal epilepsy and autistic head banging)
Age started: Not applicable
Age Retired: Not applicable
Length of career: Not applicable
Age of symptom onset: Not mentioned
Delay between end of career and symptom onset (Age of symptom onset – Age retired): Not applicable

- “A mentally subnormal man of 28, who had been slow to achieve childhood developmental milestones, developed grand mal epilepsy at the age of 7 years, at which time he was also diagnosed to be autistic. There was a long history of head banging, but no episode of severe head injury. He lived his adult life in residential care, and died 2 days after a fall from a first floor window, in which he sustained a skull fracture, extradural and acute subdural haematomas, and large basal frontal contusions.” (172)

Case 4 (Age: 27; Sport: Not applicable; Intractable complex partial seizures with secondary generalized seizures)
Age started: Not applicable
Age Retired: Not applicable
Length of career: Not applicable
Age of symptom onset: Not mentioned
Delay between end of career and symptom onset (Age of symptom onset – Age retired): Not applicable

- “A mentally retarded man of 27 years of age who had suffered from intractable complex partial seizures with secondary generalized seizures. He was born 2 months prematurely, suffered from birth trauma and had his first seizure at the age of 2.5 years. The seizure frequency gradually increased, until in recent years he had both “major attacks” seven to ten times a week in which he dropped heavily to the ground and “minor attacks” which occurred once a week.” (172)

Newell & Drachman (1999) (27)

Number of Cases Described in the Article: 1
Anger Control Problems: Yes
Depression: Not mentioned
Anxiety (Nervousness, Fear, PTSD, Obsessive-Compulsive, Worry, “Neuroses”): Not mentioned
Suicidality: Not mentioned
Paranoia/Suspiciousness: Yes
Personality/Demeanor Change: Yes
Substance Use: Yes
Cognitive Change: Yes
Case Presented as Psychiatric (e.g., depression and anxiety), not Neurological: No (Presented initially as a case of dementia with psychiatric and behavioral problems arising later on)
Headaches: Not mentioned
Type of Headaches Described: Not applicable
Progressive Course: Yes
Non-Progressive Course: No
Unable to be Classified as Having Progressive or Non-Progressive Course: No
Delayed Onset: Yes
Autopsy Performed: Yes

Case 1 (Age: 67; Sport; Boxing)
Age started: Not mentioned
Age retired: Not mentioned
Length of career: 10 years
Age of symptom onset: Likely 64 years (three years before admission; see below)
Delay between retirement and symptom onset: 40 years

- “A 67-year-old, right-handed man was admitted to the hospital because of **increasing dementia**.” (1269)
- “**Three years before admission**, the patient’s **cognitive function began to decline**. **One year later**, a neurologist **diagnosed progressive dementia with parkinsonism**.” (1269)
- “**During the two months before admission**, the patient **became agitated**, and trazodone was prescribed. His family noted **transient periods when he stared into space and did not respond to his surroundings**. He **fell several times**, with **apparent loss of consciousness but no obvious injury**.” (1269)
- “**One week before admission, the patient punched his wife because she would not let him drive. Soon thereafter, he began to look under his bed, locked the doors and windows, and became convinced that the neighbors were trying to steal the family’s car**. Subsequently, he **struck his teenage grandson without provocation and wandered from home**. He was referred to this hospital.” (1269)
- “He **drank alcohol excessively** in his youth but had been sober for several decades.” (1269)
- “The results of a general physical examination were normal.” (1269)
- “On neurologic examination, the patient was **alert but was oriented only to himself**. He believed that he was in his house in the city and that the year was 1970. He **lost concentration when attempting serial tasks**. **Abstract tasks were performed poorly**. He recalled none of three objects at five minutes, even with cues. **Remote autobiographical memories were relatively preserved. Left–right confusion was present**. The patient’s **language was intact**, and phrases were repeated accurately. His speech was **hypophonic**. His **facial expression was reduced**, and he **drooled**. **Cranial nerve functions were preserved**. There was **severe atrophy of the shoulder girdles and arms and legs**. **Diffuse fasciculations and muscle twitches were observed.** M**otor power was normal**. The patient’s **posture was slightly stooped**. His **spontaneous gait was normal, and tandem walking was unsteady**. **No resting tremor or cogwheel rigidity** was detected. His s**ense of joint position and perception of vibration were intact**. The deep-tendon reflexes

were + or ++ and symmetric. **No bradykinesia or postural instability** was evident, and the **glabellar-tap sign and Babinski signs were absent**." (1269)

- "Trazodone was continued, and treatment with risperidone was begun. The patient became less agitated. On the 16th hospital day, he was transferred to a nursing home, where he died suddenly 5 days later." (1270)
- "Dementia… may be **progressive**, as in this case." (1270)
- "This patient appears to have had a **progressive and apparently degenerative dementia**." (1270)
- "**Two years before the patient was hospitalized, a neurologist had diagnosed 'progressive dementia with parkinsonism**.' Neurologic examination at this hospital revealed only minor extrapyramidal features: some reduction of facial expression, mild hypophonia, and a slightly stooped posture, but without marked rigidity, a resting tremor, a gait disorder, or a glabellar sign." (1271)
- "**Abnormal thought and behavior were an important, although not initial, part of the patient's dementia**, with **paranoid ideas**, **loss of inhibition**, and **aggressive acts**." (1271)
- "In this case, the patient's **prominent behavioral problems** were the reason for his prolonged hospitalization and treatment with neuroleptic drugs. A striking feature of this case, with important diagnostic implications, was the **presence of motor neuron disease**, with severe atrophy of both shoulder girdles and distal muscles, and diffuse fasciculations. Muscle strength was reported to be normal, and Babinski signs were absent. Nevertheless, an electromyogram, which is not part of the routine workup for dementia, showed chronic denervation and reinnervation in all muscle groups, a finding consistent with the presence of a 'generalized disorder of motor neurons, their axons, or both.'" (1271)
- "His **symptoms began with dementia**, he **later had mild parkinsonism**, **his behavior became troublesome and disinhibited, and he had considerable evidence of motor neuron disease**." (1272)
- "The **long period of apparently normal functioning after his retirement from boxing** is somewhat unusual." (1273)
- "We **favored the diagnosis of dementia pugilistica**, although the **40-year period of latency between his retirement from boxing and the emergence of his disease was not consistent with this diagnosis.**" (1274)

Appendix A. Cases from the 20th Century with Postmortem Findings

These should not be considered cases of chronic traumatic encephalopathy neuropathologic change, or pathologically confirmed cases of CTE. Rather, they are cases that have postmortem neuropathology described, and that pathology is diverse, some of it relates to aging, some to traumatic brain injury, some to Alzheimer's disease, and some to other causes.

Reference	Case	Post-Mortem
Martland (1928)	2	No
Parker (1934)	1	No
	2	No
	3	No
Critchley (1949)	A	No
	B	No
	C	No
	D	No
	E	No
	F	No
	G	No
Raevuori-Nallinmaa (1950)	1	No
	2	No
Critchley (1957)	2	No
	3	No
	8	No
	9	No
	10	No
	11	No
	14	No
Corsellis & Brierley (1959)	1	Yes
	2	Yes
Neubuerger, Sinton, & Denst (1959)	1	No
	2	Yes
Courville (1962)	1	Yes
Spillane (1962)	1	No
	2	No
	3	No
	4	No
	5	Yes
Mawdsley & Ferguson (1963)	1	No
	2	No
	3	No
	4	No
	5	No
	6	No
	7	No
	8	No
	9	No
	10	Yes
Payne (1968)	1	Yes
	2	Yes
	3	Yes
	4	Yes
	5	Yes
	6	Yes
Roberts (1969)	1	No
	2	No
	3	No
	4	No
	5	No
	6	No
	7	No
	8	No
	9	No
	10	No
	11	No
Johnson (1969)	1	No
	2	No

Reference	Case	Post-Mortem
	3	No
	5	No
	6	No
	7	No
	8	No
	9	No
	10	No
	11	No
	12	No
	13	No
	14	No
	15	No
	16	No
Corsellis, Bruton, & Freeman-Browne (1973)	1	Yes
	2	Yes
	3	Yes
	4	Yes
	5	Yes
	6	Yes
	7	Yes
	8	Yes
	9	Yes
	10	Yes
	11	Yes
	12	Yes
	13	Yes
	14	Yes
	15	Yes
Harvey & Newsom Davis (1974)	1	No
Casson et al. (1982)	1	No
	2	No
	3	No
	4	No
	5	No
	6	No
	7	No
	8	No
	9	No
	10	No
Kaste et al. (1982)	1	No
	2	No
	3	No
	4	No
	5	No
	6	No
	7	No
	8	No
	9	No
	10	No
	11	No
	12	No
	13	No
	14	No
Casson et al. (1984)	1	No
	2	No
	3	No
	4	No
	5	No
	6	No
	7	No
	8	No
	9	No

Reference	Case	Post-Mortem
	10	No
	11	No
	12	No
	13	No
	14	No
	15	No
	16	No
	17	No
	18	No
Roberts et al. (1990)	1	Yes
Hoff et al. (1991)	1	Yes
Hoff et al. (1992)	1	Yes
	2	Yes
	3	Yes
Jordan et al. (1995)	1	Yes
Geddes et al. (1996)	1	Yes
Williams & Tannenberg (1996)	1	Yes
Jordan et al. (1997)	1	No
	2	No
	3	No
	4	No
	5	No
	6	No
	7	No
	8	No
	9	No
	10	No
	11	No
	12	No
	13	No
	14	No
	15	No
	16	No
	17	No
	18	No
	19	No
	20	No
	21	No
	22	No
	23	No
	24	No
	25	No
	26	No
	27	No
	28	No
	29	No
	30	No
Geddes et al. (1999)	1	Yes
	2	Yes
	3	Yes
	4	No
Newell & Drachman (1999)	1	Yes

Appendix B. Reference List

1. Martland HS. Punch drunk. J Am Med Assoc. 1928;91(15):1103-7.
2. Parker HL. Traumatic Encephalopathy (`Punch Drunk') of Professional Pugilists. J Neurol Psychopathol. 1934;15(57):20-8.
3. Critchley M. Punch-drunk syndromes: the chronic traumatic encephalopathy of boxers. In: Maloine, editor. Hommage a Clovis Vincent. Strasbourg: Imprimerie Alascienne; 1949. p. 131-45.
4. Critchley M. Medical aspects of boxing, particularly from a neurological standpoint. Br Med J. 1957;1(5015):357-62.
5. Mawdsley C, Ferguson FR. Neurological Disease in Boxers. Lancet. 1963;2(7312):795-801.
6. Johnson J. Organic psychosyndromes due to boxing. Br J Psychiatry. 1969;115:45-53.
7. Raevuori-Nallinmaa S. [Cerebral injury in boxing]. Duodecim. 1950;66(5):398-403.
8. Corsellis JA, Brierley JB. Observations on the pathology of insidious dementia following head injury. J Ment Sci. 1959;105:714-20.
9. Neubuerger KT, Sinton DW, Denst J. Cerebral atrophy associated with boxing. AMA Arch Neurol Psychiatry. 1959;81(4):403-8.
10. Courville CB. Punch drunk. Its pathogenesis and pathology on the basis of a verified case. Bull Los Angel Neuro Soc. 1962;27:160-8.
11. Spillane JD. Five boxers. Br Med J. 1962;2(5314):1205-10.
12. Payne EE. Brains of boxers. Neurochirurgia (Stuttg). 1968;11(5):173-88.
13. Roberts A. Brain damage in boxers: A study of prevalence of traumatic encephalopathy among ex-professional boxers. London: Pitman Medical Scientific Publishing Co.; 1969.
14. Corsellis JA, Bruton CJ, Freeman-Browne D. The aftermath of boxing. Psychol Med. 1973;3(3):270-303.
15. Harvey PK, Davis JN. Traumatic encephalopathy in a young boxer. Lancet. 1974;2(7886):928-9.
16. Kaste M, Kuurne T, Vilkki J, Katevuo K, Sainio K, Meurala H. Is chronic brain damage in boxing a hazard of the past? Lancet. 1982;2(8309):1186-8.
17. Ross RJ, Cole M, Thompson JS, Kim KH. Boxers--computed tomography, EEG, and neurological evaluation. J Am Med Assoc. 1983;249(2):211-3.
18. Casson IR, Siegel O, Sham R, Campbell EA, Tarlau M, DiDomenico A. Brain damage in modern boxers. J Am Med Assoc. 1984;251(20):2663-7.
19. Roberts GW, Whitwell HL, Acland PR, Bruton CJ. Dementia in a punch-drunk wife. Lancet. 1990;335(8694):918-9.
20. Hof PR, Knabe R, Bovier P, Bouras C. Neuropathological observations in a case of autism presenting with self-injury behavior. Acta Neuropathol. 1991;82(4):321-6.
21. Hof PR, Bouras C, Buee L, Delacourte A, Perl DP, Morrison JH. Differential distribution of neurofibrillary tangles in the cerebral cortex of dementia pugilistica and Alzheimer's disease cases. Acta Neuropathol. 1992;85(1):23-30.
22. Jordan BD, Kanik AB, Horwich MS, Sweeney D, Relkin NR, Petito CK, et al. Apolipoprotein E epsilon 4 and fatal cerebral amyloid angiopathy associated with dementia pugilistica. Ann Neurol. 1995;38(4):698-9.
23. Geddes JF, Vowles GH, Robinson SF, Sutcliffe JC. Neurofibrillary tangles, but not Alzheimer-type pathology, in a young boxer. Neuropathol Appl Neurobiol. 1996;22(1):12-6.
24. Williams DJ, Tannenberg AE. Dementia pugilistica in an alcoholic achondroplastic dwarf. Pathology. 1996;28(1):102-4.
25. Jordan BD, Relkin NR, Ravdin LD, Jacobs AR, Bennett A, Gandy S. Apolipoprotein E epsilon4 associated with chronic traumatic brain injury in boxing. J Am Med Assoc. 1997;278(2):136-40.
26. Geddes JF, Vowles GH, Nicoll JA, Revesz T. Neuronal cytoskeletal changes are an early consequence of repetitive head injury. Acta Neuropathol (Berl). 1999;98(2):171-8.
27. Drachman DA, Newell KL. Case records of the Massachusetts General Hospital. Weekly clinicopathological exercises. Case 12-1999. A 67-year-old man with three years of dementia. N Engl J Med. 1999;340(16):1269-77.
